# Mucospheres produced by a mixotrophic protist impact ocean carbon cycling

**DOI:** 10.1038/s41467-022-28867-8

**Published:** 2022-03-14

**Authors:** Michaela E. Larsson, Anna R. Bramucci, Sinead Collins, Gustaaf Hallegraeff, Tim Kahlke, Jean-Baptiste Raina, Justin R. Seymour, Martina A. Doblin

**Affiliations:** 1 Climate Change Cluster (C3), University of Technology Sydney, PO Box 123, Broadway, NSW 2007 Australia; 2 Institute of Evolutionary Biology, School of Biological Sciences, University of Edinburgh, Edinburgh, UK; 3 Institute for Marine and Antarctic Studies, University of Tasmania, Private Bag 129, Hobart, TAS 7001 Australia; 4 Sydney Institute of Marine Science, Mosman, NSW 2088 Australia

**Keywords:** Carbon cycle, Carbon cycle, Marine biology

## Abstract

Mixotrophic protists (unicellular eukaryotes) that engage in both phototrophy (photosynthesis) and phago-heterotrophy (engulfment of particles)—are predicted to contribute substantially to energy fluxes and marine biogeochemical cycles. However, their impact remains largely unquantified. Here we describe the sophisticated foraging strategy of a widespread mixotrophic dinoflagellate, involving the production of carbon-rich ‘mucospheres’ that attract, capture, and immobilise microbial prey facilitating their consumption. We provide a detailed characterisation of this previously undescribed behaviour and reveal that it represents an overlooked, yet quantitatively significant mechanism for oceanic carbon fluxes. Following feeding, the mucospheres laden with surplus prey are discarded and sink, contributing an estimated 0.17–1.24 mg m^−2^ d^−1^ of particulate organic carbon, or 0.02–0.15 Gt to the biological pump annually, which represents 0.1–0.7% of the estimated total export from the euphotic zone. These findings demonstrate how the complex foraging behaviour of a single species of mixotrophic protist can disproportionally contribute to the vertical flux of carbon in the ocean.

## Abstract (web summary)

Marine microbes govern ocean productivity and biogeochemistry, regulating global climate. Here the authors describe the sophisticated feeding strategy of a mixotrophic dinoflagellate and show how its behaviour impacts the vertical flux of carbon.

## Introduction

The biological pump is a central process in the marine carbon cycle whereby organic particles are exported from the surface to the deep ocean, which ultimately drives carbon sequestration and modulates global climate^1^. Quantification of the biological carbon pump relies on two inextricably linked parameters; (i) net primary production—the process by which carbon is photosynthetically fixed into particulate organic carbon (POC)—and (ii) export efficiency—the flux of POC from the euphotic zone to the deep ocean where the carbon is ultimately sequestered^2^. Despite considerable progress^3^, discrepancies remain between the modelled ocean carbon budget and those derived from field measurements^4^, which are partially ascribable to simplified representations of the ecological processes that contribute to the fluxes^5^. For example, traditional ecosystem models necessarily focus on simulating biogeochemical cycles rather than the organisms that mediate them, with plankton populations represented by broad functional types (e.g., autotrophic phytoplankton and heterotrophic zooplankton), omitting their complex ecological strategies and interactions^6^. This simplification, which is largely driven by a lack of available information, prevents an accurate representation of protists and the impact of their behaviours on biogeochemical cycling^7,8^. Here we describe the sophisticated foraging strategy of a ubiquitous mixotrophic marine protist and reveal how its behaviour can influence the marine carbon cycle.

## Results and discussion

### Identification and distribution of *Prorocentrum* cf. *balticum*

**Fig. 1:**
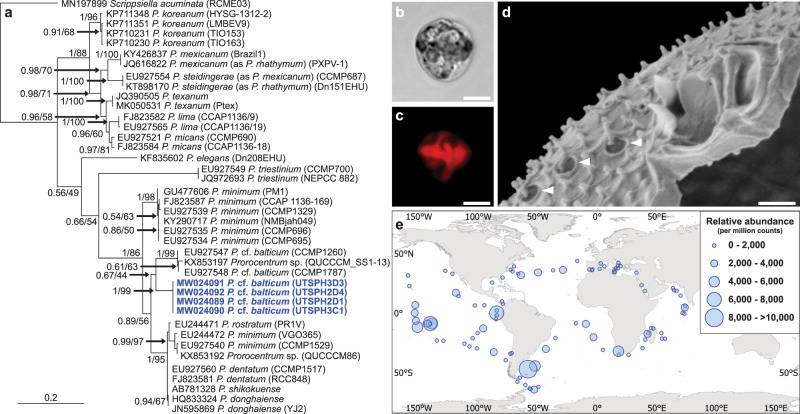
Identification and distribution of *Prorocentrum* cf. *balticum*. **a** Maximum Likelihood phylogenetic tree showing alignment of the internal transcribed spacer (ITS) gene region indicating a clear differentiation of the *P.* cf. *balticum* strains (blue) from described *Prorocentrum* species, and the close relation to undefined strains. Accession numbers, species designation, and strain codes are provided for each sequence and values at the nodes represent Bayesian posterior probability and Maximum Likelihood bootstrap support. **b**, **c** Light microscope images of *P.* cf. *balticum* showing a cell under Differential Interference Contrast (DIC) (**b**) and peripherally located red-autofluorescent chloroplasts (**c**). **d** Scanning Electron Microscope (SEM) image of *P.* cf. *balticum* showing distinctive dual wing-like apical projections and unique large pores with emanating large spines (white arrows). Images **b**, **c** were taken with an Upright Fluorescence Microscope (Nikon Eclipse Ni, Japan), fitted with a Cy5 620/60 nm ex 700/75 nm em filter and a monochrome camera (Nikon DS-Qi2) under 1000× magnification with oil immersion; scale bar = 5 µm. Scale bar in **d** = 0.5 µm. **e** Global biogeography of *P*. cf. *balticum* produced using the relative abundance of 18S rDNA sequences from the *Tara* Oceans amplicon dataset.

Four clonal strains of a small (13–16 µm transdiameter) novel marine dinoflagellate, *Prorocentrum* cf. *balticum* (Fig. 1a–d; Supplementary Figs. 1 and 2; Supplementary Note 1) were isolated from a temperate, continental shelf long-term oceanographic station located 30 km southeast of Sydney, Australia (Port Hacking; 34.120°S, 151.224°E). Analysis of the *Tara* Oceans amplicon dataset (Fig. 1e) revealed an exact match to the 18S rDNA gene sequence of *P*. cf. *balticum* (129 bp) (see “Methods”) that occurred at 96% of stations (95–99) in the surface 5–20 µm size fraction samples (Supplementary Fig. 3), indicating that this species is a cosmopolitan marine eukaryote with the potential to have wide ecological importance. When tested using liquid chromatography-tandem mass spectrometry (LC-MS/MS), our *P*. cf. *balticum* strains and their associated bacterial microbiome (xenic cultures) (see “Methods”) did not produce okadaic acid, dinophysistoxin-1 and −2, or tetrodotoxin. We therefore conclude that this species is unlikely to be associated with human illness or detrimental ecosystem impacts (Supplementary Note 1).

### *Prorocentrum* cf. *balticum* is a constitutive mixotroph

**Fig. 2:**
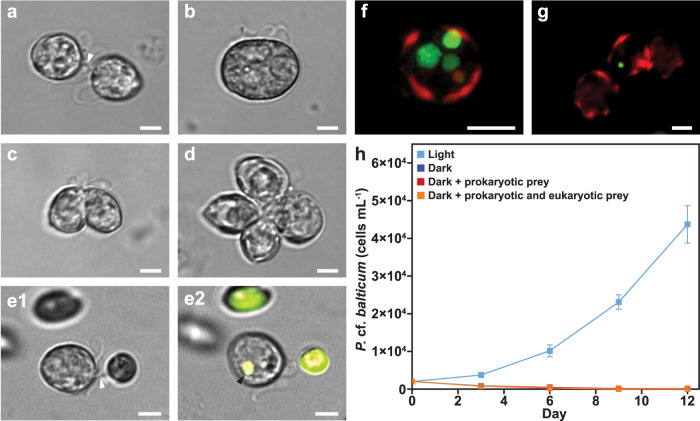
Evidence of sexual reproduction stages and peduncular phago-heterotrophy discovered for *Prorocentrum* cf. *balticum*. Images **a**-**d** show the various stages of sexual reproduction (Supplementary Note 2); extraction of the nucleus through the peduncle (white arrow) (**a**), replication of DNA (**b**), first meiotic division (**c**), and second meiotic division resulting in a tetrad (**d**). **e1,** **e2** Peduncular feeding mechanism (white arrow) used for phago-heterotrophic consumption of a *R. salina* prey cell and the resulting green-fluorescent food vacuole (black arrow). **f**, **g** Fluorescent microscope images showing: the peripheral red-fluorescent chloroplasts and intracellular green-fluorescent food vacuoles following consumption of the cryptophyte *R. salina* (**f**), and an ingested green-fluorescently labelled bacterium (**g**). **h** Growth of *P*. cf. *balticum* in co-culture ± prokaryotic (natural microbiome from xenic culture) and/or eukaryotic prey (*Proteomonas sulcata*) in light and dark conditions. Mean ± standard deviation (*n* = 6). Note that the three treatments in the dark did not grow and are overlapping with low *P*. cf. *balticum* densities. Images **a**–**e**, **g** were taken with an Inverted Fluorescence Microscope (Nikon Eclipse Ti), fitted with Nikon FITC 480/30 nm ex 535/45 nm em and Texas Red 560/40 nm ex 630/60 nm em filters, and a monochrome camera (Nikon DS-QiMc 12 bit) under 200 or 400× magnification. Image **f** was taken with a Nikon AR1 confocal microscope using lasers with 405.6–561.0 nm ex and TRITC 595 nm em, Alex610 700 nm em and TD filters at 1000× magnification with oil immersion and represents a collapsed Z-stack of 26 images (6.5 µm total distance) to clearly show the food vacuoles. Scale bars = 5 µm.

A detailed series of laboratory experiments revealed that *P.* cf. *balticum* utilises a diverse range of metabolic strategies and behaviours to succeed across geographically extensive ocean environments. It uses golden-brown red fluorescent chloroplasts for photosynthesis (Fig. 1c; Supplementary Fig. 4), can divide asexually via desmoschisis when dissolved inorganic nutrients are sufficient, but shifts to sexual reproduction when these are limiting (Supplementary Note 2; Fig. 2a–d; Supplementary Fig. 5), and is mixotrophic^9^, displaying phago-heterotrophic feeding on other small microbes. We observed *P*. cf. *balticum* feeding phago-heterotrophically on the green-fluorescent pigment-containing cryptophyte *Rhodomonas salina* using a short tubular peduncle (Fig. 2e1; Supplementary Movie 1; Supplementary Note 3) and confirmed myzocytosis through visualisation of the resulting green-fluorescent intracellular food vacuoles (Fig. 2e2–f). A broader assessment of prey consumption capabilities confirmed that an additional 11 protistan taxa ranging in size from 3 to 25 µm, including diatoms, dinoflagellates, chlorophytes, cryptophytes, prymnesiophytes, and a eustigmatophyte, were readily consumed by this species, although some larger and thecate dinoflagellates (40–60 µm size) and a rhodophyte were not (Supplementary Table 1; Supplementary Fig. 6). Using fluorescently labelled bacteria, which were observed intracellularly after 6 h of co-culture, we also confirmed that *P*. cf. *balticum* consumes prokaryotes (Fig. 2g; Supplementary Fig. 7). Despite the capability of *P*. cf. *balticum* to exploit both dissolved inorganic and particulate organic nutrients, it was not able to survive under pure heterotrophic conditions (in the dark with prokaryotic and/or eukaryotic prey) (Fig. 2h), demonstrating that phago-heterotrophy alone is not sufficient to sustain this dinoflagellate, and photosynthesis is requisite for survival. These results reveal that *P*. cf. *balticum* is an obligate phototroph, facultative phago-heterotroph, constitutive mixotroph^10^ that relies on acquiring carbon from photosynthesis, while supplementing its nutrition through particulate consumption (Supplementary Note 4).

### Mucospheres are used to capture marine microbes

**Fig. 3:**
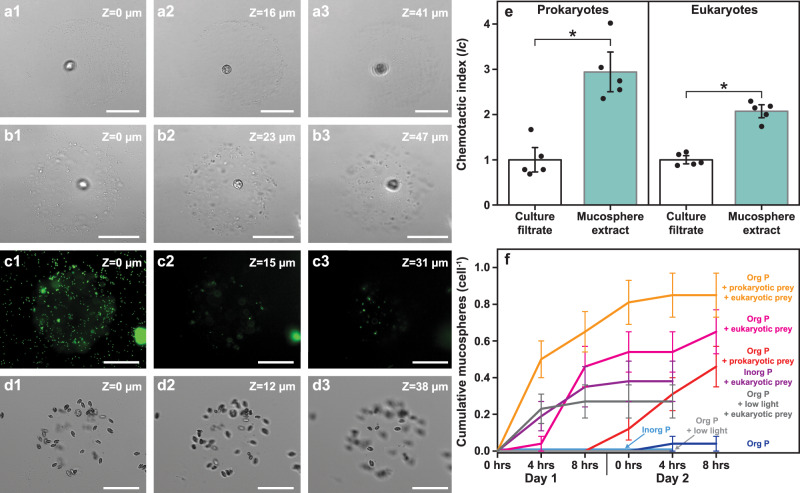
Detail of mucospheres constructed by *Prorocentrum* cf. *balticum* cells to aid prey capture. **a**–**d** Three-dimensional structure of the mucospheres with a Z-stack sequence from the (1) bottom, (2) middle and (3) top, with  a mucosphere constructed in the absence of prey (axenic culture) (**a**), a mucosphere with captured prokaryotes (**b**), the same mucosphere as in **b** visualised with the fluorescent stain SYBR green (**c**), and a mucosphere with captured eukaryotic prey (*R. salina*) (**d**). **e** Chemotactic response (as Chemotactic Index (*Ic*)) of prokaryotic prey (microbiome present in the xenic *P*. cf. *balticum* culture) and eukaryotic prey (*R. salina*) to mucosphere derived chemicals. Mean ± standard error (*n* = 5 biologically independent samples). Mucosphere-derived chemicals attracted significantly (*) more prokaryotic and eukaryotic prey than culture filtrate controls (two-sided t-test, *p* = 9.27e−04 for prokaryotic and *p* = 4.07e−05 for eukaryotic prey). **f** Cumulative mucosphere production in conditions with different resource availability observed at 0, 4, and 8 h after illumination (14:10 light:dark cycle) over two days. The calculated values include when a single *P.* cf. *balticum* cell produced multiple mucospheres. Mean ± standard error (*n* = 26 cells; Kruskal–Wallis test, *p* = 4.06e−12). Images **a**–**d** were taken with an Inverted Fluorescence Microscope (Nikon Eclipse Ti), fitted with a Nikon FITC 480/30 nm ex 535/45 nm em filter and a monochrome camera (Nikon DS-QiMc) under 200× magnification. Scale bars = 50 µm.

Phago-heterotrophy relies on effective prey capture and *P*. cf. *balticum* has developed a sophisticated behaviour to increase encounter rates. It involves the construction of a mucoid prey capture apparatus that we have termed a ‘mucosphere’, which is used to attract and immobilise a range of microbes from small prokaryotic to large eukaryotic prey. After ~8 h of photosynthesis (Supplementary Table 2), *P*. cf. *balticum* exudes mucus, spinning and shaping it into an intricate three-dimensional sphere ~100 µm in diameter (Supplementary Note 5; Supplementary Fig. 8a). Following construction of its mucosphere, the *P*. cf. *balticum* cell encapsulates itself within (Fig. 3a; Supplementary Movie 1). Prokaryotic (Fig. 3b, c) and eukaryotic cells (Fig. 3d) then become entangled in the outer adhesive surface (Supplementary Movie 1) and are immobilised but remain intact and alive, even after 24 h (Supplementary Fig. 9). Prey immobilisation in mucospheres is highly effective, allowing *P*. cf. *balticum* to trap numerous microbes simultaneously and restrain prey up to 6 times larger than itself (Supplementary Fig. 10; Supplementary Movie 1). During our observations, there were no occasions where live *P*. cf. *balticum* cells became entangled in another mucosphere, and cells could readily exit the internal area of their own mucosphere (Supplementary Fig. 8c) to either abandon it, or haul it vast distances through the surrounding medium (observed over the 15 mm width of a well in a 24 multi-well plate, which represents 10^3^ cell lengths), by remaining attached via the longitudinal flagellum (Supplementary Movie 1). During our laboratory observations, each *P*. cf. *balticum* cell constructed an average of one mucosphere per day (Supplementary Table 2) and consumed a single immobilised eukaryotic prey cell (e.g., *R. salina)*, after which the mucosphere was abandoned. This suggests mucosphere production is a prerequisite of phago-heterotophy for this species and that a single eukaryotic prey of sufficient size (e.g., ~12 µm in length for *R. salina*) is enough to satiate a *P*. cf. *balticum* cell, rendering continued habitation of the mucosphere unnecessary. Indeed, given that the mucospheres are negatively buoyant (Supplementary Fig. 11), continued association with a laden mucosphere could be disadvantageous for a phototrophic organism due to it sinking from the euphotic zone. This sophisticated foraging behaviour avoids the need for continuous active hunting, captures a variety of microbes, and allows subsequent prey selection, while restraining prey for easier peduncular consumption.

### Mucospheres attract microbial prey

To identify how the mucosphere can trap such large numbers of microbes, we tested whether these structures contained chemical cues that actively attract prey cells. We harvested mucospheres from an axenic *P*. cf. *balticum* culture, extracted and concentrated the associated chemicals, then tested the chemotactic response of potential prey cells^11,12^. Both eukaryotic and prokaryotic cells were significantly attracted to mucosphere chemical extracts (two-sided t-test, *p* < 0.01; Fig. 3e), exhibiting a two and three-fold enrichment over controls, respectively. This reveals that unlike spider webs, these mucospheres do not rely solely on passive prey interception but actively attract microbial prey. Other mucilage-based prey capture devices, termed ‘mucus traps’, have been reported to be produced by some mixotrophic dinoflagellate species. For example, *Alexandrium pseudogonyaulax* produces a mucus mass that is towed as it swims, capturing the microbes it encounters^13–15^ and three *Dinophysis* species release ‘cohesive mucilaginous clumps’ or ‘mucus threads’ that detach from their progenitors and capture prey passively^16–18^. While these mucilage-based prey capture devices also “trap” potential prey cells, none of them reach the level of sophistication of the mucosphere of *P*. cf. *balticum* which has a three-dimensional shape that is intricately crafted for hours, houses the generating cell, and actively chemoattracts prey. Given that multiple distantly related dinoflagellate species use a range of mucilaginous-based mechanisms to immobilise their prey, it strongly suggests that this foraging strategy has been largely overlooked and is prevalent in the global ocean.

### Prey, nutrients, and light affect mucosphere production

The production and shaping of a mucosphere is potentially energetically intensive. To understand this investment by *P*. cf. *balticum*, we tested mucosphere production under replete or limited resource conditions with 26 individual cells (*n* = 26) from one clonal strain. In the absence of its microbiome (i.e., under axenic culture conditions), the phototrophic growth of *P*. cf. *balticum* was inhibited when the organic phosphate source (sodium β-glycerophosphate) traditionally included in the growth medium used to culture dinoflagellates (K medium)^19^ was provided, but growth resumed when phosphorus (P) was provided as an inorganic form (sodium phosphate; Supplementary Figs. 12–15). Using this dependency, we conditioned an axenic *P*. cf. *balticum* culture to grow with organic phosphate for two weeks, exhausting internal stocks of P (P deplete) and creating a need for P acquisition via phago-heterotrophic consumption. We then monitored mucosphere production of individual cells at 0, 4 and 8 h after light exposure for two consecutive days (light cycle 14:10 h light:dark) (Fig. 3f; Supplementary Table 2). The presence of prey strongly influenced mucosphere production. No mucospheres were produced in the absence of prey in P replete or deplete conditions in the first 24 h. Among our 26 independent replicates, 12 and 46% of cells in P deplete conditions, constructed mucospheres in the presence of prokaryotic and axenic eukaryotic prey, respectively, with this increasing to 69% when both prey types were present. Under P replete conditions, 42% of *P*. cf. *balticum* cells made mucospheres in the presence of axenic eukaryotic prey (Supplementary Table 2). The mean daily number of mucospheres produced by *P*. cf. *balticum* under all conditions in the presence of prey was ~1, but some cells produced up to 2 mucospheres in 24 h (Supplementary Table 2). The time by which mucospheres were constructed varied from 6 to 28 h, with a swifter response in the presence of eukaryotic prey (6, 8, and 9 h versus 24 h for prokaryotic prey). The amount of time that *P*. cf. *balticum* cells spent within their mucosphere varied from <4 to >28 h, with a mean between 5.3 and 14.4 h depending on nutrient and prey conditions (Supplementary Table 2). These results strongly suggest that exometabolites derived from prey cells trigger mucosphere production, that mucosphere production is valuable even if dissolved nutrient levels are sufficient, and that *P*. cf. *balticum* can differentiate the chemical signatures of prokaryotic and eukaryotic prey.

Light exposure also affected mucosphere production, with only 23% of cells constructing mucospheres in the first 24 h under low light conditions compared with 46% in optimal light when axenic eukaryotic prey were present (Supplementary Table 2). When light levels were optimal, mucosphere production continued throughout the second day of monitoring (an additional 11% of cells produced mucospheres) but no additional mucospheres were constructed in low light conditions on day two (Fig. 3f). This likely reflects the initial allocation of residual photosynthetic energy to mucosphere production, then a transition to energy conservation when light becomes limiting. Staining the mucospheres with the acid polysaccharide stain Alcian Blue^20^ and the protein stain Coomassie Brilliant Blue^21^ (Supplementary Fig. 16a, b) revealed that their composition is analogous to transparent exopolymeric particles (TEP), a chemically diverse group of particulate material that consists mainly of carbon-rich acidic polysaccharides excreted by marine microbes^20^. This confirmation of the carbon-rich composition of mucospheres further supports our assessment that photosynthesis is a prerequisite for mucosphere production. We therefore conclude that mucospheres are produced by *P*. cf. *balticum* with carbon accumulated through photosynthesis and are used to capture prey that can be consumed to obtain other potentially limiting nutrients such as phosphorus, required for continued phototrophy and cell division.

### Mucospheres contribute significantly to the ocean carbon cycle

Mucosphere production has a potentially profound impact on ocean biogeochemical cycling. The chemoattractant properties of mucospheres likely increase prey capture, especially in the oligotrophic ocean where cells are sparsely distributed. This could increase rates of consumption, boosting trophic transfer of both nutrients and carbon. Furthermore, mucospheres are abandoned once the producing cells are satiated. The discarded mucospheres are negatively buoyant, becoming part of the POC pool and contributing to the vertical carbon flux either by being incorporated into aggregates, or sinking like other mucoid structures (Supplementary Fig. 11). For example, similar mucoid feeding structures produced by Appendicularians known as ‘houses’^22^ have been shown to contribute 12–83% of the total POC flux from the surface ocean, representing a significant proportion (between 0.60 and 1257 mg C m^−2^ d^−1^) of the marine carbon budget^23^. We propose that given the widespread distribution and abundance of *P*. cf. *balticum* in the ocean, abandoned mucospheres could contribute significantly to the vertical carbon flux. To estimate the magnitude of this export, we coupled measurements of mucosphere production and their carbon content in culture, with an assessment of the natural spatio-temporal distribution of *P*. cf. *balticum* (Fig. 1e; Supplementary Fig. 17 and 18). To calculate the mean carbon content per mucosphere, we measured the amount of organic carbon in the mucus (defined as >0.4 µm^20^) collected from a culture of axenic *P*. cf. *balticum* and approximated the number of mucospheres produced (using the cell abundance, growth rate and a measurement of the proportion of cells that produce mucospheres in 24 h grown under the same conditions). We then applied a method previously used to assess the carbon export contributions of Appendicularians^23^, to estimate the carbon export potential of mucosphere production. Specifically, we used the mean carbon content per *P*. cf. *balticum* mucosphere of 154.3 (± 19.5) pg, the lowest and highest average cell abundance of *P*. cf. *balticum*, 19 and 137 cells L^−1^, and the maximum cell number (3350 cells L^−1^) recorded across a decade of monthly microscopic cell observations from nine oceanographic time-series stations distributed around the Australian continent covering 30 degrees of latitude (Supplementary Fig. 18; Supplementary Data File 1). We then applied a daily mucosphere production rate of 23% measured under low light conditions (20 µmol m^−2^ s^−1^) in the presence of eukaryotic prey (Supplementary Table 2). While these observations were made under stable laboratory conditions, our data suggest that mucospheres are produced when prey is detected, and it is therefore conceivable that production and abandonment rates would be similar in nature. Applying these values, we estimate the contribution of carbon from mucospheres generated by *P*. cf. *balticum* to be 0.04–0.29 mg C m^−2^ d^−1^ but this value can be as high as 7.13 mg C m^−2^ d^−1^ when peak cell abundances are reached. If each mucosphere snares on average ~50 prokaryotic and 10 small (10 µm diameter) spherical eukaryotic cells (which according to our observations represents a conservative estimate, see “Methods”), the contribution increases to 0.17–1.24 mg C m^−2^ d^−1^ for the lowest and highest cell abundance respectively, and 30.30 mg C m^−2^ d^−1^ for the maximum recorded cell abundance. These values overlap with the range of the large carbon flux contribution previously characterised for some Appendicularian species (0.60–1, 257 mg C m^−2^ d^−1^)^23^ despite mucospheres being five orders of magnitude smaller than Appendicularian houses^23^. In addition, the contribution of mucospheres also overlaps with other carbon export mechanisms such as zooplankton faecal pellets (0–218 mg C m^−2^ d^−1^) and organic aggregates (0–110 mg C m^−2^ d^−1^)^24^ and is sizeable considering this is the contribution of a single species. To determine the significance of the mucosphere production contribution in terms of the biological carbon pump, we scaled these values across the global ocean (excluding the polar regions) for a 60 m euphotic zone and estimated between 5.57 × 10^−5^ and 4.02 × 10^−4^ Gt C is contributed daily, or 0.02–0.15 Gt C annually, representing 0.1–0.7% of the estimated total export from the euphotic zone (20 Gt)^3^. While microorganisms are typically considered to only contribute dead or senescent cells to the biological carbon pump, our results define a new mechanism for POC production and subsequent ocean carbon cycling.

### The diurnal cycle of *P*. cf. *balticum* impacts carbon fluxes

**Fig. 4:**
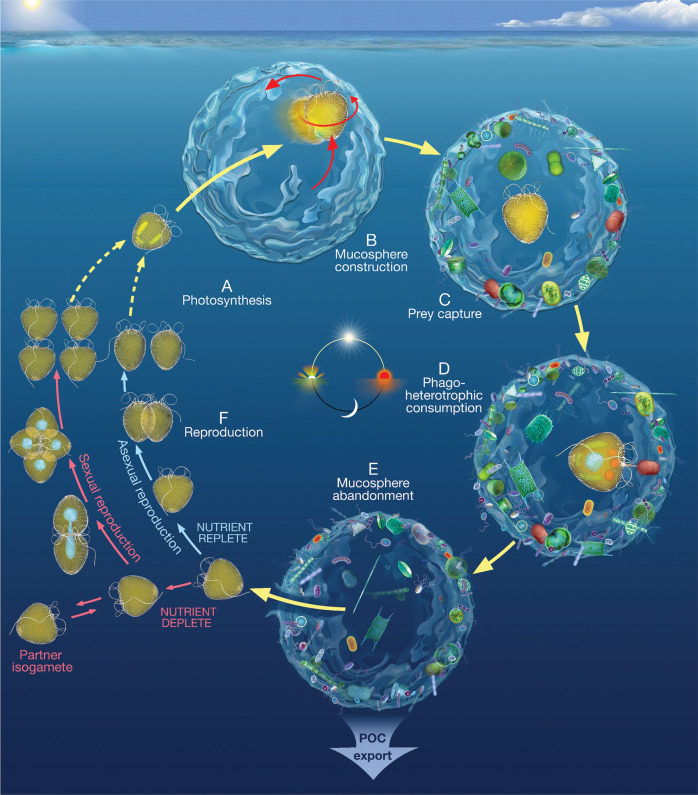
Summary of *Prorocentrum* cf. *balticum* behaviours. An infographic showing the daily cycles of **A** photosynthesis, **B** mucosphere production, **C** prokaryotic and eukaryotic prey attraction and capture, **D** peduncular phago-heterotrophic feeding, **E** mucosphere abandonment and carbon export potential, and **F** asexual or sexual reproduction. The solid yellow arrows represent the daily cycle of the *P*. cf. *balticum* cell including entering the mucosphere during construction in (**B**) and exiting the mucosphere in (**E**). The light blue arrows show the *P*. cf. *balticum* cell through the asexual reproduction stage and the pink arrows represent the sexual reproduction stage. The broken yellow arrows signify one of the succeeding *P*. cf. *balticum* cells from either of the reproduction stages re-entering the daily cycle.

Here we describe the metabolic and ecological characteristics of a marine protist that exhibits flexible nutrient acquisition and reproduction capabilities, and a sophisticated foraging strategy that can contribute to the marine carbon flux in a manner that is currently not accounted for in biogeochemical models. In summary (Fig. 4), the process begins with *P*. cf. *balticum* photosynthesising in the early stages of the light cycle (A) and converting fixed carbon into an apparatus that aids prey capture by exuding, then spinning and shaping mucus into a mucosphere (B). The fully formed mucosphere attracts and captures both prokaryotic and eukaryotic cells (C) that *P*. cf. *balticum* selectively consumes via peduncular myzocytosis (D). Once satiated (typically in the evening), *P*. cf. *balticum* abandons the mucosphere laden with surplus prey cells, which then contributes to the downward flux of POC from the surface ocean (E). The unburdened *P*. cf. *balticum* cell is then free to divide asexually in preparation for the next daily cycle, or under prolonged nutrient limitation, perform sexual reproduction (F). Our discovery that a mixotrophic protist uses mucospheres for prey capture uncovers a previously overlooked mechanism for marine primary producers to contribute to the vertical flux of carbon, and when the global abundance of *P*. cf. *balticum* is considered, the contribution from this behaviour is significant. This highlights the critical need to further describe and include protistan behaviour in Earth system models.

## Methods

### Isolation, identification, and distribution of *P*. cf. *balticum*

#### Isolation

A 20 µm plankton net was used to collect phytoplankton from the Port Hacking 100 m Australian Integrated Marine Observing System (IMOS) National Reference Station located on the continental shelf of southeast Australia (34.120°S, 151.224°E) in September 2018. The collected material was enclosed in a sealed jar, and stored in an incubator maintained at 20 °C under ~150 µmol m^−2^ s^−1^ light on a 14:10 h light:dark cycle. After four weeks of incubation with no added nutrients, it was assumed that the remaining dinoflagellate species were likely osmotrophic or mixotrophic, surviving through acquisition of organic nutrients or by means of ingestion. Single cells of *Prorocentrum* spp. were targeted and isolated using the micropipette technique^25^ and placed in individual wells of a 96 multi-well plate (Falcon, Corning, New York, USA) with 200 µL, 0.2 µm filter sterilised and autoclaved natural seawater collected from the sampling location and incubated under the same conditions. After several days, K medium (-Si)^19^ was gradually added as the cells began to grow. Once the isolated strains were growing consistently and a sufficient cell density was reached (approximately 1 × 10^2^ cells), they were transferred to 25 cm^2^ (50 mL) sterile vented polystyrene tissue culture flasks (Falcon, Corning, New York, USA). Established cultures were then maintained in these vessels, in K medium (-Si) made from sterile natural seawater collected from the isolation location, at a temperature of 20 °C, salinity of 35, under ~150 µmol m^−2^ s^−1^ light on a 14:10 light:dark cycle in a plant growth chamber (Fitoclima S600, Aralab, Rio de Mouro, Portugal), henceforth referred to as standard conditions.

#### Scanning Electron Microscope (SEM) morphological analysis

Two millilitres of living culture was gently filtered onto 1 cm diameter, 1 µm pore size, Nucleopore polycarbonate filters, rinsed with distilled water to remove salt crystals, and the filters gently air dried. Filters were mounted on aluminium stubs, sputter-coated with platinum, and analysed using an Emission SEM (Model Su-70, Hitachi, Tokyo, Japan).

#### Phylogenetic analysis

Cells from 100 mL of each established *P. rorocentrum* cf. *balticum* strain were harvested by centrifugation at 600 × *g* for 10 min and DNA extracted using a DNAeasy Powersoil Kit following the manufacturer’s instructions (Qiagen, Venlo, The Netherlands). The D1–D6 gene region of the 28S large subunit (LSU), the 18S small subunit (SSU), the internal transcribed spacer 1 (ITS1), and the complete SSU-ITS-LSU rDNA gene regions were amplified using the primers D1R-F (ACC CGC TGA ATT TAA GCA TA) and 28-1483R (GCT ACT ACC ACC AAG ATC TGC)^26,27^, Dino18SF1 (AAG GGT TGT GTT YAT TAG NTA CAR AAC) and 18ScomR1 (CAC CTA CGG AAA CCT TGT TAC GAC)^28^, ITSA (CCT CGT AAC AAG GHT CCG TAG GT) and ITSB (CAG ATG CTT AAR TTC AGC RGG)^29^ and Pro18SF (TGA TTA CGT CCC TGC CCT TT) and Pro 28SR (CAT CGC CAG TTC TGC TTA CC)^30^ respectively, and analysed separately. These genetic markers were selected because they are commonly used for the *Prorocentrum* genus, and many sequences are publicly available in GenBank (www.ncbi.nlm.nih.gov) for comparison. PCR amplifications were carried out in 25 µL reaction volumes containing Immomix mastermix (Bioline, Berlin, Germany) both forward and reverse primers (0.4 µM final concentration) and 1 µL of 10 ng µL^−1^ template in a 96 Well Thermal Cycler (Applied Biosystems, Waltham, Massachusetts, USA). Thermocycling conditions for the LSU region were 95 °C for 10 min, 30 cycles at 94 °C for 1 min, 45 °C for 1 min, 72 °C for 1 min with a final step at 72 °C for 7 min^31^; conditions for the SSU region were 95 °C for 10 min, 35 cycles at 94 °C for 30 s, 56 °C for 30 s, 72 °C for 45 s with a final step at 72 °C for 10 min^30^; conditions for the ITS1 region were 95 °C for 10 min, 35 cycles at 95 °C for 1 min, 55 °C for 45 s, 72 °C for 1 min 15 s with a final step at 72 °C for 7 min^32^ and the complete SSU-ITS-LSU region were 95 °C for 10 min, 35 cycles at 94 °C for 30 s, 56 °C for 30 s, 72 °C for 45 s with a final step at 72 °C for 10 min^30^. Amplification products (~ 950 bp) for each gene region were purified and sequenced in both directions using Sanger sequencing by a commercial service (Australian Genomic Research Facility (AGRF), Queensland, Australia) and sequences deposited in GenBank with accession numbers LSU MW024106-MW02409; SSU MW024110-MW024113; ITS1 MW024089-MW02492 and SSU-ITS-LSU MW024115-MW024118. Phylogenetic analyses were conducted in Geneious Prime v1.8.0 (Biomatters, Ltd, Auckland, New Zealand)^33^. Publicly available sequences of *Prorocentrum* spp., and other dinoflagellates used as out-groups were downloaded from GenBank (www.ncbi.nlm.nih.gov) and aligned with the sequences obtained from this study using the MUSCLE algorithm (maximum number of iterations 8)^34^. Sequences from the LSU, SSU, and ITS1 regions were truncated to 616, 1424, and 484 bp, respectively and analysed separately. Maximum Likelihood (ML) phylogenetic trees were generated for both regions with PHYML with 1000 bootstraps^35^ using a GTR substitution model and an estimated gamma distribution. Bayesian analysis was performed for both regions using MrBayes 3.2.6^36^ by means of the GTR + G (general-time reversible with gamma-shaped among-site variation) model. Bayesian analyses were carried out in four simultaneous runs with four chains each for 3.1 × 10^6^ generations, sampling every 1000 trees and 1000 trees were discarded as burn in.

#### Global distribution

The eukaryotic amplicon sequencing dataset from the *Tara* Oceans expedition was used to assess the distribution and relative abundance of *P*. cf. *balticum* in the global ocean. The total 18Sv9 rDNA information organised at the metabarcode level including *Tara* Ocean samples from all stations, depths, and size fractions, was downloaded from the Pangaea data portal (DOI:10.1594/PANGAEA.873275)^37^. Metadata for all *Tara* Oceans samples and stations, including filtered size class and location, was downloaded from the Pangaea data portal (DOI:10.1594/PANGAEA.875582)^38^. The primers used for sequencing the SSU gene region of *P*. cf. *balticum* (Dino18SF1 and 18ScomR1) described in the methods section titled ‘Phylogenetic analysis’, did not cover the area required for comparison with the eukaryotic amplicon sequences from the *Tara* Oceans dataset. Therefore, the 1433 bp SSU and the 1834 bp SSU-ITS-LSU consensus sequences from our Sanger sequencing were de novo assembled using the inbuilt Geneious assembler of Geneious Prime v1.8.0 (Biomatters, Ltd, Auckland, New Zealand). The sequences had a 25 bp overlap, creating a sequence now 3242 bp long spanning the required region. To confirm the sequences were assembled correctly, we then mapped this to a 7,632 bp long reference sequence of a closely related species (*P. minimum* strain D-127; accession no. JX402086).

The newly assembled 3242 bp sequence, now covering the required 129 bp region for comparison with the 18Sv9 region, allowed the identification of a unique barcode sequence with a 100% match over its complete length. Assessment of the specificity of the barcode for *P*. cf. *balticum* identified this sequence is also a match for some strains assigned as *P. minimum*, *P. dentatum* and *P. donghaiense* in Genbank suggesting it may not exclusively represent the abundance of *P*. cf. *balticum*. However, there were 8049 barcodes taxonomically designated as species within the *Prorocentrum* genus in the *Tara* Oceans metabarcode dataset, 82 of these as *P. minimum*, seven as *P. dentatum* and 2620 as *P. donghaiense*. This demonstrates the 18Sv9 region has the capability of discriminating many *Prorocentrum* strains and while the barcode that was a 100% match to our *P*. cf. *balticum* sequence may not be exclusive to this species, its use represents the most specific method available for estimating the distribution of *P*. cf. *balticum* in the global ocean to date.

The abundance of the *P*. cf. *balticum* barcode was extracted from the *Tara* Oceans metabarcode dataset for 99 *Tara* Oceans stations, which represents all stations except those located in polar regions that were not publicly available at the time of analysis. Relative abundance values were calculated by normalising the raw counts by the sum of all counts in a sample. Relative abundance per million counts was then calculated by multiplying the relative abundance values with a scaling factor of 1,000,000.

*Prorocentrum* cf. *balticum* cells have a transdiameter of ~15 µm, though there is some phenotypic variation (Supplementary Fig. 14), and when encapsulated within a mucosphere, the total diameter can increase to 100 µm. Therefore, the relative abundance of the *P*. cf. *balticum* barcode, was investigated for the three size classes that covered this range (<5, 5–20, and 20–180 µm), for all depths (Supplementary Fig. 3a–c). Intuitively, the abundance was highest in the 5–20 µm size class and the surface samples (~5 m) generally represented the abundance within the euphotic zone (~60 m), thus this size fraction and depth was used to represent the relative abundance of *P*. cf. *balticum* in the global ocean on a world map at the latitude and longitude from each station using QGIS v2.18.16^39^ (Fig. 1e).

#### Toxin analysis

To check for the presence of okadaic acid, dinophysistoxin-1, dinophysistoxin-2, and tetrodotoxin which are neurotoxins that negatively affect human health and are produced by other species of *Prorocentrum*, two *P*. cf. *balticum* strains (UTSPH2D1 and UTSPH3D3) were grown in K medium (-Si), at 20 °C, at a salinity of 35, under ~150 µmol m^−2^ s^−1^ light on a 14:10 light:dark cycle in 1 L glass conical flasks. Once the cultures reached late exponential growth phase, the cells were harvested by centrifugation (600 × *g* for 10 min) resulting in a pellet from each strain of approximately 5 × 10^7^ cells. The cell pellets were then frozen at −80 °C, freeze dried, and sent for analysis through a commercial service at the Cawthron Institute, New Zealand, using an in-house LC-MS/MS method^40,41^.

### Microscopy

Microscopy was the primary method of observation in this study. A description of the microscopes used is provided below, with the application of each type of microscopy described in the relevant experimental sections.

#### Light microscopy

An inverted light microscope (Nikon Eclipse TS100, Japan) was used to visualise live or preserved cells under 200, 400, or 1000× magnification. Images or movies were captured using a colour camera (Infinity 1, Lumenera, Ontario, Canada) using proprietary software (Infinity Analyse v6.4.0).

#### Fluorescence microscopy

An inverted fluorescence microscope (Nikon Eclipse Ti, Japan) fitted with FITC 480/30 nm ex 535/45 nm em, Texas Red 560/40 nm ex 630/60 nm em, and DAPI 375/28 nm ex 460/60 nm em filters was used to visualise live and preserved cells in multi-well plates under 200, or 400× magnification and were imaged with a monochrome camera (Nikon DS-QiMc) using proprietary software (NIS Elements v4.60).

An upright fluorescence microscope (Nikon Eclipse Ni, Japan) fitted with a Cy5 620/60 nm ex 700/75 nm em filter and a monochrome camera (Nikon DS-Qi2) with proprietary software (Infinity Analyse v6.4.0) under 200, 400, or 1000× magnification was used for more detailed observation of cells and for obtaining cell size measurements.

#### Confocal microscopy

Confocal microscopy was used to visualise the cells in the most detail. An inverted confocal microscope (Nikon AR1, Japan) using lasers 405.6 and 561.0 nm ex and the following filters TRITC 595 nm em, Alex610 700 nm em TD was used to image cells with a digital camera (Nikon A1Plus) under 1000× magnification with oil immersion using proprietary software (NIS Elements v4.60).

### *P*. cf. *balticum* phototrophic growth experiments

#### Electron transport rate measurements

To examine the photosynthetic activity of *P*. cf. *balticum*, we determined its capacity to utilise light using a steady-state light curve protocol in a Pulse Amplitude Modulated Fluorometer (Water-PAM, Walz, Germany) (Supplementary Fig. 4a)^42^. A 2 mL aliquot of an exponentially growing *P*. cf. *balticum* culture (strain UTS3D3) grown under standard conditions was placed in a cylindrical quartz cuvette and dark-adapted for 15 min. Chlorophyll-a fluorescence emission was then measured when cells were exposed to saturating pulses of red light (650 nm) every 30 s for 4 min at increasing light intensities (0, 19, 30, 47, 73, 113, 168, 258, 387, 550, 766, 1257 and 1890 µmol m^−2^ s^−1^). The mean of the final three measurements at each light intensity was used to estimate the effective quantum yield (Φ_PSII_), and the relative Electron Transport Rate (rETR) was calculated using the following equations:

1 $${\varPhi }_{{{{{{\rm{PSII}}}}}}}=({F}_{M}{\prime} -F{\prime} )/{F}_{M}{\prime}$$

where, *F’* and *F*_*M*_*’* is the minimum and maximum fluorescence at each light intensity respectively, and,

2 $${{{{{\rm{rETR}}}}}}={{{{{\rm{PAR}}}}}}* {\varPhi }_{{{{{{\rm{PSII}}}}}}}* 0.5* 0.85$$

where, PAR is photosynthetically active radiation.

#### Phototrophic growth and strain comparison

To understand how this photosynthetic capacity translated to phototrophic growth and to compare the four isolated strains of *P*. cf. *balticum*, growth was measured under standard conditions. Cells were inoculated into triplicate 25 cm^2^ (50 mL) sterile vented polystyrene tissue culture flasks (Falcon, Corning, New York, USA) at a concentration 1 × 10^3^ cells mL^−1^. A 1 mL aliquot was removed every 1–3 days and preserved with paraformaldehyde (1% v/v final concentration) (Emgrid Australia, Pooraka, South Australia, Australia) and the cell abundance quantified using a flow cytometer (Beckman Coulter Cytoflex LX, Indianapolis, USA) with blue laser (488 nm) excitation and a combination of 690/50 and 585/42 nm detection with the CytExpert v2.4 software. Rainbow fluorescent QC beads (Cytoflex Daily QC Fluorospheres, BD Sciences) were also used to ensure the accuracy of the settings and gating strategy between sampling days (Supplementary Fig. 21). The maximum growth rate was calculated using the slope of natural logarithm transformed cell abundance in the linear portion of the 16–day growth curve. Phototrophic growth of the four isolated *P*. cf. *balticum* strains were similar so strain UTSPH3D3 was selected for all future experiments (Supplementary Fig. 4b) and has been deposited at the Australian National Algal Culture Collection (ANACC) as strain CS-1390.

### Preparation and verification of axenic *P*. cf. *balticum* cultures

#### Preparation

Bacteria are closely associated with protists, forming a distinct microbiome. Mixotrophic protists can often ingest bacteria as well as other unicellular eukaryotes^43–45^, therefore it was necessary to remove the microbiome from *P*. cf. *balticum* to test its true phototrophic and mixotrophic growth. An axenic *P.* cf. *balticum* culture strain (UTSPH3D3) was produced following the methods described in Shishlyannikov et al.^46^ with some modifications. Approximately 50 mL of the exponentially growing xenic culture was gravity filtered through a 47 mm polycarbonate filter membrane (pore size 5 µm) (GE life sciences, Trevose, PA, USA), ensuring 20 mL of liquid remained. The remaining liquid was swirled to re-suspend the cells then rinsed with 50 mL sterile natural seawater four times before re-suspending the cells in the remaining 20 mL and transferring the suspension and filter membrane to a sterile 50 mL centrifuge tube (Thermo Fisher Scientific, Hampton, New Hampshire, USA). Triton-X 100 (Sigma Aldrich, St. Louis, Missouri, USA) was slowly added to the re-suspended cells at a final concentration of 20 µg mL^−1^ and gently inverted for 1 min. The cells were then filtered through another polycarbonate filter membrane, slowly rinsed, and continuously re-suspended by swirling with five portions of 100 mL K medium (-Si) to remove traces of Triton-X 100. The cells were swirled in the last 20 mL of K medium (-Si) and decanted into another 50 mL tube, along with the used polycarbonate filter membrane. An additional 30 mL K medium (-Si) was added to the tube for a final volume of 50 mL and the following antibiotics added: ampicillin (Astral Scientific, NSW, Australia) final concentration 50 µg mL^−1^, and ciprofloxacin (Astral Scientific, NSW, Australia) final concentration 5 µg mL^−1^. The cells were incubated with the antibiotics for 20 h at 20 °C in the dark, before being diluted 1:1 with K medium (-Si) and grown at 1 mL volume in 24 multi-well polystyrene plates (Falcon, Corning, New York, USA) under standard conditions. After one week in the 24 multi-well plate, the contents of each well was further diluted in 9 mL sterile seawater supplemented with K medium (-Si) and transferred to 50 mL flat tissue culture flasks.

#### Verification

After an additional week, the resultant cultures were screened for bacterial contamination by inoculating 1 mL of antibiotic treated culture into 5 mL of Difco Marine Broth 2216 (ROWE Scientific, new South Wales, Australia) and allowing 3 days growth at room temperature (at 160 rpm), after which tubes were visually inspected and turbid samples discarded. A second verification step included plating the cultures on agar made with Difco Marine Broth 2216, incubating at room temperature for 3 days and visualising bacterial colonies. The bacterial consortium was not removed entirely after this first treatment with antibiotics, so *P*. cf. *balticum* cultures were treated again using the same protocol but with additional exposure to cefotaxime, carbenicillin, gentamycin, and streptomycin (Astral Scientific, NSW, Australia) at final concentrations of 200, 100, 67 and 50 µg mL^−1^, respectively, and screened using liquid Difco Marine Broth 2216 (ROWE Scientific, new South Wales, Australia) and agar (ROWE Scientific, new South Wales, Australia) incubations. Treatments without visual turbidity in Marine Broth or colonies on agar were preserved with paraformaldehyde (1% v/v final concentration), stained with the nucleic acid stain SYBR Green I 10,000X (5:100,000 dilution)^47^ (Invitrogen, Carlsbad, California, USA) and any remaining bacterial cells visualised with fluorescence microscopy with FITC 480/30 nm ex 535/45 nm em and Texas Red 560/40 nm ex 630/60 nm em filters, and flow cytometry (Beckman Coulter Cytoflex LX, Indianapolis, USA) using blue laser (488 nm) excitation, SYBR 525/40 nm, Violet SSC detection and the CytExpert v2.4 software (Supplementary Fig. 12). Growth of the axenic *P*. cf. *balticum* culture was significantly inhibited and experimentation revealed the cells were unable to grow in standard K medium (-Si), but growth was restored when the organic form of phosphate (sodium β-glycerophosphate) was substituted with an inorganic form (sodium phosphate) at the same concentration (Supplementary Fig. 15). Therefore, both the xenic and axenic cultures were subsequently switched to K medium (-Si) with inorganic phosphate for ongoing maintenance and experimentation.

#### Confirmation that antibiotic treatment did not affect *P*. cf. *balticum* physiology

To test if the protocol used to remove the bacterial microbiome from the *P*. cf. *balticum* culture could have affected the health of the dinoflagellate cells themselves, the microbiome from the xenic culture was reintroduced into the axenic culture to develop an axenic+ culture and the phototrophic growth and cell dimensions compared. The bacterial microbiome was separated from the xenic *P*. cf. *balticum* cells by filtration through a 47 mm polycarbonate filter membrane (pore size 5 µm) (GE life sciences, Trevose, PA, USA) in a sterile filtration tower. A 1 mL aliquot of the homogenised mixture was added to five replicates of the axenic *P*. cf. *balticum* culture to produce five axenic+ replicate cultures. The xenic and axenic *P*. cf. *balticum* cultures were also split into five replicates each so that there were replicates of each of the xenic, axenic, and axenic+ cultures. These were then grown under standard conditions and transferred to fresh media every three weeks (approximate period of a growth cycle), for three months to allow the microbiome to stabilise. Growth rates were measured by inoculating 25 cm^2^ (50 mL) sterile vented polystyrene tissue culture flasks (Falcon, Corning, New York, USA) at a concentration of 1 × 10^3^ cells mL^−1^, incubating under standard conditions, and removing a 1 mL aliquot every 2 days, preserving with paraformaldehyde (1% v/v final concentration) and determining the dinoflagellate cell abundance using a flow cytometer (Beckman Coulter Cytoflex LX, Indianapolis, USA) as described in the methods section titled ‘Phototrophic growth and strain comparison’. Bacterial cell abundance was determined using blue excitation with 525/40 nm and Violet SSC detection after first staining with SYBR Green I 10,000X (Invitrogen, Carlsbad, California, USA) (5:100,000 dilution) and incubating for 10 min (Supplementary Fig. 14a). The maximum growth rate was calculated using the slope of natural logarithm transformed cell abundance in the linear portion of the 16-day growth curve.

Changes in dinoflagellate cell size and shape were also determined by measuring the length and width of 30 individual cells from each of the xenic, axenic and axenic+ cultures using the proprietary image analysis software on the upright fluorescence microscope (Supplementary Fig. 14b, c). To confirm the observations that *P*. cf. *balticum* cannot grow when provided an organic phosphate source in the absence of its microbiome, the growth experiment was repeated with treatments where either an inorganic or organic phosphate source was supplied and both dinoflagellate and bacterial cell abundances were monitored. This experiment showed the growth rate of the axenic *P*. cf. *balticum* culture was significantly reduced when grown with organic phosphate (Supplementary Fig. 14).

#### Characterisation of associated microbiome

To determine how the microbiome in the xenic and axenic+ cultures differed in both the organic and inorganic phosphate treatments, 30 mL from each replicate was filtered onto a 47 mm polycarbonate filter membrane (pore size 0.2 µm) (GE life sciences, Trevose, PA, USA) during the exponential growth phase, snap frozen in liquid nitrogen and stored at −80 °C until the DNA was extracted using a DNAeasy PowerWater Kit following the manufacturer’s instructions (Qiagen, Venlo, The Netherlands). DNA was then PCR amplified and sequenced by a commercial service (Australian Genome Research Facility (AGRF), Queensland, Australia) using Illumina adapter barcoded universal bacterial 16S rDNA gene (V1–V3 region) primers: 27F (AGA GTT TGA TCM TGG CTC AG)^48^, and 519 R (GWA TTA CCG CGG CKG CTG)^49^. Amplicons were then pooled and sequenced on the Illumina Miseq platform resulting in 300 bp paired end reads, using standard AGRF procedures.

The open source programme *R* version 3.6.3^50^ was used for amplicon read processing, statistical analysis, and production of microbiome figures^51^, primarily the *R*-packages *dada2* (version 1.14.0) and *phyloseq* (version 1.30.0). The raw fastq files were run through the *dada2* pipeline where they were quality filtered, dereplicated, and merged^52^ (*R* code: https://github.com/ARBramucci/Larsson2020). The merged reads were collapsed using the collapse no mismatch step, chimeras were removed using the removeBimeraDenovo function, and amplicon sequence variants (ASVs) were exported^53^. ASVs were annotated at 50% probability cut-off using the SILVA database (version 138)^54^. The annotated ASV table was secondarily filtered to remove any ASVs identified within DNA extraction and PCR blanks, as well as any ASVs not annotated to Kingdom Bacteria and all ASVs annotated as either chloroplasts or mitochondria. Reads were rarefied to  the even sampling depth of 23,960 reads (95% of the lowest total reads per sample) using the *phyloseq* rarefy to even depth function before further processing^55^, resulting in the removal of one under sequenced replicate. In order to test the effect of algal host and phosphate treatment on bacterial composition and confirm that the bacterial consortia of the various treatments did not differ significantly at the level of ASVs, we built Bray–Curtis distance matrices using 999 permutations and performed permutational multivariate analysis of variance (PERMANOVA) using the *adonis* function from the *vegan* package (version 2.5-7 in *R*^56^. The *R* function *pairwise.adonis* was used to perform multiple pairwise comparisons and the Benjamin-Hochberg correction was used to account for the false discovery rate^57^. A bar chart of rarefied abundances of microbiome ASVs making up >0.02% of a given sample was plotted using *ggplot2* and the NMDS of all rarefied ASVs was plotted using *phyloseq* (jaccard ordination, stress=1.33, stat ellipses (solid line= type t, 95% confidence; dashed line= type norm, 95% confidence) in *R*. The bacterial consortia of the xenic and axenic+ cultures overlapped in all the assigned genera present at over 0.02% relative abundance per sample (Supplementary Fig. 13b). The bacterial consortia were not significantly different regardless of the algal host (xenic or axenic+) or phosphate type (organic vs inorganic phosphate), indicating that physiological differences of *P*. cf. *balticum* cultures could not be ascribed to changes in their microbiome.

### Phago-heterotrophic feeding experiments

#### Eukaryotic prey

Eukaryotes used as prey were obtained from a variety of sources including the Australian National Algal Culture Collection (ANACC), the Bigelow National Centre for Marine Algae and Microbiota (NCMA), and the author’s personal collection (MEL). Details of these xenic cultures and the maintenance conditions are provided in Table 1. Cell size dimensions were estimated by imaging each microalgal culture using an inverted fluorescence microscope (Nikon Eclipse Ti, Japan) fitted with a monochrome camera (Nikon DS-QiMc) and measuring the diameter of individual cells using the proprietary image analysis software that had been size calibrated.

**Table 1:** Details of the unicellular eukaryotic prey species used in this study and their maintenance conditions.

Prey species	Class	Strain number	Origin	Temp. (°C)	Medium
*Amphidinium massartii*	Dinophyceae	CS-259	ANACC	25	*f*/2-Si
*Coolia palmyrensis*	Dinophyceae	UTSR7	MEL	25	Modified K
*Dunaliella tertiolecta*	Chlorophyceae	CS-14	ANACC	23	*f*/2
*Gambierdiscus lapillus*	Dinophyceae	UTSHI6B5	MEL	25	Modified K
*Gymnodinium catenatum*	Dinophyceae	CS-302/11	ANACC	25	GSe
*Nanochloropsis oceanica*	Eustmatophyceae	CS-179	ANACC	23	*f*/2
*Porphyridium purpureum*	Rhodophyceae	CS-25	ANACC	20	*f*/2
*Prorocentrum lima*	Dinophyceae	UTSHI1C5	MEL	25	Modified K
*Prymnesium parvum*	Prymnesiophyceae	CS-376	ANACC	20	*f*/2
*Rhodomonas salina*	Cryptophyceae	CS-24	ANACC	20	K-Si
*Proteomonas sulcata*	Cryptophyceae	CS-412	ANACC	25	GSe
*Scrippsiella* sp.	Dinophyceae	UTSTFB1	MEL	20	K-Si
*Tetraselmis* sp.	Chlorophyceae	CS-352	ANACC	23	*f*/2
*Thalassiosira weissflogii*	Bacillariophyceae	CCMP1050	Bigelow	20	*f*/2
*Thalassiosira pseudonana*	Bacillariophyceae	CCMP1010	Bigelow	20	*f*/2
*Thalassiosira rotula*	Bacillariophyceae	CCMP3264	Bigelow	20	*f*/2
*Tisochrysis lutea*	Prymnesiophyceae	CS-177	ANACC	23	*f*/2

References for media recipes: *f*/2^72^; Modified K^73^; K^19^.

In order to separate the effects of phago-heterotrophic feeding on eukaryotic and prokaryotic prey, axenic cultures of the cryptophytes *Rhodomonas salina* (CS-24) and *Proteomonas sulcata* (CS-412) were produced using the method described in the methods section titled ‘Preparation and verification of axenic *P*. cf. *balticum* cultures’, with cells being treated with a single dose of the antibiotics cefotaxime, carbenicillin, gentamycin and streptomycin (Astral Scientific, NSW, Australia) at final concentrations of 200 µg mL^−1^, 100 µg mL^−1^, 67 µg mL^−1^ and 50 µg mL^−1^, respectively.

To confirm phago-heterotrophic consumption of eukaryotic prey, the xenic *P*. cf. *balticum* culture was co-cultured with the phycoerythrin pigment-containing xenic *R. salina* culture in a 24-multiwell glass bottom plate (Cellvis, California, USA) for 48 h in standard culture conditions. The cells were observed live to confirm the peduncular feeding mechanism and the resulting green-fluorescent food vacuoles were observed initially with an inverted fluorescence microscope. Subsequent imaging was done using an inverted confocal microscope after fixation with paraformaldehyde (1% v/v final concentration) (Fig. 2f).

To test eukaryotic prey consumption more broadly, the xenic *P*. cf. *balticum* culture was inoculated into K medium (-Si) at a concentration of 5 × 10^2^ cells mL^−1^ in 24-multiwell glass bottom plates (Cellvis, California, USA) and co-cultured with the microalgal prey species listed in Table 1 for 48 h under standard culture conditions. Microalgal prey species <15 µm were inoculated at 1 × 10^3^ cells mL^−1^ and those that were >15 µm at 1 × 10^2^ cells mL^−1^. After 48 h of co-culture, the cells were observed live for entrapment of prey in mucospheres using fluorescence microscopy (Supplementary Table 1 and Supplementary Fig. 10). The unique peripheral location of the chloroplasts in *P*. cf. *balticum* (Supplementary Fig. 6 Control) allowed visualisation of red-fluorescent food vacuoles, defined as spherical red bodies within the cells interior usually devoid of chloroplasts, resulting from consumption of chlorophyll-containing prey items. Therefore, after the co-culture incubation, the cells were preserved with paraformaldehyde (1% v/v final concentration) and 30 *P*. cf. *balticum* cells from each treatment were inspected for the presence of the red-fluorescent food vacuoles resulting from phago-heterotrophic consumption using fluorescence microscopy (Supplementary Fig. 6).

#### Prokaryotic prey

The capacity for *P*. cf. *balticum* to consume prokaryotic prey was also tested. Fluorescently labelled bacteria (FLB) were prepared as per^58^. Briefly, 200 mL of the xenic *P*. cf. *balticum* culture was filtered through a 5 µm pore size 47 mm polycarbonate filter membrane to separate the *P*. cf. *balticum* cells from the natural bacterial microbiome. The filtrate containing only the natural bacterial microbiome was then filtered onto a 0.2 µm pore size 47 mm polycarbonate filter membrane and resuspended in 10 mL of sterile natural seawater and 2 mg of the green fluorescent stain 5-([4,6-dichlorotriazin-2-yl]amino)fluorescein hydrochloride (DTAF) (Sigma Aldrich, St. Louis, Missouri, USA) added. This suspension was then incubated at 60 °C for 2 h, filtered onto a 0.2 µm pore size 47 mm polycarbonate filter membrane, and rinsed with 100 mL sterile natural seawater three times. The stained bacterial microbiome was then resuspended a final time in 10 mL sterile natural seawater, aliquoted into 1.5 mL microtubes, and stored at −80 °C until use. Cells of the axenic *P*. cf. *balticum* culture were inoculated into 3 mL K medium (-Si with inorganic phosphate substituted) at 2 × 10^3^ cells mL^−1^ in 6 multi-well polystyrene plates (Falcon, Corning, New York, USA) and incubated for 24 h to allow the cells to produce mucospheres. The FLB were then added at a ratio equivalent to that of the xenic culture (5 × 10^6^ cells mL^−1^) and the cells were incubated together under standard culture conditions in the light. After 6 h, the cells were filtered onto a 5 µm pore size 25 mm black polycarbonate membrane (Steritech, Wetherill Park, NSW, Australia), washed five times with 10 mL sterile natural seawater and mounted on a glass microscope slide, with 1 drop of fluorescence grade immersion oil protected by a glass coverslip. Intracellular inclusions of FLB were then observed with a fluorescence microscope (Fig. 2g and Supplementary Fig. 7).

#### Obligate phototrophy of *P*. cf. *balticum*

To identify if *P*. cf. *balticum* is an obligate or facultative phototroph, growth of the axenic culture was measured under standard conditions with either 150 µmol light or in the dark. Cells in the dark had access to either prokaryotic prey, or eukaryotic prey. It was first confirmed that *P*. cf. *balticum* consumed *Proteomonas sulcata* and was a suitable eukaryotic prey to be used in this experiment by growing the two species in co-culture, then visualising food vacuoles using fluorescence microscopy as described in the methods section titled ‘Eukaryotic prey’. Cells were inoculated into six replicate wells of a 12 multi-well plate (Falcon, Corning, New York, USA) in 3 mL of K medium (-Si with inorganic phosphate substituted) at a concentration of 2 × 10^3^ cells mL^−1^. A 250 µL aliquot was removed every 3 days and *P*. cf. *balticum* cell abundance quantified on a flow cytometer (Beckman Coulter Cytoflex LX, Indianapolis, USA) using CytExpert v2.4 software with blue laser (488 nm) excitation and a combination of 690/50 nm and 585/42 nm detection (Fig. 2H). Rainbow fluorescent QC beads (Cytoflex Daily QC Fluorospheres, BD Sciences) were also used to ensure the accuracy of the settings and gating strategy between sampling days.

#### Sexual reproduction

The unusual form of sexual reproduction was observed when *P*. cf. *balticum* cells from the axenic culture were incubated in sterile natural seawater with no added nutrients in a 24-multiwell glass bottom plate for 2 weeks under standard culture conditions. The cells were first observed and imaged live using an inverted fluorescence microscope. The cells were then preserved with paraformaldehyde (1% v/v final concentration) and the nucleus visualised by staining with the nucleic acid dye 4′,6-diamidino-2-phenylindole (DAPI; Sigma Aldrich, St. Louis, Missouri, USA) at a final concentration of 1 µmol for 10 min (Fig. 2a–d and Supplementary Fig. 5). Sexual reproduction observed in our clonal cultures reveal homothallism.

### Mucosphere production and composition

#### Prey capture in mucospheres

Eukaryotic cells entrapped in the mucospheres were easily observed using standard light microscopy (Fig. 3d and Supplementary Fig. 10). Prokaryotic cells entrapped in mucospheres were observed by first staining with SYBR Green I 10,000X (5:100,000 dilution) and then visualised using a fluorescence microscope (Fig. 3c and Supplementary Figs. 8, 9). To test if the ensnared prey cells remained alive after being immobilised in the mucosphere for 24 h, the eukaryotic prey cells were stimulated by successive flashes of green-fluorescent light, which instantly induced a reaction to attempt to struggle free of the mucosphere (Supplementary Fig. 9a1–a4). The same technique was used to test the ease of escape of *P*. cf. *balticum* from within a mucosphere. *P. rorocentrum* cf. *balticum* cells could push through the mucosphere and escape within seconds (Supplementary Fig. 8c). To test if prokaryotic prey were alive after 24 h immobilised within the mucosphere, cells were dual stained with the nucleic acid stain SYBR Green I (Invitrogen, Carlsbad, California, USA) and the live/dead stain Propidium Iodide (PI) (Thermo Fisher Scientific, Hampton, New Hampshire, USA) (final concentration 30 µM). Both live and dead cells would emit a green-fluorescent signal from the SYBR Green I stain, but dead cells would also emit an orange-fluorescent signal from the PI penetrating intracellularly. (Supplementary Fig. 9b). Green and orange fluorescence were evaluated separately using the FITC 480/30 nm ex 535/45 nm em and Texas Red 560/40 nm ex 630/60 nm filters, respectively, then the images overlayed for visual assessment of whether the prokaryotic cells were alive or dead. This technique verified that immobilised prokaryotic and eukaryotic cells were alive.

#### Mucosphere production experiments

To understand the conditions which initiate mucosphere production, experimental treatments that manipulate the phosphorus source and light intensity were tested. A subsample of the axenic *P*. cf. *balticum* culture was diluted sufficiently so that 200 µL of medium would contain only one or two cells, this was then aliquoted into wells of a 96-multiwell plate. Cell behaviour was monitored at intervals of 0, 4, and 8 h after illumination (under standard conditions) for two days in the following treatments:

- K medium (-Si) with organic phosphate;
- K medium (-Si) with organic phosphate and prokaryotic prey;
- K medium (-Si) with organic phosphate and axenic eukaryotic prey;
- K medium (-Si) with organic phosphate and prokaryotic and axenic eukaryotic prey;
- K medium (-Si) with inorganic phosphate;
- K medium (-Si) with inorganic phosphate and axenic eukaryotic prey;
- K medium (-Si) with organic phosphate and low light;
- K medium (-Si) with organic phosphate with axenic eukaryotic prey in low light;

where the prokaryotic prey was ~1 × 10^3^ bacteria cells collected from the natural microbiome of the xenic *P*. cf. *balticum* culture, the eukaryotic prey was 1 × 10^2^ cells of the axenic culture of the cryptophyte *R. salina* and low light was 20 µmol m^−2^ s^−1^ (compared with standard conditions at 150 µmol m^−2^ s^−1^). The number of mucospheres was recorded for each well at each time point except for the low light and inorganic phosphate conditions which were not monitored for the final 8 h time point (due to low mucosphere production). Mucospheres sink to the bottom of the well and do not move which made it possible to track whether it was newly produced, was a repeat observation of a previously recorded mucosphere with either the *P*. cf. *balticum* cell remaining within, or whether it had been abandoned. A total of 26 individual wells were monitored for each condition. The results were corrected for cell division after the first 24 h and the proportion of cells which had produced a mucosphere calculated. Statistical significance at the 24 h timepoint was tested using a Kruskal-Wallis H test, followed by Mann–Whitney pairwise comparisons (with Bonferroni correction), and the results are presented in Supplementary Table 3.

#### Mucosphere composition analysis

Common microscopy stains were used to elucidate the composition of mucospheres. Cells from the axenic *P*. cf. *balticum* culture growing exponentially under standard conditions were inoculated into a 24-multiwell glass bottom plate in 2 mL volumes and left to produce mucospheres for 24 h. The non-fluorescent stains Alcian Blue and Coomassie Brilliant Blue are commonly used to identify polysaccharide-rich TEP and protein-rich TEP, respectively, so were used in this study to determine if mucospheres have a composition similar to either of these particle types commonly associated with marine systems. The stains were added to the wells (Alcian blue final concentration 0.02% of 8GX as per Passow and Alldredge^59^; Coomassie Brilliant Blue 0.04% final concentration as per Long and Azam^21^), incubated for 12 h, and then imaged on an inverted light microscope (Supplementary Fig. 16a, b). The fluorescent stains SYPRO red (Thermo Fisher Scientific, Waltham, MA, USA)^60^, Calcofluor White (Sigma Aldrich, St. Louis, Missouri, USA)^61^, BODIPY 505/515 (Thermo Fisher, Waltham, MA, USA)^62^, and Acridine Orange (Thermo Fisher, Waltham, MA, USA)^63^ were also added to different wells to test for proteins, β-polysaccharides, neutral lipids and mucopolysaccharides respectively, and were visualised using fluorescence microscopy. All fluorescent stains positively stained the *P*. cf. *balticum* cells themselves but not the mucospheres they produced (Supplementary Fig. 16c–f).

#### Mucosphere buoyancy experiment

To test the buoyancy of mucospheres, 50 µL (approximately 1000 cells) of the xenic *P*. cf. *balticum* culture growing exponentially under standard conditions was inoculated into 3 mL (which corresponds to the capacity of a single well) fresh K medium in a 24-multiwell glass bottom plate and 50 µL of the axenic *R. salina* culture added as prey. The plate was placed in INCell High Content Analyser 2200 (GE Healthcare, Kent, United Kingdom) and programed to image 50 fields of view in a single well, focused on the *Z*-plane on the glass base, under brightfield using the 10× objective every hour for 25 h. The images were then scanned for instances were mucospheres laden with prey had been produced and had settled to the bottom of the well indicating negative buoyancy (Supplementary Fig. 11).

#### Mucosphere as a chemoattractant

Previous studies have speculated about the role of chemicals in facilitating prey capture in mucilage-based structures produced by mixotrophic dinoflagellates^13,18^ but none have tested this explicitly. Here we measured the chemotactic response of an axenic *R. salina* culture and *P*. cf. *balticum*-associated bacteria towards mucosphere-derived chemicals, using the ISCA (In Situ Chemotaxis Assay)^11^. The ISCA is a microfluidic device composed of an array of wells (110 µl each) which can be filled with different chemicals. Here, we used three treatments: (i) ultra-filtered spent medium as a negative control, (ii) mucosphere extract (0.1 mg ml^−1^) and (iii) 10% Difco Marine Broth 2216 (ROWE Scientific, New South Wales, Australia) as a positive control.

The mucosphere extract was generated by centrifuging an exponentially growing axenic *P*. cf. *balticum* culture (250 ml) at 1000 × *g* for 10 min to pellet the cells. The supernatant was then centrifuged again at 13,000 × *g* for 10 min to pellet the mucospheres. All the liquid was removed, and the pelleted mucus was extracted with 100% methanol (HPLC grade, total volume 4 ml), sonicated for 10 min on ice, before being incubated for 30 min in a thermomixer at 1000 rpm (room temperature). The extract was then centrifuged at 13,000 × *g* for 10 min (room temperature), and supernatant was aliquoted, dried using a vacuum concentrator (Vacufuge, Eppendorf, Hamburg, Germany) and the resulting dry weight was quantified.

The first chemotaxis assay involved an axenic culture of the eukaryotic prey *R. salina* in exponential growth. Ultra-filtered spent medium was used as a control in the ISCA, but also to resuspend the potential chemoattractants, maintaining the same water chemistry as the surrounding medium. This ultra-filtered spent medium was obtained by centrifuging 15 ml of *R. salina* culture at 1000 × *g* for 10 min, the supernatant was then filtered through a 0.2 μm Millex FG (Merck Millipore, USA); followed by two successive filtrations through a 0.22 μm Sterivex filter (Merck Millipore, USA), and finally through a 0.02 μm Anotop filter (Whatman, United Kingdom).

ISCAs (*n* = 5) were deployed in the *R. salina* culture (80 ml) for 1 h. At the end of the incubation, ISCA well contents were collected and preserved with paraformaldehyde (0.2% final concentration). Cell abundances in each ISCA treatment (*n* = 5) was quantified the same day by running a standardised volume of sample (30 µl) by flow cytometry (Beckman Coulter Cytoflex LX, Indianapolis, USA) using blue laser (488 nm) excitation and a combination of 690/50 nm and 585/42 nm detection with the CytExpert v2.4 software. To quantify the strength of chemotaxis, the chemotactic index (*Ic*) was calculated by dividing the number of cells present in each treatment by the number of cells present in the filtered seawater control^11,12^. Statistical significance was assessed using a two-sided t-test.

The second chemotaxis assay involved *P*. cf. *balticum*-associated bacteria. Ultra-filtered spent medium was used as a control in the ISCA (prepared with the assayed *P*. cf. *balticum* culture in the same way as above). ISCAs (*n* = 5) were deployed in *P*. cf. *balticum*-associated bacteria (80 ml) for 1 h^12^. These were obtained by centrifuging the culture at 1000 × *g* for 10 min to pellet the *P.* cf. *balticum* cells and using the supernatant. At the end of the incubation, ISCA well contents were collected and preserved with glutaraldehyde (2% final concentration) (Sigma Aldrich, St. Louis, Missouri, USA) for 15 min. Cell abundances in a standardised volume of sample (30 µl) each ISCA treatment (*n* = 5), was quantified by flow cytometry (Beckman Coulter Cytoflex LX, Indianapolis, USA) using blue excitation (488 nm) with SYBR 525/40 nm, Violet SSC detection and the CytExpert v2.4 software after first staining with SYBR Green I 10,000× (Invitrogen, Carlsbad, California, USA) (5:100,000 dilution) and incubating for 10 min. Statistical significance was assessed using a t-test.

### Mucosphere carbon contribution calculations

#### Carbon concentration of mucospheres

To quantify the carbon content of mucospheres, the axenic *P*. cf. *balticum* culture was grown under standard conditions in triplicate 75 cm^2^ (250 mL) sterile vented polystyrene tissue culture flasks (Falcon, Corning, New York, USA), inoculated at 5 × 10^3^ cells mL^−1^. The abundance of cells was monitored by subsampling 1 mL every 2–4 days for quantification on a flow cytometer using blue laser (488 nm) excitation, and a combination of 690/50 and 585/42 nm detection with CytExpert v2.4 software until the cells reached late exponential growth phase at ~4 × 10^4^ cells mL^−1^. The cells and mucus were harvested in the early morning when a large portion of the *P*. cf. *balticum* cells had abandoned the mucospheres. To further encourage separation of the dinoflagellate cells and mucospheres, the flasks were vigorously shaken before fractionation. A 15 mL subsample of the culture containing the spent medium, cells, and mucus was removed from each replicate flask and transferred into 15 mL centrifuge tubes (Fisher Scientific, Hampton, New Hampshire, USA). This comprised the “whole water” sample. A second 15 mL aliquot was taken from each culture flask and transferred to another 15 mL tube, was centrifuged at 1000 × *g* for 5 min to pellet the *P*. cf. *balticum* cells while leaving the mucus suspended in the spent medium supernatant. The supernatant containing the mucus and spent medium was transferred to a separate 15 mL tube and made up the “mucus and spent medium” sample. The remaining cell pellet was resuspended in 15 mL sterile natural seawater so the cells were in an equivalent volume of liquid for analysis—this comprised the “cell pellet” sample. This centrifugation process effectively separated the dinoflagellate cells from the mucus as shown in Supplementary Fig. 20 allowing the carbon of each fraction to be analysed individually. The final fraction consisted of only the “spent medium” and was obtained by repeating the process to separate the cells from the mucus, with the additional step of filtering the mucus from the spent medium using a syringe and reusable syringe filter holder (Sartorious, Göttingen, Germany) with a 25 mm 0.45 µm pore size nitrocellulose filter (Millipore, Burlington, Massachusetts, USA). This pore size was chosen as it is traditionally the threshold at which particulate (>0.4 µm) and dissolved (<0.4 µm) exopolymeric material are classified^20^. Control samples (5 replicates) were also collected and included sterile natural seawater as this was used to resuspend the harvested dinoflagellate cell pellet, and MilliQ water which had been filtered through the reusable syringe filter and 0.45 µm pore size nitrocellulose filter to account for the introduction of carbon during this process. All fractions were then stored frozen at −80 °C until carbon (C) analysis.

The C content of each of the fractions, including controls, was then measured using a Total Carbon Analyser (TOC-Lcsh model, Shimadzu, Kyoto, Japan) with the non-purgeable organic carbon (NPOC) protocol, a method that detects organic carbon. Upon thawing, 10 mL of sample was diluted 1:3 with MilliQ water. Additional procedural controls (5 replicates) of only MilliQ water were also analysed to determine the quantity of carbon introduced during the dilution process.

The quantity of carbon in each of the fractions was then calculated as follows and is presented in Table 2 and Supplementary Date File 1.

- C in all fractions − mean of C in MilliQ controls = Corrected C in all fractions
- C in spent medium filtrate − C in MilliQ 0.4 µm filter control = Corrected C in spent medium filtrate
- C in mucus and spent medium − corrected C in spent medium filtrate = Calculated C in mucus
- C in cell pellet fraction − C in natural seawater control = Corrected C in cell pellet fraction

A final check was also completed to determine the recovery of C in each of the fractions in comparison to the whole water fraction by:

- Corrected C in spent medium filtrate + Calculated C in mucus + Corrected C in cell pellet fraction = C in whole water
- Recovery of C in the cumulative fractions was found to be between 96 and 104% (Table 2).

**Table 2:** Measured POC concentrations in the collected fractions from the axenic *P*. cf. *balticum* culture.

Fraction	Replicate 1	Replicate 2	Replicate 3	Mean (SD)
C in MilliQ controls (mg L^−1^)	0.16 (0.04)*	0.16 (0.04)*	0.16 (0.04)*	0.16 (0.04)*
C in spent medium filtrate (mg L^−1^)	13.54	13.56	12.92	13.34 (0.36)
C in MilliQ 0.4 µm filter control (carbon introduced when removing the mucus from the spent medium filtrate) (mg L^−1^)	0.84 (0.08)*	0.84 (0.08)*	0.84 (0.08)*	0.84 (0.08)*
**C in corrected spent medium filtrate (spent medium filtrate – MilliQ 0.4 µm filter control) (mg L**^**−1**^**)**	**12.70**	**12.72**	**12.08**	**12.5 (0.36)**
C in mucus and spent medium filtrate (mg L^−1^)	**13.39**	**13.33**	**12.89**	**13.20 (0.27)**
**Calculated C in mucus (mucus and spent medium filtrate – corrected spent medium filtrate) (mg L**^**−1**^**)**	0.69	0.61	0.81	0.70 (0.10)
C in cell pellet fraction (mg L^−1^)	1.20	1.33	1.33	1.29 (0.08)
C in natural seawater control (mg L^−1^)	0.59 (0.11)*	0.59 (0.11)*	0.59 (0.11)*	0.59 (0.11)*
**Calculated C in cell pellet (cell pellet fraction – natural seawater control) (mg L**^**−1**^**)**	**0.61**	**0.74**	**0.73**	**0.69 (0.07)**
**Sum of C in all fractions (corrected spent medium filtrate + mucus + cell pellet) (mg L**^**−1**^**)**	**14.00**	**14.07**	**13.62**	**13.90 (0.24)**
C in whole water (mg L^−1^)	14.59	14.21	13.12	13.97 (0.76)
% Recovery of whole water C (mg L^−1^)	96%	99%	104%	99.67% (4.04)
**Calculated C in mucus in 10 mL sample adjusted for 1:3 dilution (mg 10 mL**^**−1**^ **sample) (multiplied by 0.03)**	**0.02**	**0.02**	**0.02**	**0.02**
**Estimated number of mucospheres in 10 mL of sample analysed**	**131,450**	**137,140**	**141,170**	**136,587 (4,833)**
**Carbon per mucosphere (mg)**	**1.6 × 10**^**−7**^	**1.3 × 10**^**−7**^	**1.7 × 10**^**−7**^	**1.53 × 10**^**−7**^ **(0.21 × 10**^**−7**^**)**
**Carbon per mucosphere (pg)**	**157.5**	**133.4**	**172.1**	**154.33 (19.54)**

Direct measures are shown in white rows; calculated estimates are shown in bold text.

^*^Mean of 5 replicates.

To determine the carbon associated with each mucosphere, the abundance of mucospheres in the harvested cultures was estimated. This was done by calculating the total number of cells produced over the growth period (8 days) and multiplying by the proportion of cells which produced mucospheres. This was estimated as 7.3% from a separate experiment in identical growth conditions, after monitoring a total of 422 individual *P*. cf. *balticum* cells in a 96 multi-well plate with 2–6 cells per well over 24 h (Table 3; Supplementary Data File 1). The carbon per mucosphere could then be calculated once the carbon in mg L^−1^ was converted to mg C per 10 mL sample (Table 2).

**Table 3:** *Prorocentrum* cf. *balticum* cell abundance from measured and calculated growth rate values over the growth period and the number of mucospheres estimated to have been in each mucus fraction for which carbon was quantified.

Cell count (mL)											
Rep.	Day 0	Day 1	Day 2	Day 3	Day 4	Day 5	Day 6	Day 7	Day 8	Sum	Sum in 10 mL
1	4,300	5,719	7,606	10,116	20,570	26,741	30,630	34,306	40,080	180,068	1,800,680
2	4,950	6,584	8,756	11,646	19,750	25,675	32,230	36,098	42,1800	187,868	1,878,680
3	5,000	6,650	8,845	11,763	19,100	24,380	34,510	38,651	44,040	193,389	1,933,890

Mucospheres (based on 7.3% production rate)											
1	314	417	555	738	1,502	1,952	2,236	2,504	2,926	13,145	131,450
2	361	481	639	850	1,442	1,874	2,353	2,635	3,079	13,714	137,140
3	365	485	646	859	1,394	1,813	2,519	2,822	3,215	14,117	141,170

#### *P*. cf. *balticum* abundance

We analysed multiple datasets to determine a conservative abundance of *P*. cf. *balticum* to use in our calculations. The first was part of the Joint Global Ocean Flux Study (JGOFS) and consisted of four cruises in May, June or July, of 1989, 1990 and 1991 (http://ijgofs.whoi.edu)^64^. We extracted the values for *P. minimum* (originally incorrectly reported as *P. minutum* in the JGOFS dataset) because this species is indistinguishable from *P*. cf. *balticum* using light microscopy and would have been the only species known at the time. Supplementary Fig. 17a shows the prevalence and abundance of this species in the surface samples collected as part of the JGOFS initiative. The abundance of *P. minimum* in the surface samples (~2 m as this was the only depth consistently measured on each cruise) varied with each cruise; May–July 1989: range 0–22,750 cells L^−1^, average of 5788 cells L^−1^ (*n* = 4); May–June 1990: range 270–64,940 cells L^−1^, average of 20,390 cells L^−1^ (*n* = 12); June 1991: range 0–24,700 cells L^−1^, average of 3743 cells L^−1^ (*n* = 42); and July 1991: range 0–13,300 cells L^−1^, average of 2744 cells L^−1^ (*n* = 36).

The second dataset was from the Continuous Plankton Recorder (CPR) which comprised plankton light microscope count data spanning from 1958 to 2018 (DOI:10.17031/1735)^65^. In this dataset, pelagic *Prorocentrum* species were differentiated into several categories but the small round pelagic species with similar morphology as *P. minimum* were grouped into a category named “*Prorocentrum exuviaella* type” based on the early classification of these species in the genus *Exuviaella*^65^. The abundances in this dataset represent surface samples from 0 to 10 m and cannot be directly converted to cells L^−1^, though the samples were collected consistently through time, so the values do represent relative changes. The data are plotted in Supplementary Fig. 17b.

The third dataset was the Australian Integrated Marine Observing System National Reference Station Programme (IMOS-NRSP) microscope identification and enumeration data, which includes monthly counts of marine phytoplankton for over a decade at nine stations off the continental shelf around Australia spanning tropical and temperate locations across latitudes from −12.24 to −42.35°^66^. The samples for phytoplankton microscope identification and enumeration from the IMOS-NRSP, are collected by combining water from 0 to 50 m to provide a pooled sample that is more representative of cell abundances across the upper ocean. Because of the difficulty in distinguishing *P*. cf. *balticum* cells from the closely related and morphologically similar *P. minimum* species using light microscopy, cell abundance is reported as a value for the *Prorocentrum minimum/balticum* complex (shown as *Prorocentrum* spp. in the dataset). The lowest and highest station average cell number of *P. minimum/balticum* complex over the decade long dataset was 19 and 137 cells L^−1^ which were from Maria Island and Kangaroo Island, respectively, and the maximum cell abundance reached was 3350 cells L^−1^ at the Port Hacking station (Supplementary Data File 1; Supplementary Fig. 18). Given that our strains were isolated from Port Hacking, one of the IMOS-NRSP Stations, and these values are consistently lower than those reported in other datasets, we used these conservative estimates to represent the annual mean abundance of *P*. cf. *balticum* in oceanic waters. We therefore used the values of 19 and 137 cells L^−1^ to represent the annual mean abundance of *P*. cf. *balticum* to calculate a mean range of potential carbon contributions but also the maximum cell number (3350 cells L^−1^) to allow calculation of the maximum potential carbon contribution if abundances were to reach bloom proportions.

#### Carbon calculations

With an estimate of the carbon content per mucosphere and the mean and maximum number of *P*. cf. *balticum* cells in the ocean, we then calculated the export potential of mucosphere production (Supplementary Data File 1). We used the same approach as (Alldredge et al.^23^ and references therein), to estimate the potential for carbon export by mucospheres. First, we determined a representative depth for the euphotic zone across the global ocean. We plotted the Photosynthetically Active Radiation (PAR) (µmol m^−2^ s^−1^) values collected as part of the *Tara* Oceans initiative^67^ as a function of depth (Supplementary Fig. 19). The lowest light level for which we measured mucosphere production rates was 20 µmol m^−2^ s^−1^ (low light level) (see the methods section titled ‘Mucosphere production experiments’), and the depth at which this light level was reached in the *Tara* Oceans PAR data was 60 m. We, therefore, used 60 m to represent the depth of the euphotic zone across the global ocean for the purpose of our mucosphere production carbon contribution calculations. Beginning with 1 m^2^ of ocean area, we multiplied by 60 m to represent 1 m^2^ of ocean volume in the euphotic zone as 60 m^3^. We then converted this to litres for 6 × 10^4^ L m^−2^. The lowest and highest station average cell abundances of the *P. minimum/balticum* complex of 19 and 137 cells L^−1^, were multiplied by this ocean volume for 1.14 × 10^6^–8.22 × 10^6^ cells m^−2^. Assuming a 23% production rate of mucospheres in the presence of eukaryotic prey under low light conditions, as measured through experimentation in this study (Supplementary Table 2; Note: this is different to the 7.3% mucosphere production rate found in the absence of prey, used in calculations for evaluating the carbon content of mucospheres in the methods section titled ‘Carbon concentration of mucospheres’), the total number of mucospheres that could be produced daily across the ocean was estimated to be 2.62 × 10^5^–1.89 × 10^6^ mucospheres m^−2^ d^−1^. With an average of 154.3 pg of carbon per mucosphere, that equates to 0.04–0.29 mg C m^−2^ d^−1^ produced and released in the form of mucospheres. When these calculations are completed using the maximum cell abundance reported in the IMOS-NRSP dataset (3350 cells L^−1^), these values increase to 7.13 mg C m^−2^ d^−1^. Converting these values to represent a daily carbon production rate across the surface area of the world’s oceans (3.24 × 10^8^ km^2^ excluding the polar regions^68^), mucospheres potentially contribute 1.31 × 10^−5^–9.46 × 10^−5^ Gt C d^−1^ or 0.005–0.035 Gt C y^−1^. We did not calculate the daily or yearly carbon contribution potential for the maximum *P*. cf. *balticum* cell abundance as these values represent bloom conditions and it is unrealistic to consider these values could be reached across the global ocean and sustained year-round.

The carbon concentrations used for these calculations were measured using the axenic *P*. cf. *balticum* culture and therefore represent only the carbon content of the mucus. The attractive and adhesive nature of the mucus provides an opportunity for additional carbon export by trapping other marine microbes. The amount of carbon this additional process could contribute was calculated by estimating the number of prokaryotes and eukaryotes that could be captured in a mucosphere and adding the carbon content of these cells. The average mucosphere is 100 µm in diameter which represents a volume of 524,000 µm^3^ or 5.24 × 10^−7^ mL (approximately 5 × 10^−5^ % of 1 mL). Assuming an abundance of 1 × 10^6^ cells mL^−1^ of bacteria in seawater^69^, a conservative estimate of the number of bacteria in a mucosphere based on area alone (without attraction) would be 52 cells (5 × 10^−5^ % of 1 × 10^6^ cells mL^−1^). Using the mean carbon content of a bacterium of 20 fg C cell^−1^ estimated by^70^, the additional carbon contributed by 52 bacteria cells in a mucosphere is 1.05 × 10^3^ fg C. Using the theory that the carbon content of cells scale with cell volume^71^, and assuming 10 spherical cells of 10 µm diameter with a carbon content of ~50 pg cell^−1^, we estimated eukaryotic cells captured in mucospheres could contribute an additional 5 × 10^2^ pg C. Therefore, the total potential for carbon export by mucospheres laden with prokaryotic and eukaryotic prey is 5.57 × 10^−5^–4.02 × 10^−4^ Gt C d^−1^ or 0.02–0.15 Gt C y^−1^ when calculated using the lowest and highest station average *P*. cf. *balticum* cell abundance and 9.83 × 10^−3^ Gt C d^−1^ when calculated using the maximum *P*. cf. *balticum* cell abundance recorded.

### Statistics and reproducibility

No sample size calculations were performed. The number of replicates in each experiment was chosen based on pilot studies. Mucosphere production by *P.* cf. *balticum* cells was observed consistently throughout all experiments. Morphological assessments were performed on 30 individual cells. Intracellular food vacuoles resulting from the consumption of prokaryotic or eukaryotic prey by *P*. cf. *balticum* was observed in at least *n* = 5 cells. Prey capture in mucospheres and survival after 24 h of immobilisation was confirmed at least *n* = 3 times. Negative buoyancy of mucospheres was confirmed *n* = 3 times. The stages of sexual reproduction were observed in at least *n* = 5 cells. Positive or negative staining of mucospheres was observed for at least *n* = 3 mucospheres. Separation of *P*. cf. *balticum* cells and mucospheres by centrifugation was optimised through repetition and observed in at least *n* = 3 samples. Further information about statistical analysis is shown in the relevant methods sub-sections.

### Reporting summary

Further information on research design is available in the Nature Research Reporting Summary linked to this article.

## Supplementary information

Supplementary Information File

Description of Additional Supplementary Files

Supplementary Movie 1

Supplementary Data 1

Reporting Summary

## Data Availability

The xenic and axenic *P.* cf. *balticum* cultures established in this study have been deposited at the Australian National Algal Culture Collection (ANACC) under accession CS-1390 and are available for distribution. Sequences from the phylogenetic analysis of the four *P.* cf. *balticum* strains have been deposited in Genbank with accession numbers LSU MW024106-MW02409; SSU MW024110-MW024113; ITS1 MW024089-MW02492 and SSU-ITS-LSU MW024115-MW024118. The raw.fastq read files from the *P.* cf. *balticum* associated microbiome assessment were deposited in Sequence Read Archive (SRA) under project id PRJNA737517 with sample numbers SAMN19697965-SAMN19697985 (https://www.ncbi.nlm.nih.gov/sra/?term=PRJNA737517). Raw data associated with the mucosphere carbon contribution calculations are supplied as a supplementary excel file titled Supplementary Data File 1. A movie file showing phago-heterotrophic feeding and mucosphere construction is provided as a supplementary.mp4 file titled Supplementary Movie File 1. The data used to assess the distribution and abundance of *P.* cf. *balticum* can be accessed through the following links: Integrated Marine Observing System National Reference Station Programme (IMOS-NRSP), through the Australia Ocean Data Network (AODN) portal https://portal.aodn.org.au/; Joint Global Ocean Flux Study (JGOFS), DOI:10.1594/PANGAEA.859221; Continuous Plankton Recorder (CPR) Survey, DOI:10.17031/1735; The *Tara* Oceans amplicon dataset, DOI:10.1594/PANGAEA.873275 and DOI:10.1594/PANGAEA.875582

## Code availability

The R code used for the *P.* cf. *balticum*-associated microbiome analysis is available at https://github.com/ARBramucci/Larsson2020.
