# Supplementary material for: Mucospheres produced by a mixotrophic protist impact ocean carbon cycling
Source: Nat Commun. 2022 Mar 14;13:1301. doi: 10.1038/s41467-022-28867-8 (PMC8921327; doi:10.1038/s41467-022-28867-8)
Supplement: Supplementary file 1 — Supplementary Information File [file 41467_2022_28867_MOESM1_ESM.pdf]

Nature Communications

Supplementary Information file for

Mucospheres produced by a mixotrophic protist impact ocean carbon cycling

Authors: Michaela E. Larsson[1]*, Anna R. Bramucci[1], Sinead Collins[2], Gustaaf Hallegraeff[3], Tim Kahlke[1], Jean-Baptiste Raina[1], Justin R. Seymour[1], Martina A. Doblin[1]

Affiliations:

[1]Climate Change Cluster (C3), University of Technology, Sydney, PO Box 123, Broadway NSW 2007, Australia.

[2]Institute of Evolutionary Biology, School of Biological Sciences, University of Edinburgh, Edinburgh, UK.

[3]Institute for Marine and Antarctic Studies, University of Tasmania, Private Bag 129 Hobart, TAS 7001, Australia

[*]Corresponding author:

Dr. Michaela E. Larsson

Michaela.Larsson@uts.edu.au

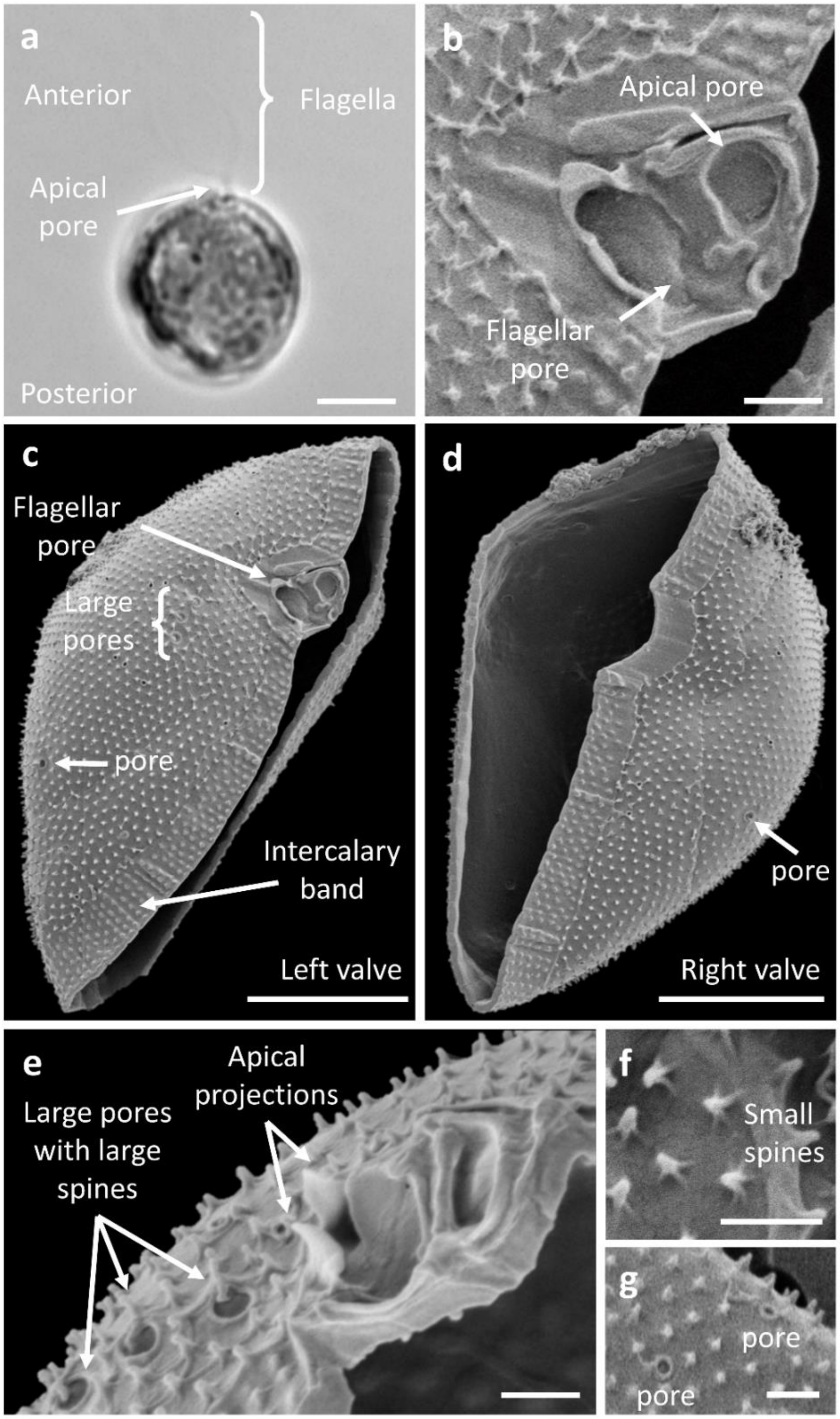

Supplementary Figure 1. Light microscope and Scanning Electron Microscope images of *P*. cf. *balticum* demonstrating the morphological features that differentiate it from other described *Prorocentrum* species. (a) light microscope image showing cell structure, and flagella placement. (b-g) SEM images showing the detail of the apical area, the large and small pores, large and small spines and ornamented intercalary band as described in Supplementary Note 1. Scale bars in (a, c-d) = 5 µm. Scale bar in (b, e-g) = 0.5 µm.

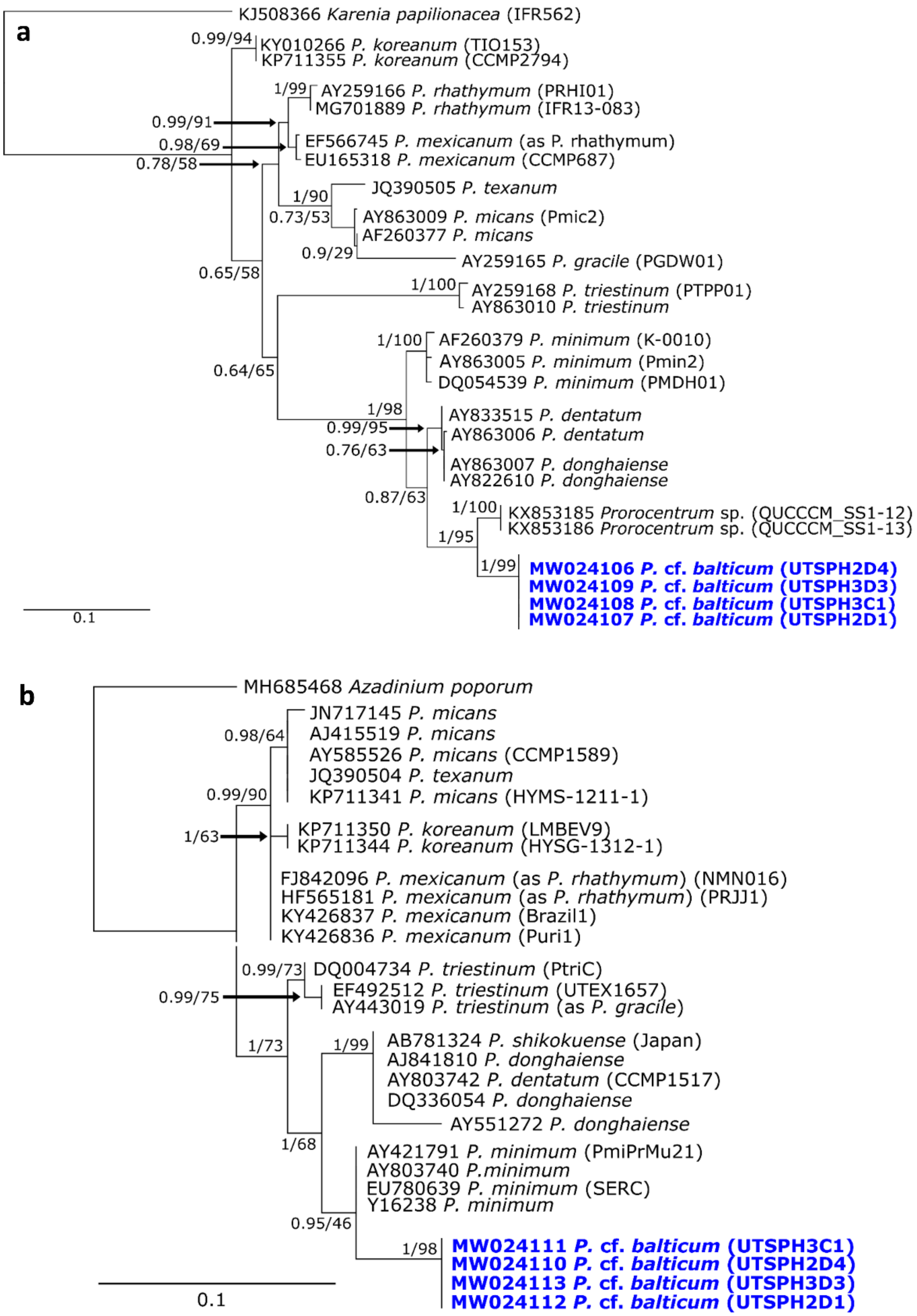

Supplementary Figure 2. The maximum likelihood phylogenetic tree showing alignment of the (a) D1-D3 region of the 28S large subunit (LSU) and the (b) 18S small subunit (SSU) rDNA sequences for *P.* cf. *balticum*. Strains from this study are shown in blue. Accession numbers, species designation and strain codes are provided for each sequence and values at the nodes represent Bayesian posterior probability and Maximum Likelihood bootstrap support.

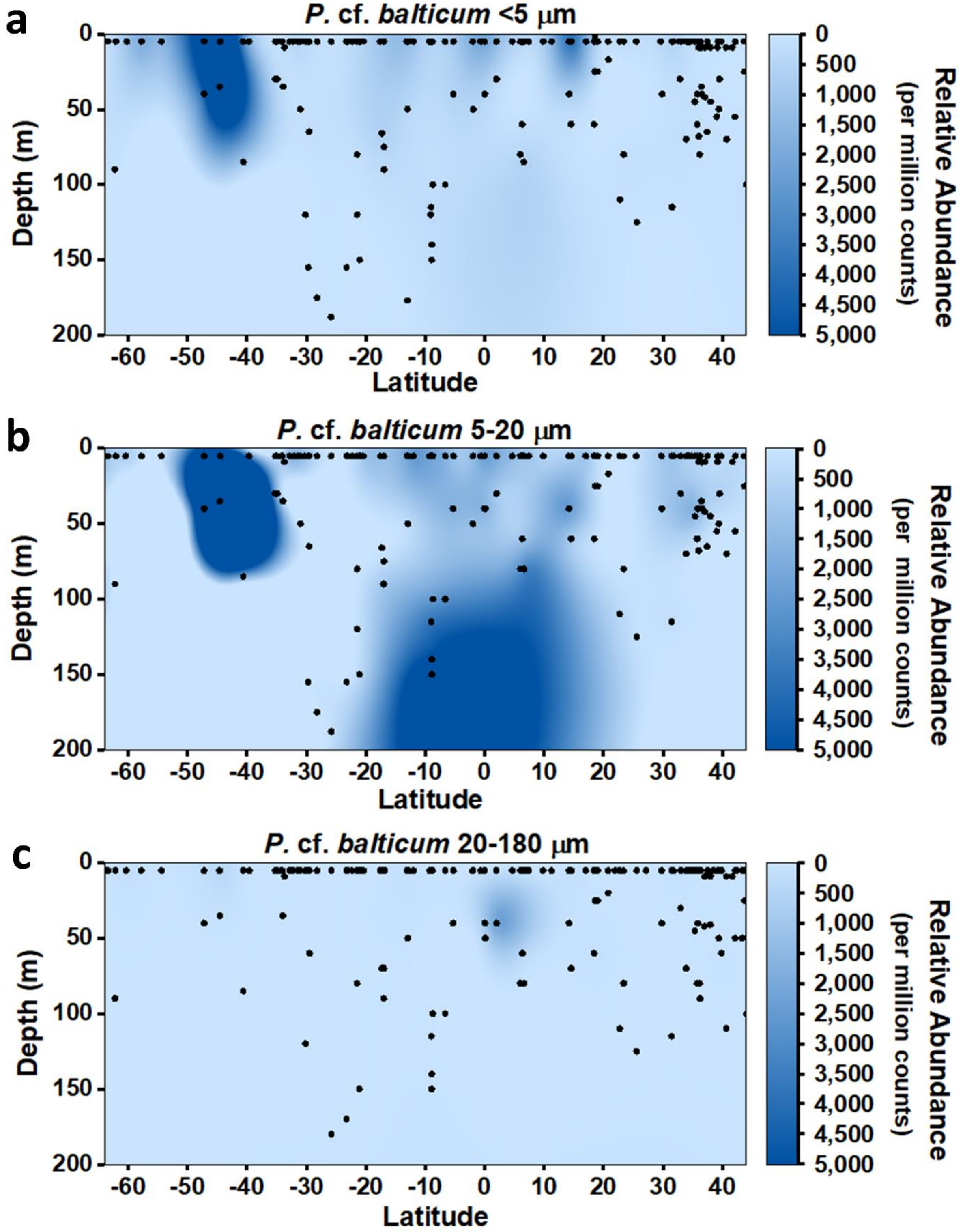

Supplementary Figure 3. Relative abundance of the *P.* cf. *balticum* across latitude and depth for three size fractions (a) <5 um, (b) 5-20 um and (c) 20-180 um in the Tara Oceans dataset. Note the Tara Oceans campaign collected samples from the surface (typically 5 m and the deep chlorophyll maximum (which varied from 17-188 m depth and was identified by in-situ fluorometry).

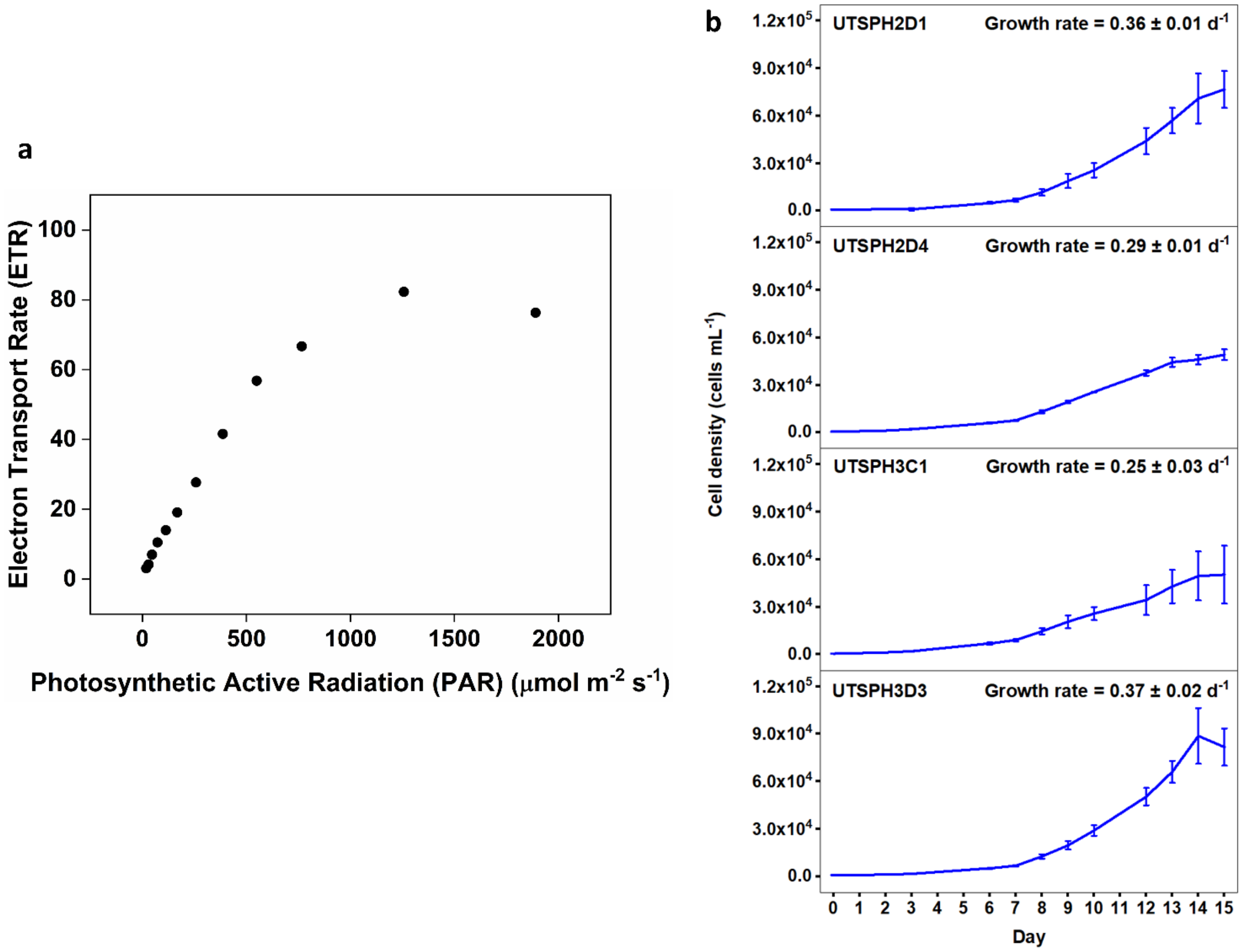

Supplementary Figure 4. (a) Rapid Light Curve acquired using a Pulse Amplitude Modulated (PAM) Fluorometer (Water-PAM, Walz, Germany) for *P.* cf. *balticum* strain UTSPH3D3 demonstrating the photosynthetic capacity of the mixotrophic dinoflagellate. (b) shows phototrophic growth curves of the four established *P.* cf. *balticum* strains isolated from the Australian Integrated Marine Observing System (IMOS) Port Hacking (PH100m) station. Mean ± standard deviation (n = 3 biological replicates). Maximum growth rates (d^{-1}) are shown next to the strain code. Strain UTSPH3D3 was selected for subsequent experiments.

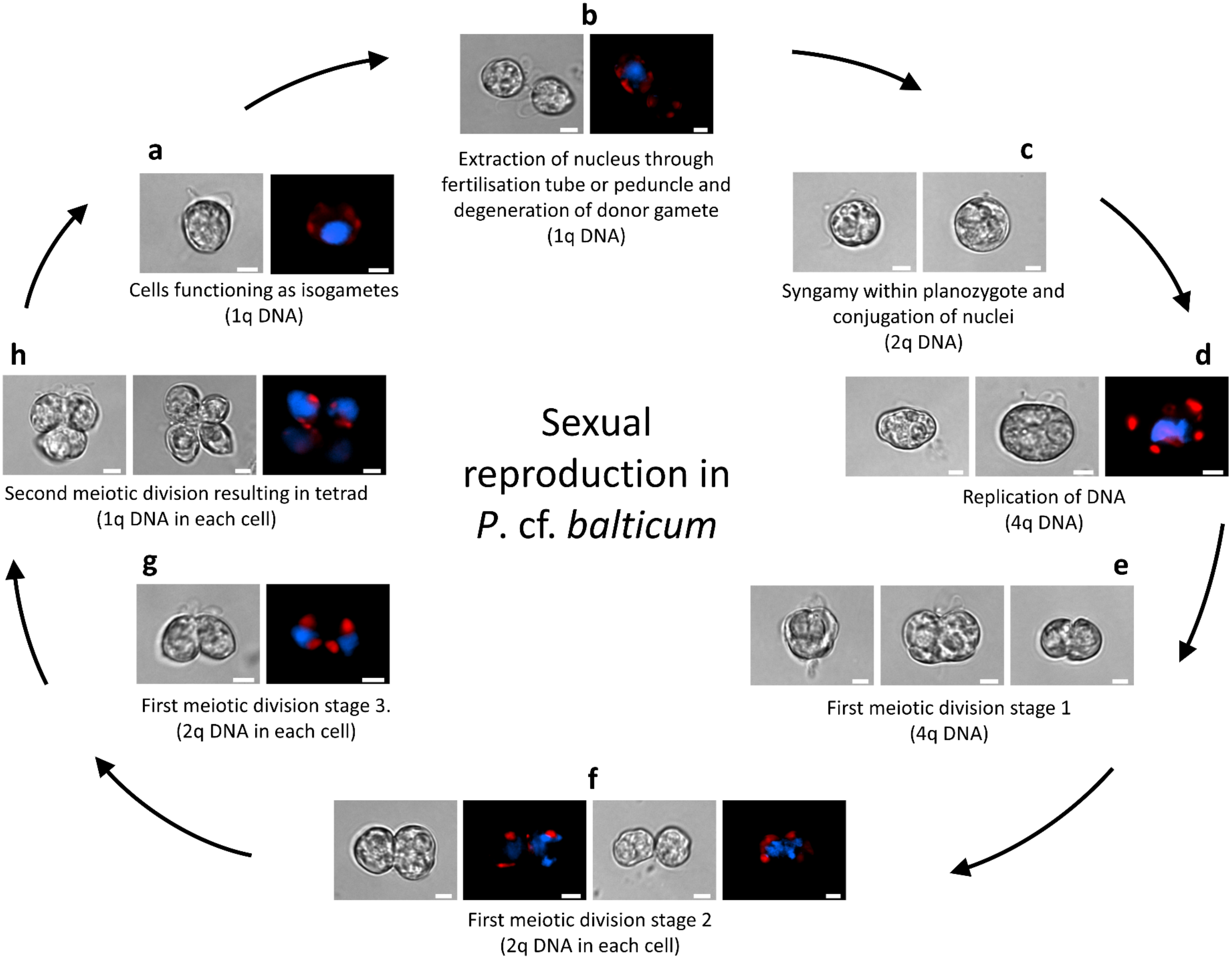
b
Extraction of nucleus through fertilisation tube or peduncle and degeneration of donor gamete (1q DNA)
c
Syngamy within planozygote and conjugation of nuclei (2q DNA)
d
Replication of DNA (4q DNA)
e
First meiotic division stage 1 (4q DNA)
f
First meiotic division stage 2 (2q DNA in each cell)
g
First meiotic division stage 3. (2q DNA in each cell)
h
Second meiotic division resulting in tetrad (1q DNA in each cell)
a
Cells functioning as isogametes (1q DNA)
Sexual reproduction in *P*. cf. *balticum*

Supplementary Figure 5. Sexual reproduction cycle of *P*. cf. *balticum* including the stages of (a) isogamete formation; (b) nucleus extraction through the fertilisation tube or peduncle; (c) conjugation of nuclei; (d) DNA replication; (e-g) first meiotic division; and (h) second meiotic division; observed under nutrient limited conditions with plastids shown in red and nucleus stained with 4',6-diamidino-2-phenylindole (DAPI) and shown in blue. Images were taken on an inverted fluorescence microscope (Nikon Eclipse Ti), fitted with Nikon FITC 480/30nm ex 535/45nm em, Texas Red 560/40nm ex 630/60nm em and DAPI 375/28nm ex 460/60nm em filters and a black and white camera (Nikon DS-QiMc) under 400x magnification. Scale bar = 5 µm.

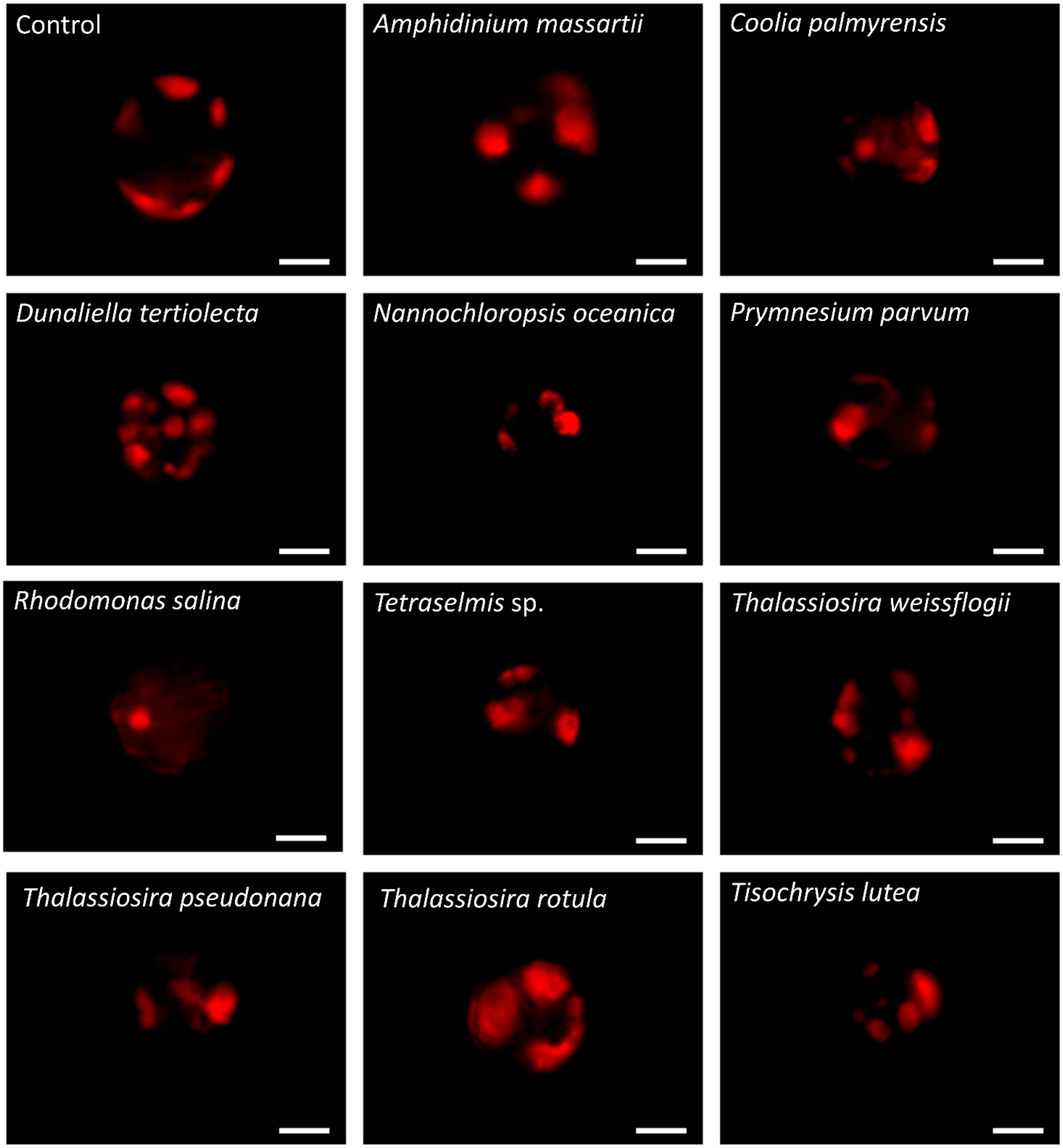

Supplementary Figure 6. Fluorescent microscope images of red-fluorescent intracellular food vacuoles within *P.* cf. *balticum* following phago-heterotrophic consumption of various unicellular eukaryotic prey immobilised in mucospheres following a 48h co-incubation period. Food vacuoles were defined as spherical red bodies found within the cell interior usually devoid of chloroplasts in *P.* cf. *balticum*. Images were taken using an inverted fluorescence microscope (Nikon Ti, Japan) with Texas Red 560/40nm ex 630/60nm em filter and a black and white camera (Nikon DS-QiMc 12 bit) under 400x magnification. Scale bar = 5 µm.

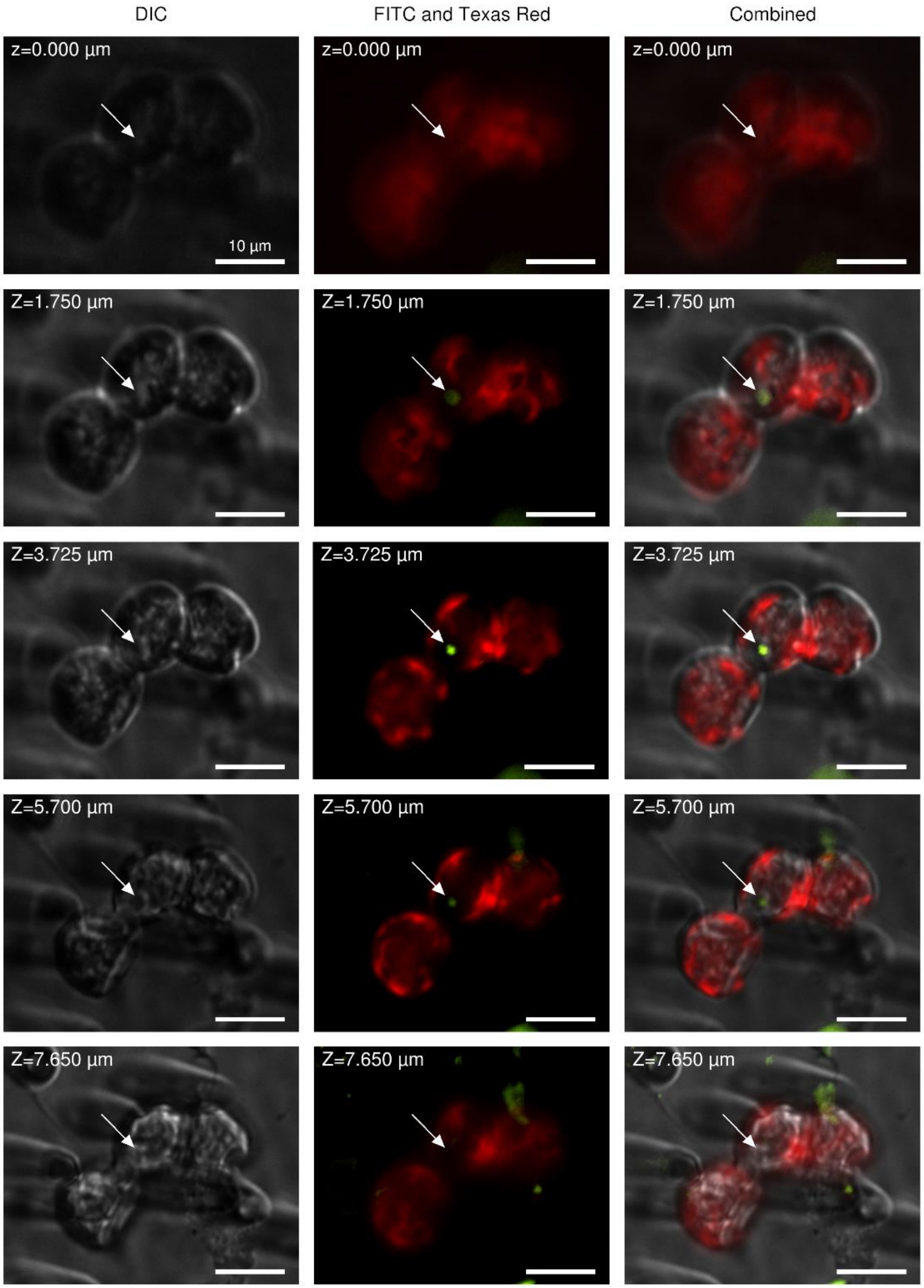

Supplementary Figure 7. A z-stack of fluorescent microscope images showing a bacterial cell fluorescently labelled with 5-([4,6-dichlorotriazin-2-yl]amino)fluorescein hydrochloride (DTAF) ingested by *P.* cf. *balticum*. Images were taken using an inverted fluorescence microscope (Nikon Eclipse Ti, Japan) fitted with Texas Red 560/40nm ex 630/60nm em and FITC 480/30nm ex 535/45nm em filters and a black and white camera (Nikon DS-QiMc 12 bit) under 400x magnification. Scale bar = 10 µm.

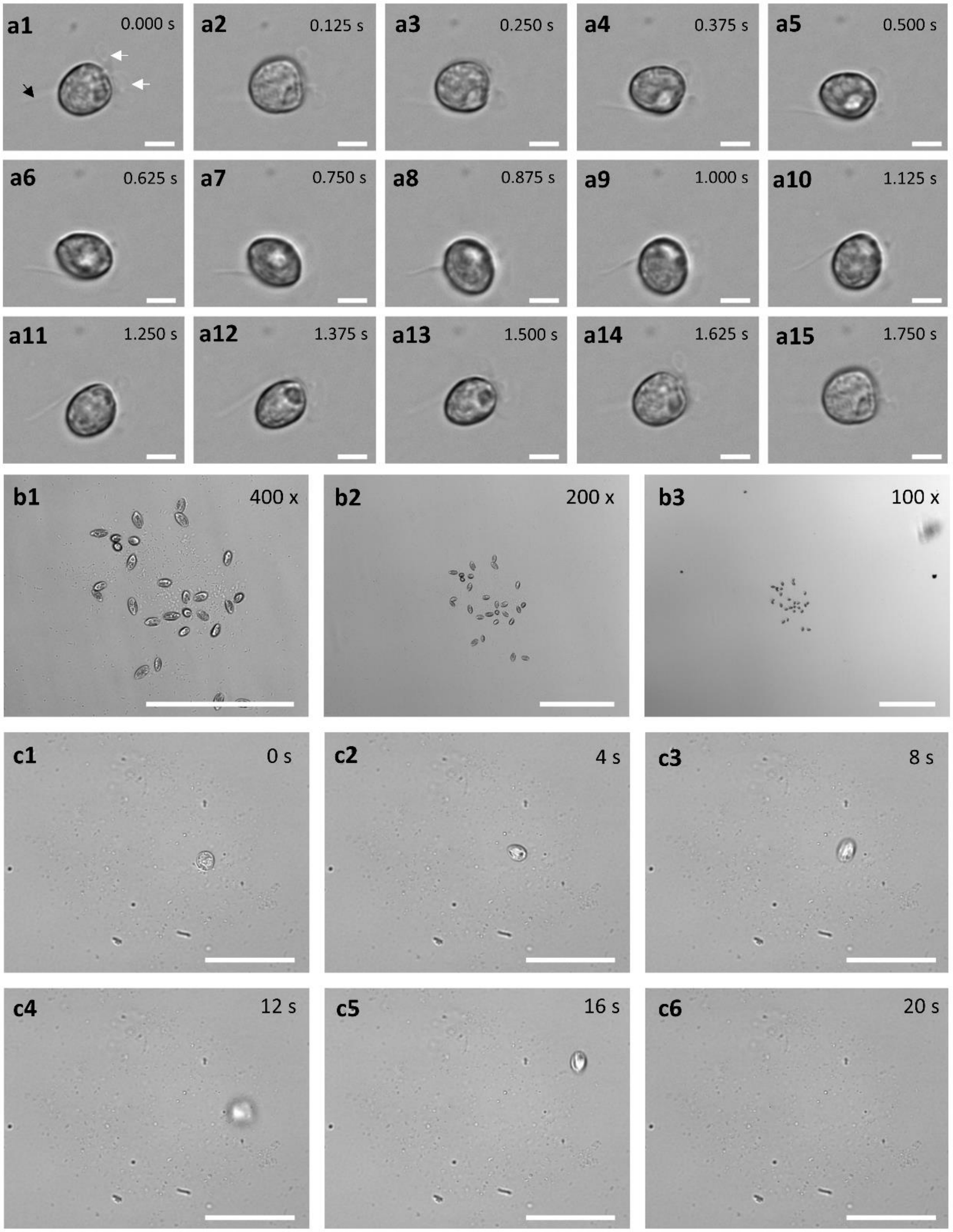

Supplementary Figure 8. (a) *P.* cf. *balticum* twisting to make a mucosphere. (b) are images of both prokaryotic and eukaryotic (*R. salina*) prey cells attracted to mucus and/or exudates from *P.* cf. *balticum* at different objective magnifications, and (c) is a series of images showing the ease with which a *P.* cf. *balticum* cell can leave a mucosphere after stimulation with fluorescent light. Images were taken on an inverted fluorescence microscope (Nikon Eclipse Ti) with a black and white camera (Nikon DS-QiMc) under 100, 200 or 400x magnification. Scale bar in (a) = 5 µm, (b) = 50 µm and (c) = 100 µm.

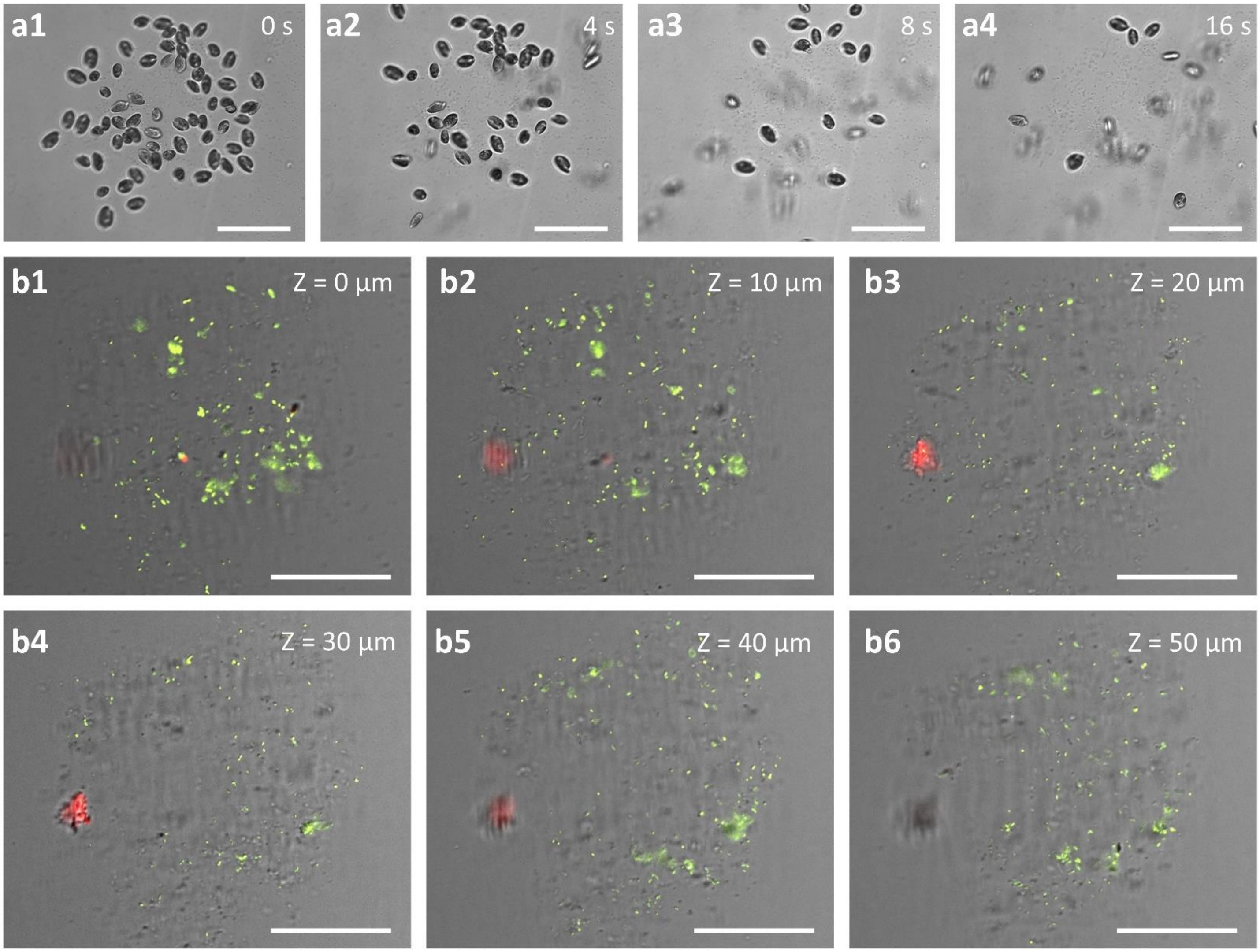

Supplementary Figure 9. (a1) Eukaryotic prey (*R. salina*) accumulated in a mucosphere after 24 hour, (a2-a4) shows the eukaryotic prey struggling free from the mucosphere after stimulation with fluorescent light which activated/irritated their photosynthetic apparatus. This demonstrates the cells remain alive while being immobilised in the mucosphere. (b1-b6) is a series of Z-level images through a mucosphere with the prokaryotes stained using the DNA stain SYBR Green I (green) and live/dead stain Propidium Iodide (PI). Both live and dead cells emit a green fluorescence signal from the SYBR Green I stain, but dead cells would also emit an orange fluorescence signal from the PI penetrating intracellularly. Most prokaryotic cells were only green demonstrating that the cells are alive after 24 hours captured in a mucosphere. Images were taken on an inverted fluorescence microscope (Nikon Eclipse Ti), fitted with Nikon FITC 480/30nm ex 535/45nm em and Texas Red 560/40nm ex 630/60nm em filters and a black and white camera (Nikon DS-QiMc) under 200x magnification. Note that the FITC and Texas Red images have been overlaid for visual comparison in b1-b6. Scale bar = 50 µm.

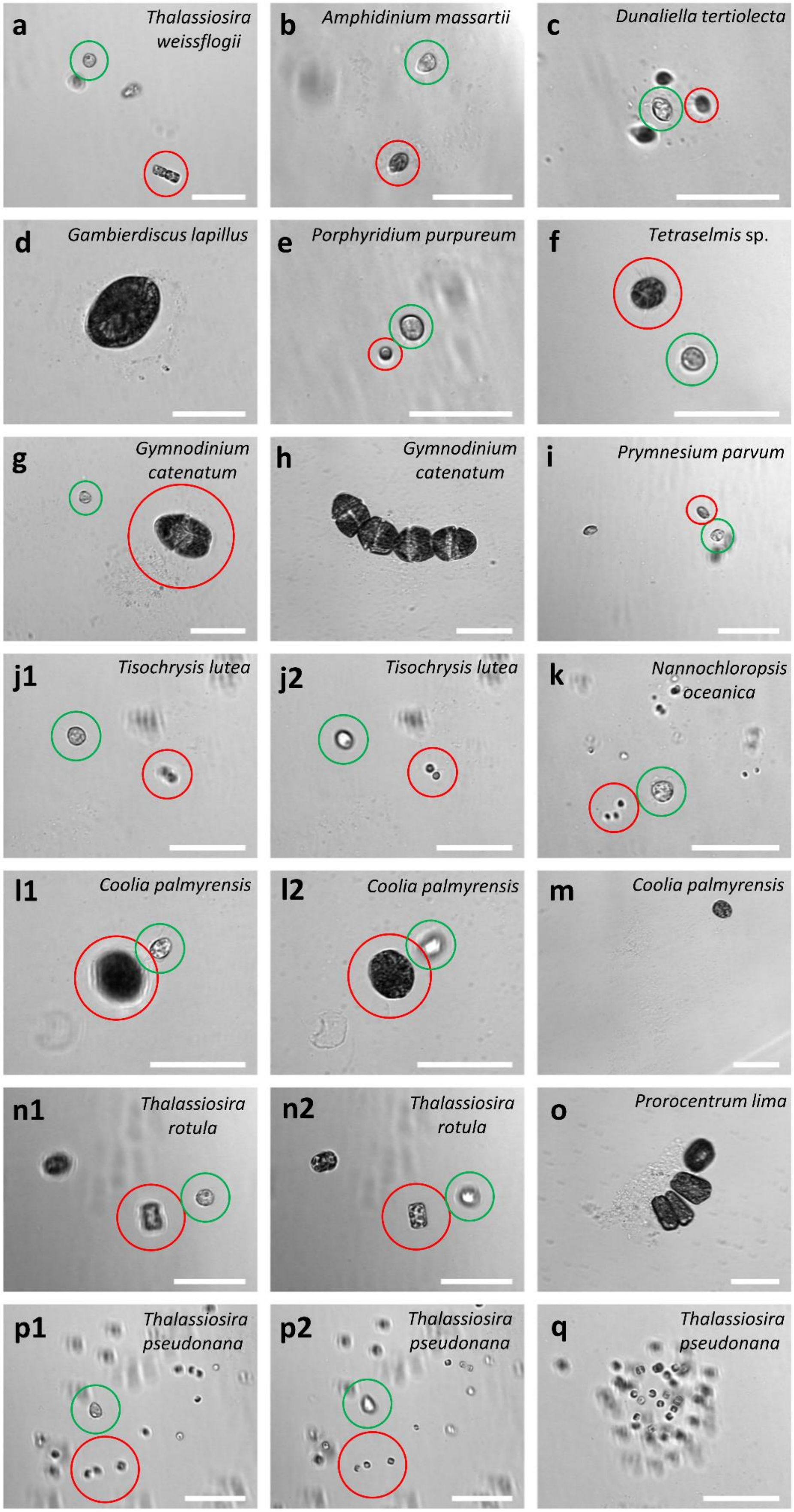
a Thalassiosira weissflogii
b Amphidinium massartii
c Dunaliella tertiolecta
d Gambierdiscus lapillus
e Porphyridium purpureum
f Tetraselmis sp.
g Gymnodinium catenatum
h Gymnodinium catenatum
i Prymnesium parvum
j1 Tisochrysis lutea
j2 Tisochrysis lutea
k Nannochloropsis oceanica
l1 Coolia palmyrensis
l2 Coolia palmyrensis
m Coolia palmyrensis
n1 Thalassiosira rotula
n2 Thalassiosira rotula
o Prorocentrum lima
p1 Thalassiosira pseudonana
p2 Thalassiosira pseudonana
q Thalassiosira pseudonana

Supplementary Figure 10. Various eukaryotic prey species (a) *Thalassiosira weissflogii*; (b) *Amphidinium massartii*; (c) *Dunaliella tertiolecta*; (d) *Gambierdiscus lapillus*; (e) *Porphyridium purpureum*; (f) *Tetraselmis* sp. (g-h) *Gymnodinium catenatum*; (i) *Prymnesium parvum*; (j) *Tisochrysis lutea*; (k) *Nannochloropsis oceanica*; (l-m) *Coolia palmyrensis*; (n) *Thalassiosira rotula*; (o) *Prorocentrum lima*; (p-q) *Thalassiosira pseudonana;* immobilised in mucospheres captured during experiments assessing prey consumption capabilities. Green circles show *P.* cf. *balticum* cells and red circle show prey species. Note images j1-j2, l1-l2, n1-n2, p1-p2 are the same cells but with either the *P.* cf. *balticum* cell or the prey cell in focus. Images were taken on an inverted fluorescence microscope (Nikon Eclipse Ti) with a black and white camera (Nikon DS-QiMc) under 200 or 400x magnification. Scale bars = 50 µm.

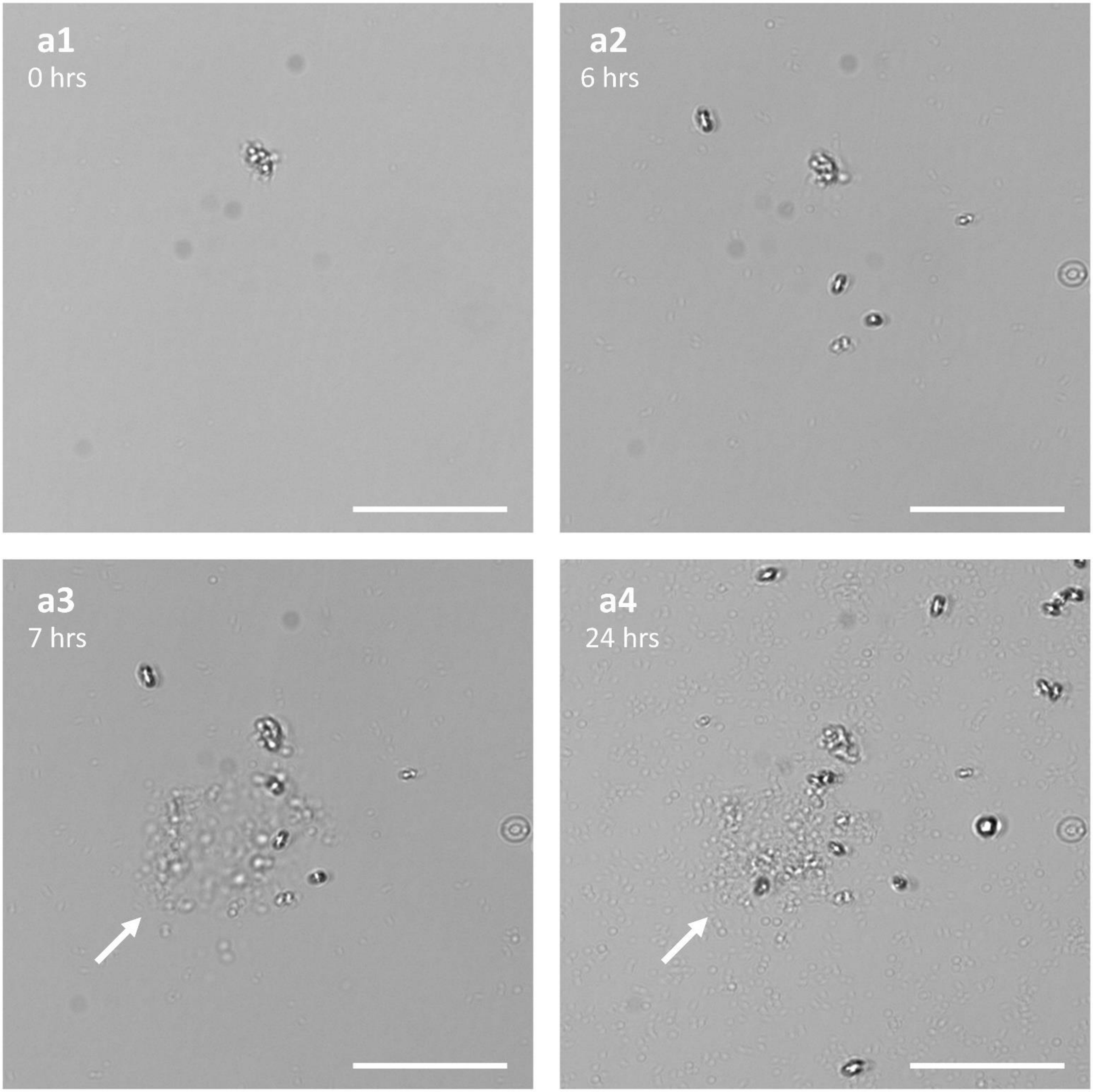

Supplementary Figure 11. A series of brightfield images through time of the same field of view taken at intervals over 24 hours (a1 = 0 hours; a2 = 6 hours; a3 = 7 hours; and a4 = 24 hours) using the INCell High Content Analyzer 2200 automated microscope system using a 10× objective demonstrating the negative buoyancy and propensity for sinking of a mucosphere laden with excess eukaryotic (*Rhodomonas salina*) and prokaryotic prey cells. The sinking mucosphere (arrow) is visible from 7 hours onward (a3-a4). Scale bar = 100 μm

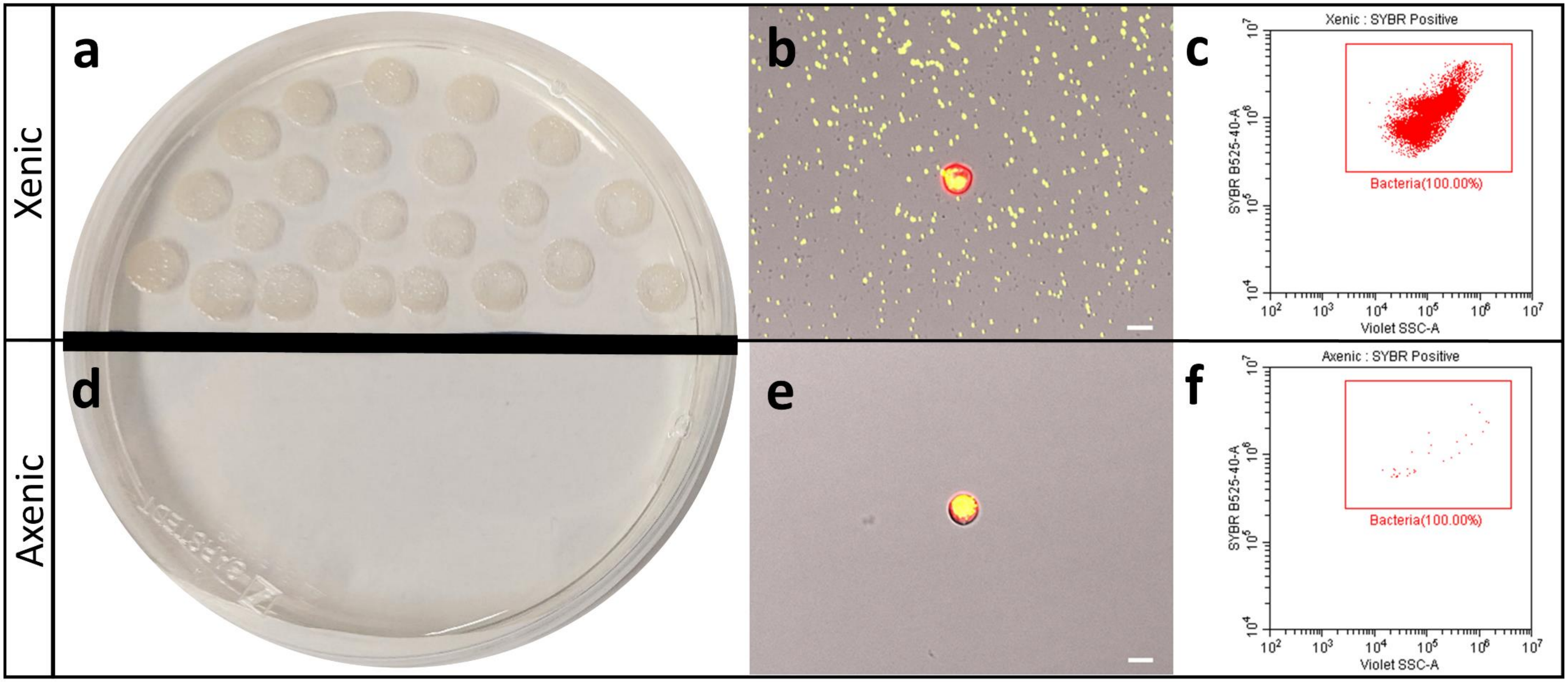

Supplementary Figure 12. Verification of axenicity for *P.* cf. *balticum* strain UTSPH3D3. (a) and (d) shows bacterial growth on marine agar after 7 days. (b) and (e) are images showing a single red-fluorescent *P.* cf. *balticum* cell with or without bacteria stained with the DNA stain SYBR Green I (green) taken on an inverted fluorescence microscope (Nikon Eclipse Ti), fitted with Nikon FITC 480/30nm ex 535/45nm em and Texas Red 560/40nm ex 630/60nm em filters and a black and white camera (Nikon DS-QiMc) under 200x magnification. Scale bar = 10 µm. (c) and (f) highlights the presence of bacteria stained with SYBR Green visualized using flow cytometry and CytExpert v2.4 software (Beckman Coulter Cytoflex LX, Indianapolis, USA) with a blue laser (488 nm) excitation and detected with a SYBR 525/40 nm filter.

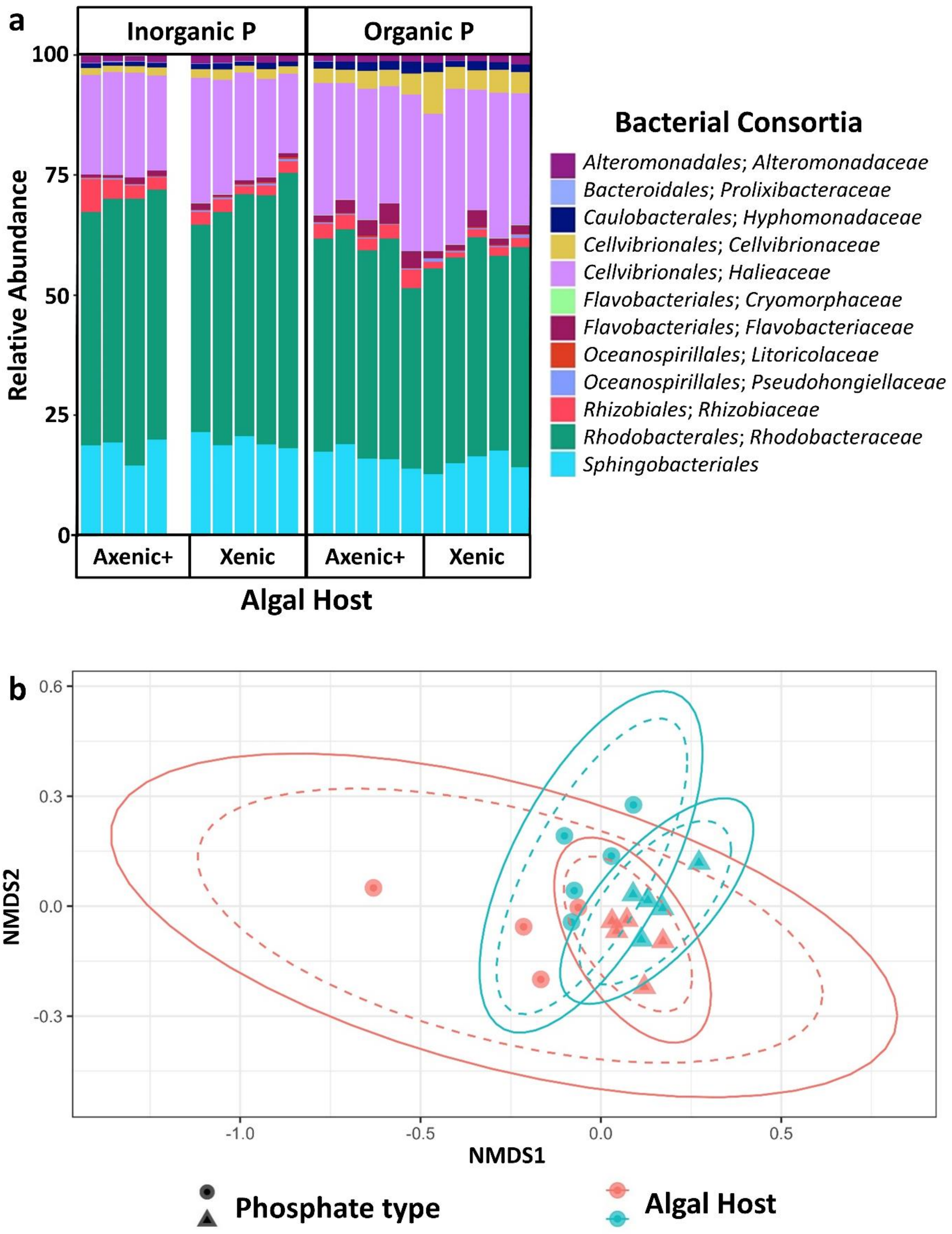

Supplementary Figure 13. Figure showing comparison of the bacterial consortia of the xenic and axenic+ *P.* cf. *balticum* lineages. (a) shows relative abundance of rarified microbiome amplicon sequence variance (ASVs). (b) is a Non-Multidimensional Scaling (NMDS) plot also of the rarified ASVs where the dashed ellipses show the type norm 95% confidence intervals, and the solid ellipses show the type t 95% confidence intervals.

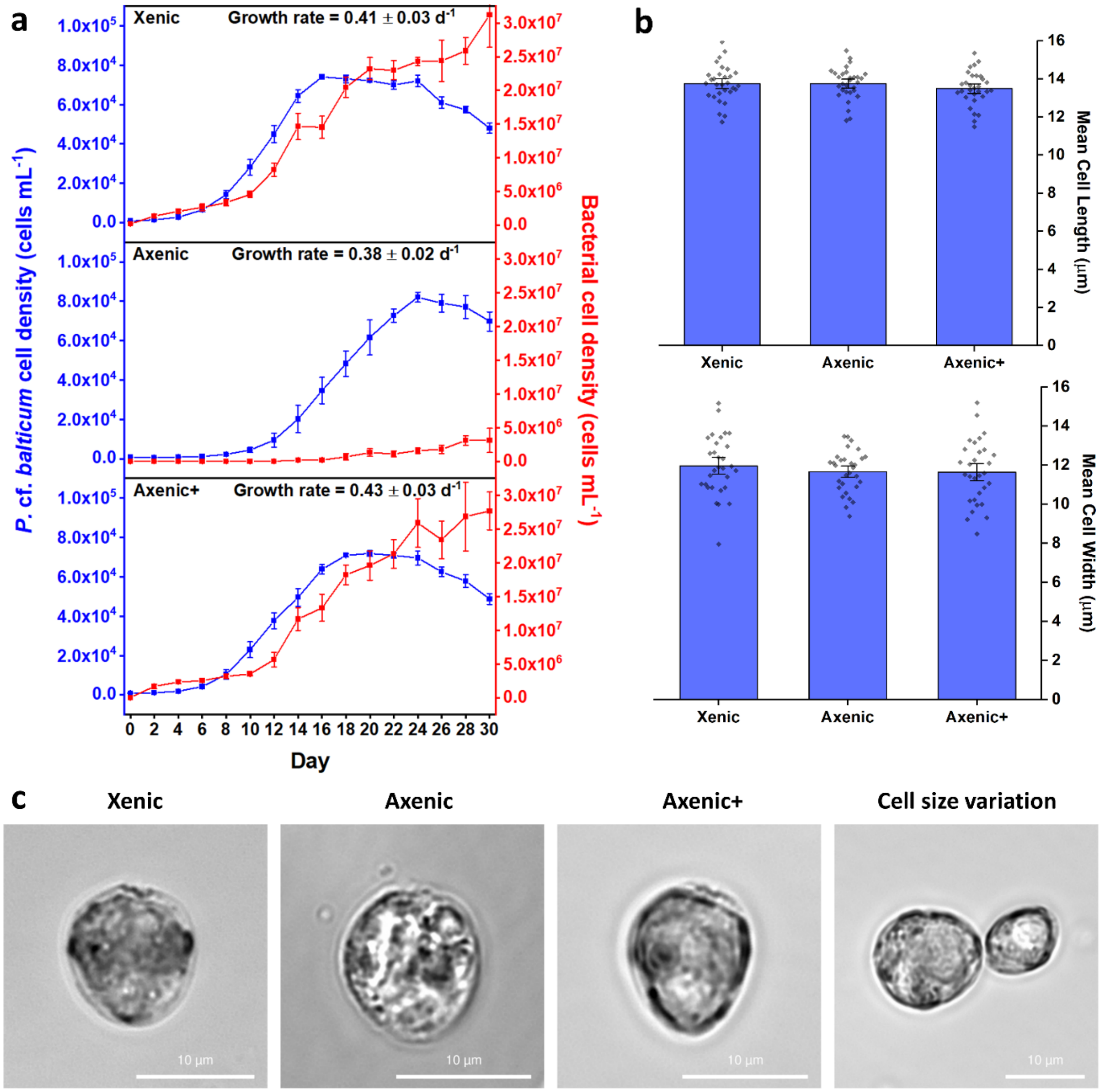

Supplementary Figure 14. (a) Growth curves of *P.* cf. *balticum* and associated bacteria for the xenic, axenic and axenic+ lineages demonstrating there was no effect of the antibiotic treatment on the overall fitness of the cells and that further work with the axenic strain can be considered representative of the original strain. Mean ± standard deviation (n=5 biological replicates). (b) Column graphs of mean cell size including both length and width are shown to demonstrate there was no change to the cell dimensions as a result of the antibiotic treatment but cell size variation as imaged was observed in all treatments. Mean ± standard error (n = 30 individual cells). (c) Shows representative differential interference contrast images of each variant taken using live cells on an upright fluorescence microscope (Nikon Eclipse Ni, Japan) with a mono camera (Nikon DS-Qi2) demonstrating very little change in the cell proportions due to antibiotic treatment but clear variation in cell size.

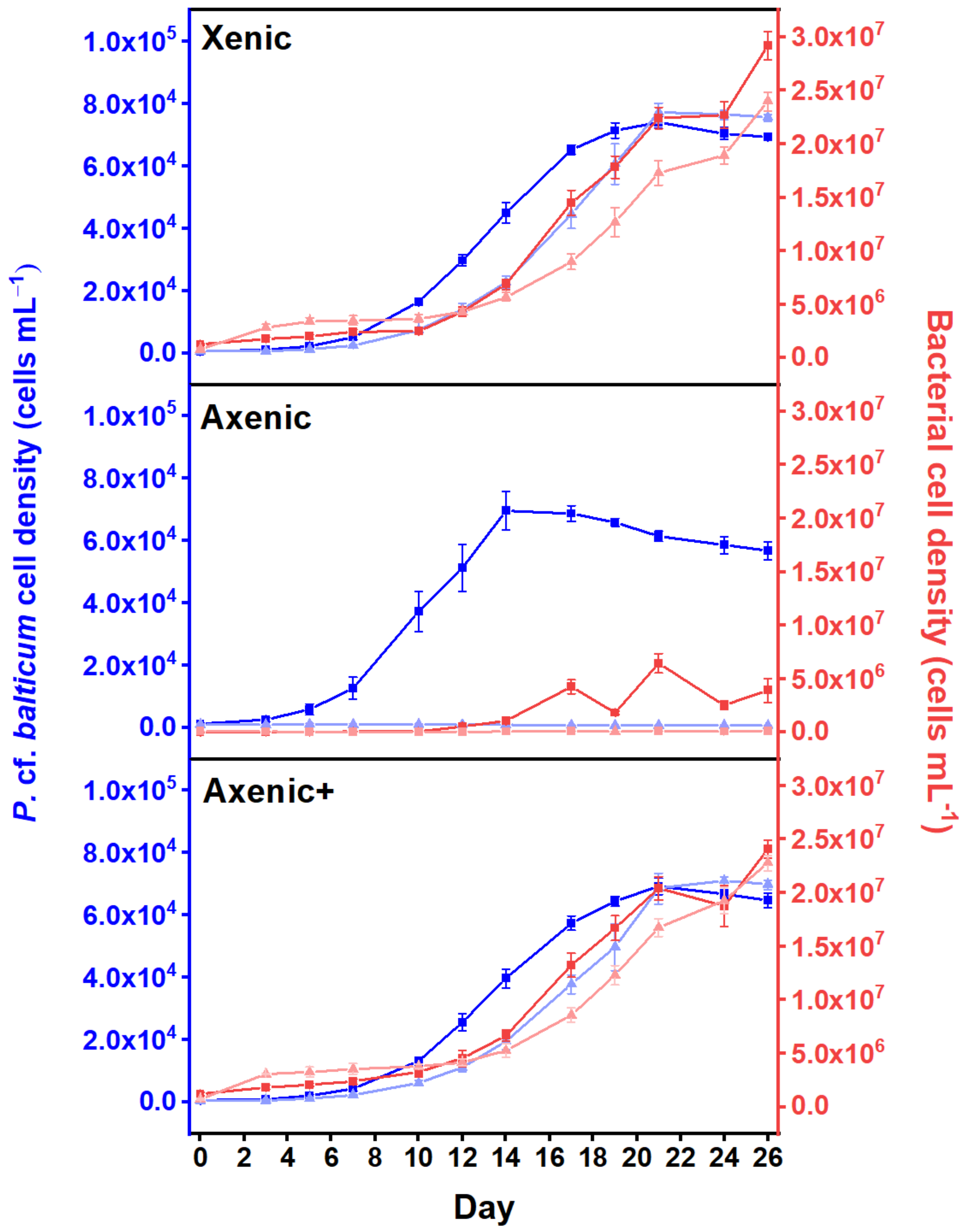

Supplementary Figure 15. Growth curve of *P.* cf. *balticum* grown with an organic (sodium β-glycerophosphate) (bright coloured square symbols) and inorganic (sodium phosphate) (pale coloured triangle symbols) form of phosphorus. In the absence of bacteria *P.* cf. *balticum* was not able to grow with organic phosphorus (axenic). Mean ± standard deviation (n=5 biological replicates).

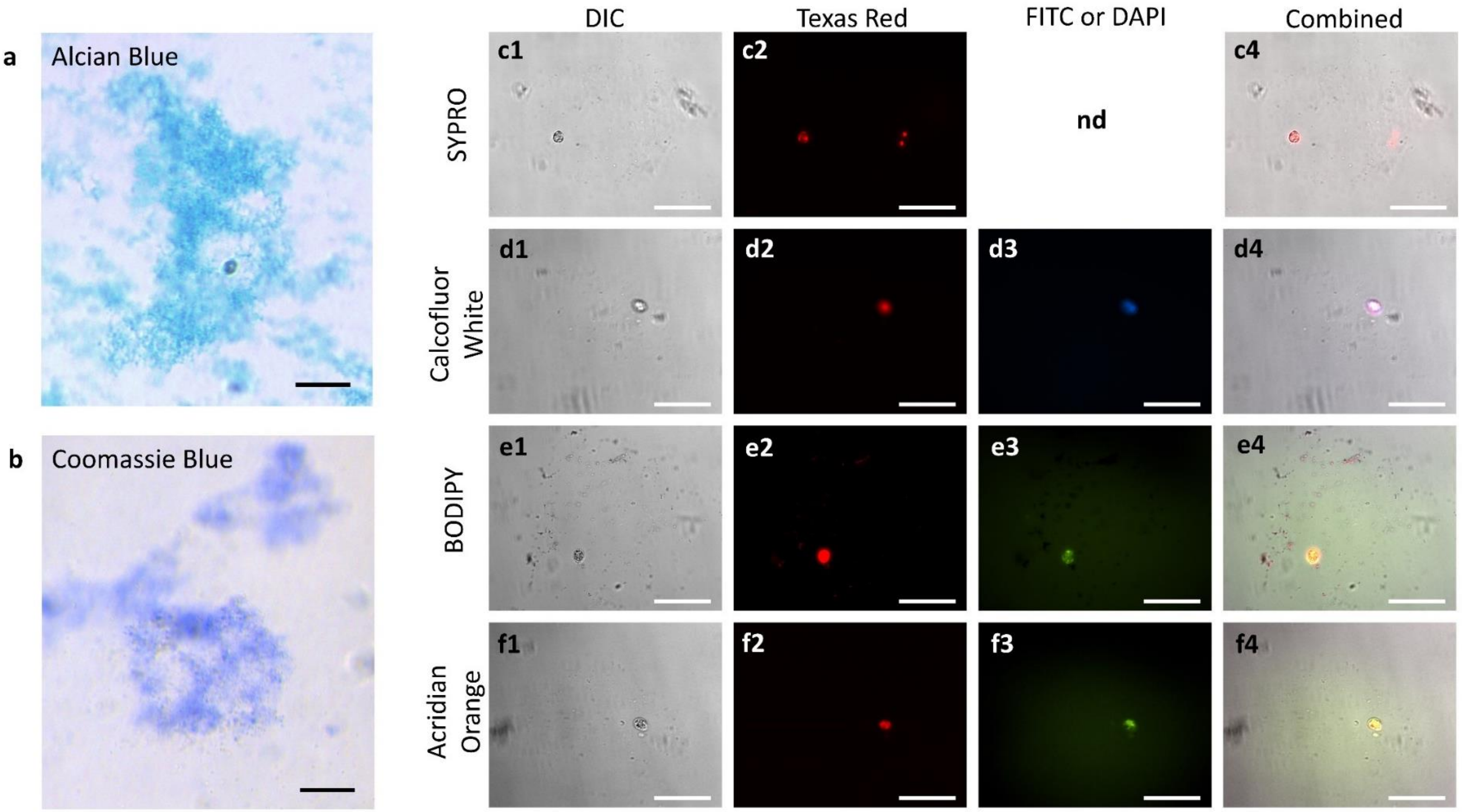

Supplementary Figure 16. Images of stains used to elucidate the composition of *P*. cf. *balticum* mucospheres including positive staining with (a) Alcian Blue and (b) Coomassie Brilliant Blue used to identify polysaccharide-rich and protein-rich transparent exopolymeric particles, respectively. The fluorescent stains used were (c) Sypro Red for proteins (note no data for FITC or DAPI channel), (d) Calcofluor White for β-polysaccharides, (e) BODIPY for neutral lipids and (f) Acridine Orange for mucopolysaccharides; all of which positively stained the *P*. cf. *balticum* cells, but not the mucospheres they produced. Images were taken on an inverted fluorescence microscope (Nikon Eclipse Ti), fitted with Nikon FITC 480/30nm ex 535/45nm em, Texas Red 560/40nm ex 630/60nm em and DAPI 375/28nm ex 460/60nm em filters and a black and white camera (Nikon DS-QiMc) under 200x magnification. Scale bar = 50 µm.

a

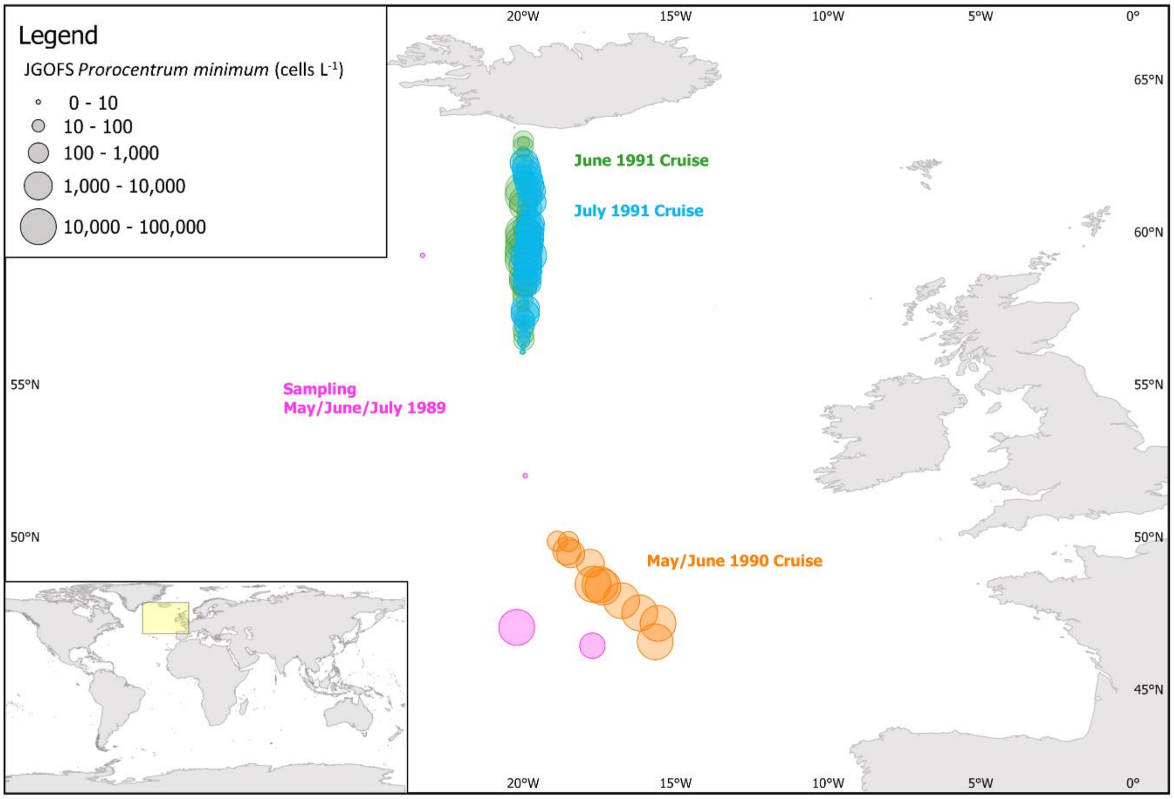
Legend
JGOFS *Prorocentrum minimum* (cells L^{-1})
0 - 10
10 - 100
100 - 1,000
1,000 - 10,000
10,000 - 100,000
June 1991 Cruise
July 1991 Cruise
Sampling May/June/July 1989
May/June 1990 Cruise
20°W
15°W
10°W
5°W
0°
65°N
60°N
55°N
50°N
45°N

b

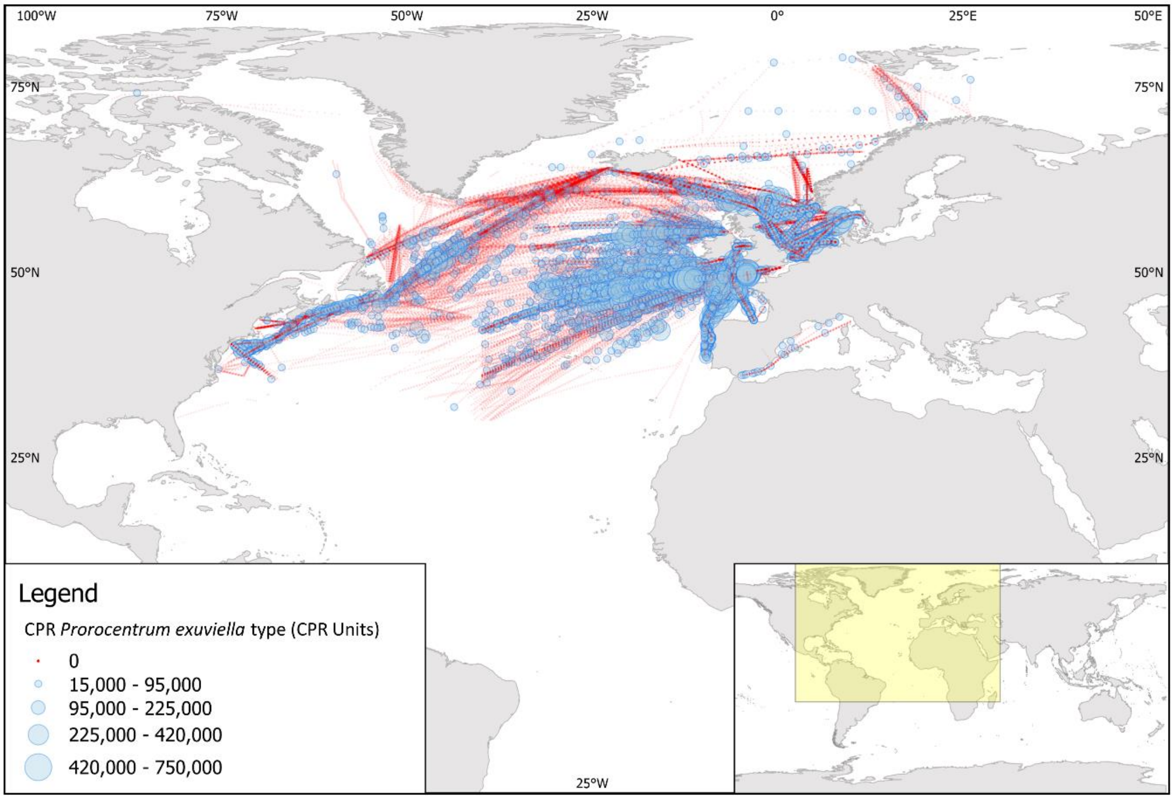
100°W
75°W
50°W
25°W
0°
25°E
50°E
75°N
50°N
25°N
Legend
CPR *Prorocentrum exuviella* type (CPR Units)
0
15,000 - 95,000
95,000 - 225,000
225,000 - 420,000
420,000 - 750,000

Supplementary Figure 17: Visualisation of two phytoplankton abundance datasets (enumerated using light microscopy) were analysed to determine a conservative appropriate *P.* cf. *balticum* cell abundance for calculating the potential contribution of carbon to the global ocean budget resulting from mucosphere production. (a) shows data collected as part of the Joint Global Ocean Flux Study (JGOFS) with the size of the coloured bubbles representing the cell abundance of *P.* cf. *balticum*-like cells (originally incorrected reported as *Prorocentrum minutum* then corrected to *P. minimum*) in cells L^{-1}. The different colours represent the different cruises included in this dataset; pink for May-July 1989; orange for May-June 1990; green for June 1991 and blue for July 1991. (b) shows data collected as part of the Continuous Plankton Recorder (CPR) from 1958 to 2018. The subset of data shown here is the category of *Prorocentrum* reported as "*Prorocentrum exuviaella* type" which includes species with the same morphology as *P.* cf. *balticum* (DOI:10.17031/1735). The red dots represent samples that were collected but *Prorocentrum exuviaella* type cells were not detected, and the size of the blue bubbles represent the abundance of *Prorocentrum exuviaella* type cells that were counted in CPR units.

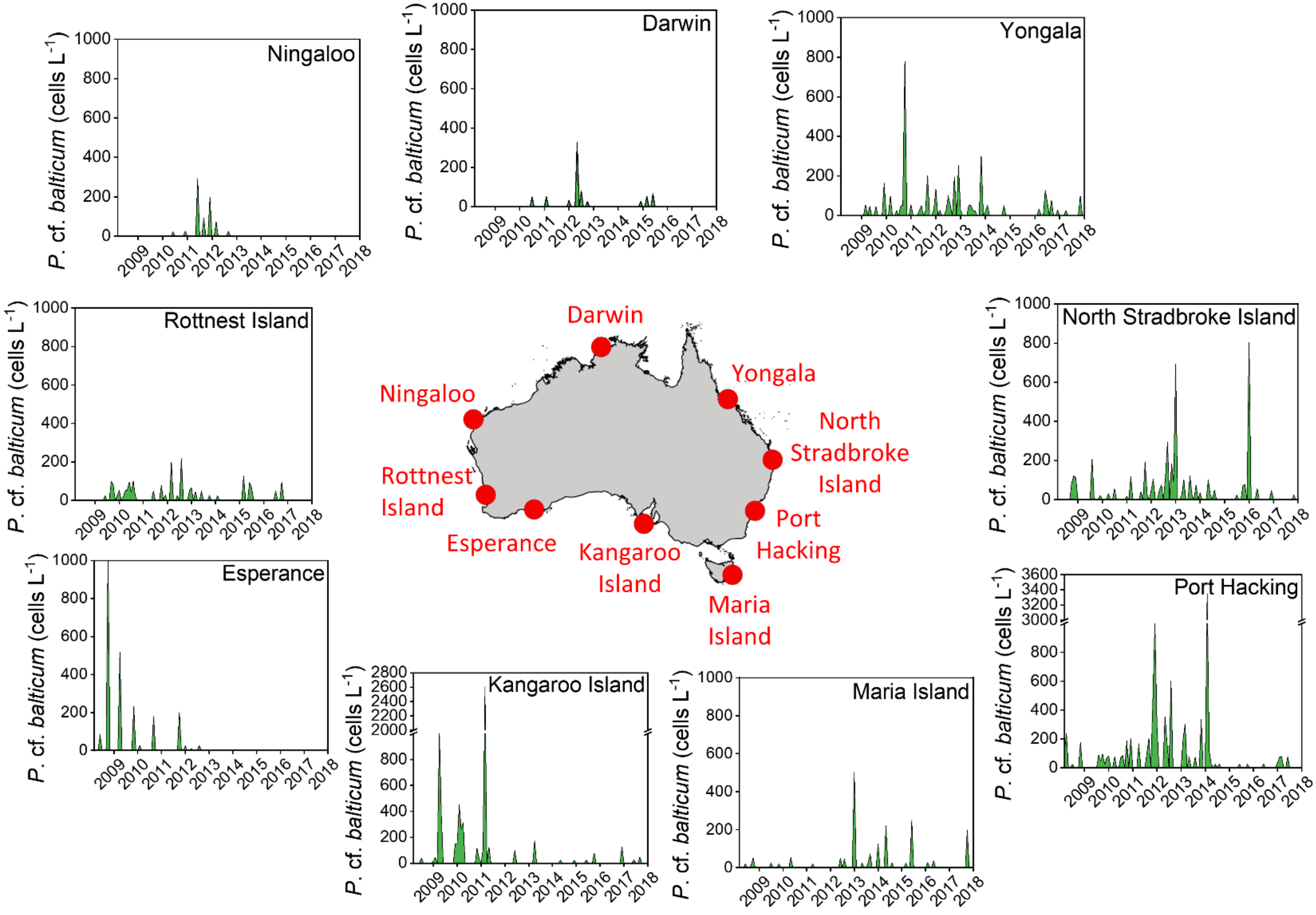

Supplementary Figure 18. Spatial and temporal distribution of *P*. cf. *balticum* and morphologically similar species from light microscope observations collected at nine Australian Integrated Marine Observing System National Reference Stations.

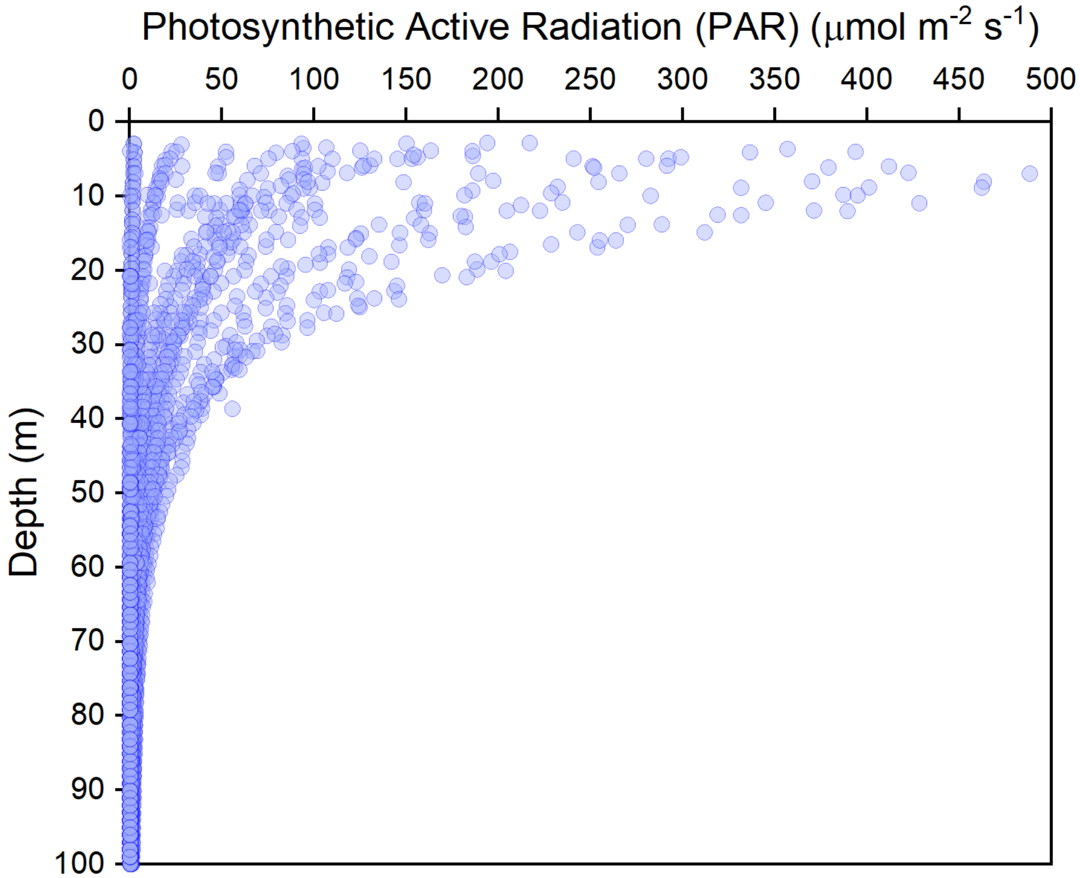

Supplementary Figure 19: Photosynthetic Active Radiation (PAR) (µmol m-2 s-1) measurements with depth (m) measured as part of the Tara Oceans initiative demonstrating light attenuates to 150 µmol m-2 s-1 (normal *P.* cf. *balticum* culture growth light conditions) and 20 µmol m-2 s-1 (low light growth conditions) at ~30 m and 60 m, respectively. Data redrawn from Picheral, et al. [1].

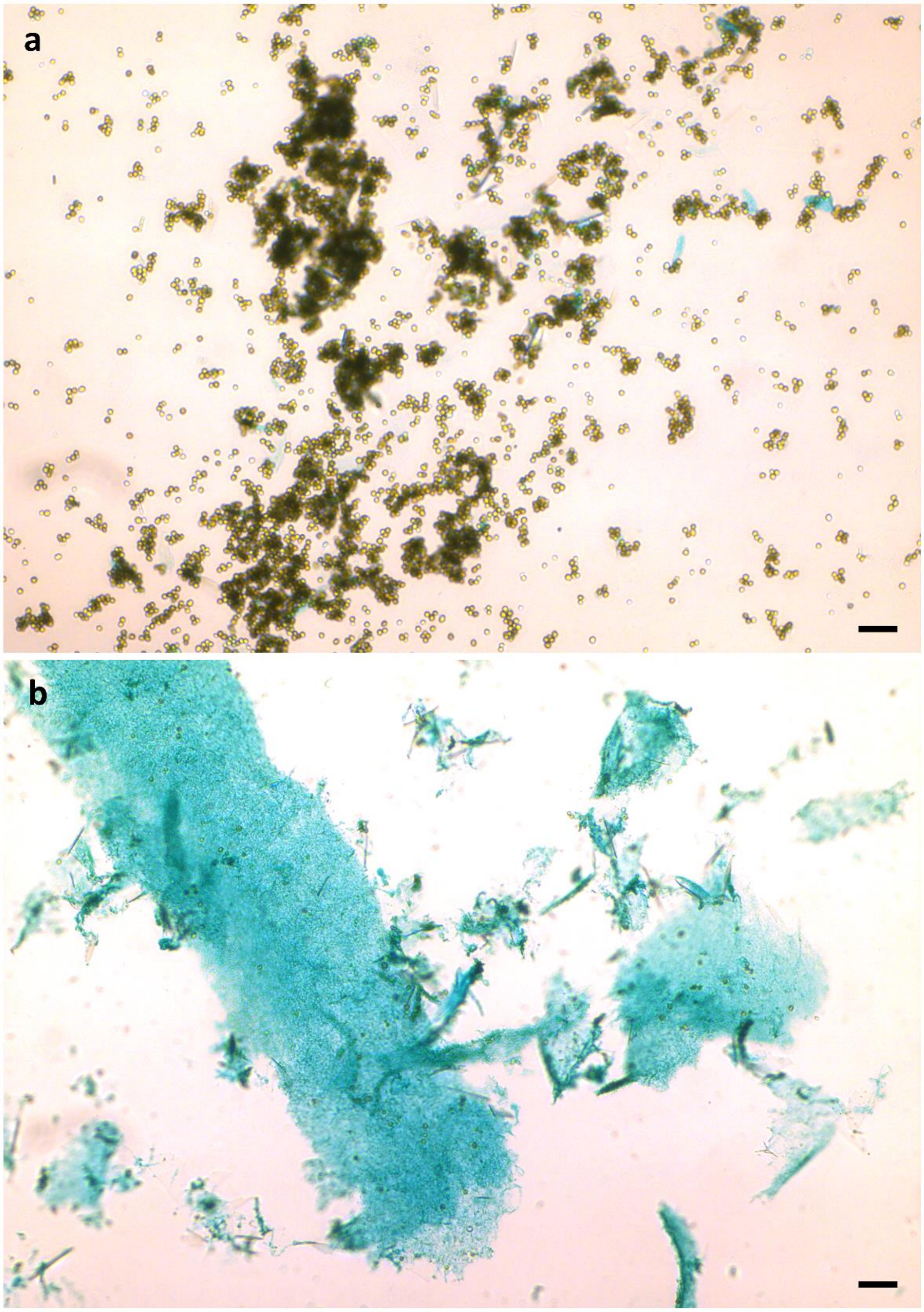

Supplementary Figure 20. Images showing the effective separation of *P.* cf. *balticum* (a) cells and (b) mucus using centrifugation. A 15 mL subsample of the axenic *P.* cf. *balticum* variant was vigorously shaken to remove the dinoflagellate cells from the mucospheres, then centrifuged at 1,000g for 5 mins to pellet the cells. The supernatant containing the mucus was then separated into another 15 mL tube and centrifuged again at 15,000g for 15 mins to pellet the mucus from the mucospheres and the supernatant removed. Both fractions were then stained with 0.02% 8GX Alcian Blue and visualised using an inverted light microscope (Nikon Eclipse TS100, Japan) under 100x magnification and imaged using a color camera (Infinity 1, Lumenera, Ontario, Canada). Scale bar = 100 µm.

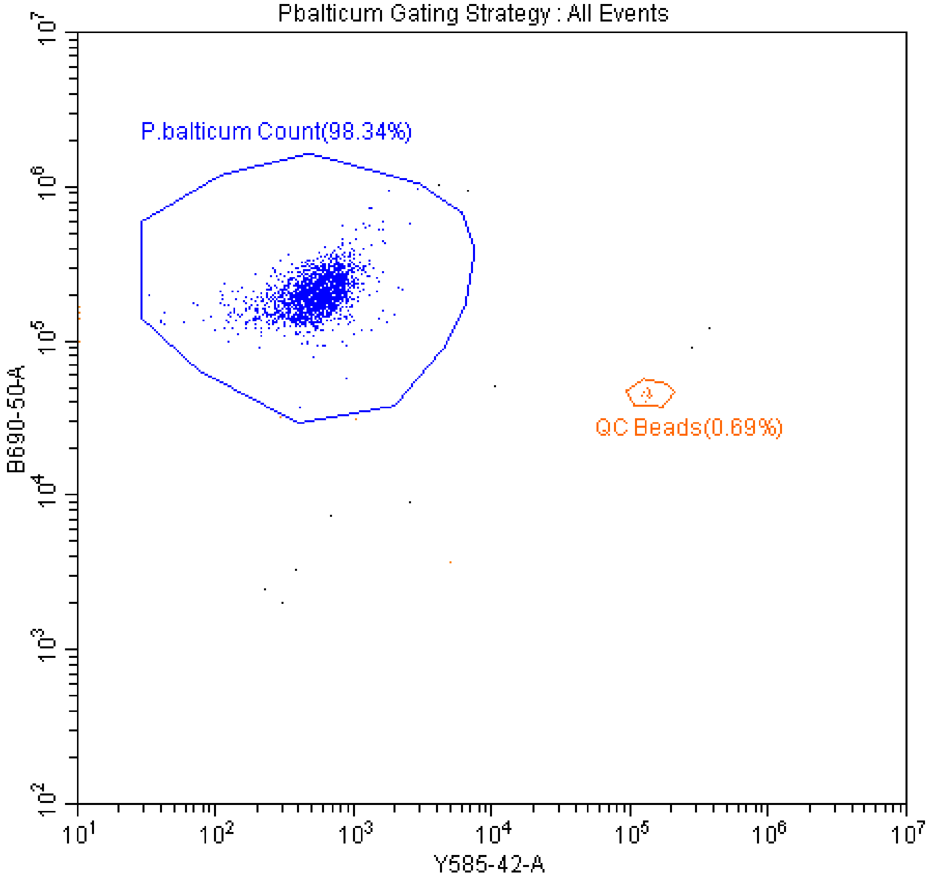

Supplementary Figure 21. Flow cytometer (Beckman Coulter Cytoflex LX with CytExpert v2.4 software, Indianapolis, USA) gating strategy used to detect and enumerate *Prorocentrum* cf. *balticum* cells during growth experiments. A blue laser (488 nm) excitation was used in combination with 690/50 nm and 585/42 nm detectors. Rainbow fluorescent QC beads (Cytoflex Daily QC Fluorospheres, BD Sciences) were also used to ensure accuracy of the settings and gating strategy between sampling days.

Supplementary Table 1. Description of the unicellular eukaryotic prey offered to *P*. cf. *balticum* for phago-heterotrophic consumption to test prey preferences.

Prey Species	Class	Cell Size (μm)	Evidence of phago-heterotrophic feeding
Amphidinium massartii	Dinophyceae	15	Yes
Coolia palmyrensis	Dinophyceae	25	Yes
Dunaliella tertiolecta	Chlorophyceae	10	Yes
Gambierdiscus lapillus	Dinophyceae	60	No
Gymnodinium catenatum	Dinophyceae	50	No
Nannochloropsis oceanica	Eustigmatophyceae	3	Yes
Porphyridium purpureum	Rhodophyceae	5	No
Prorocentrum lima	Dinophyceae	40	No
Prymnesium parvum	Prymnesiophyceae	12	Yes
Rhodomonas salina	Cryptophyceae	12	Yes
Proteomonas sulcata	Cryptophyceae	12	Yes
Scrippsiella sp.	Dinophyceae	15	No
Tetraselmis sp.	Chlorophyceae	15	Yes
Thalassiosira weissflogii	Bacillariophyceae	10	Yes
Thalassiosira pseudonana	Bacillariophyceae	5	Yes
Thalassiosira rotula	Bacillariophyceae	15	Yes
Tisochrysis lutea	Prymnesiophyceae	5	Yes

Supplementary Table 2. Mucosphere production rates by *P*. cf. *balticum* when grown under varied conditions with and without prokaryotic (natural microbiome) and axenic eukaryotic prey (*R. salina*). When mean values are reported, standard deviation is also shown.

Condition	Proportion of cells that produced mucospheres in 24 hr	Mean no. mucospheres cell^{-1} in 24 hr	Max no. mucospheres cell^{-1} in 24 hr	Mean time first mucosphere produced (hr)	Mean time first mucosphere abandoned (hr)	Mean hours spent in mucosphere (hr)	Max time spent in mucosphere (hr)	Min time spent in mucosphere (hr)
Axenic *P*. cf. *balticum* phosphate deplete	0% (n=26)	-	-	-	-	-	-	-
Axenic *P*. cf. *balticum* phosphate deplete + eukaryotic prey	46% (n=26)	1.1 (± 0.3) (n=12)	2 (n=12)	9.0 (± 4.9) (n=12)	21.2 (± 7.1) (n=10)[1]	14.4 (± 4.7) (n=10)[2]	>24 (n=12)	<4 (n=12)
Axenic *P*. cf. *balticum* phosphate deplete + prokaryotic prey	12% (n=26)	1.0 (± 0.0) (n=3)	1 (n=3)	24.0 (± 0.0) (n=3)	29.3 (± 2.3) (n=3)	5.3 (± 2.3) (n=3)	8 (n=3)	<4 (n=3)
Axenic *P*. cf. *balticum* phosphate deplete + eukaryotic and prokaryotic prey	69% (n=26)	1.1 (± 0.3) (n=18)	2 (n=18)	6.0 (± 4.8) (n=18)	16.8 (± 9.2) (n=18)	8.6 (± 6.1) (n=20)[2]	>16 (n=20)	<4 (n=20)
Axenic *P*. cf. *balticum* phosphate deplete in low light	0% (n=26)	-	-	-	-	-	-	-
Axenic *P*. cf. *balticum* phosphate deplete in low light + eukaryotic prey	23% (n=26)	1.0 (± 0.0) (n=6)	1 (n=6)	4.7 (± 1.6) (n=6)	18.7 (± 9.2) (n=3)[1]	13.3 (± 8.3) (n=3)[2]	>28 (n=6)	<4 (n=6)
Axenic *P*. cf. *balticum* phosphate replete	0% (n=26)	-	-	-	-	-	-	-
Axenic *P*. cf. *balticum* phosphate replete + eukaryotic prey	42% (n=26)	1.0 (n=11)	1 (n=11)	8.0 (± 5.8) (n=11)	18.0 (± 8.3) (n=8)[1]	11.5 (± 6.2) (n=8)[2]	>28 (n=8)	<4 (n=8)

Note: Mucosphere production was monitored at 0, 4, 8 hr after light exposure for two consecutive days and were grown on a 14:10 light:dark cycle.
[1] Some mucospheres were not abandoned during the monitoring period.
[2] It is not known how long the cells remained within the mucosphere between the 8 to 24 hr monitoring time points, though it was assumed to be the full 16 hr for this calculation.

Supplementary Table 3. Statistical significance of the mucosphere production experiments at the 24 hr timepoint tested using a Kruskal-Wallis H test, followed by a two-sided Mann-Whitney pairwise comparisons (with Bonferroni correction). Significant differences are shown in red text.

Kruskal-Wallis test	
H (chi2):	37.41
Hc (tie corrected):	67.82
p (same):	4.06E-12

There is a significant difference between sample medians

Two-sided Mann-Whitney pairwise (with Bonferroni corrected p values)

Treatments	Organic P	Organic P + prokaryote prey	Organic P + eukaryotic prey	Organic P + prokaryotic and eukaryotic prey	Inorganic P	Inorganic P + eukaryotic prey	Organic P and low light	Organic P and low light + eukaryotic prey
Organic P		1	1.16E-03	8.54E-06	1	0.03261	1	0.1426
Organic P + prokaryote prey	1		0.079	7.01E-04	1	1	1	1
Organic P + eukaryotic prey	1.16E-03	0.079		1	1.16E-03	1	1.16E-03	1
Organic P + prokaryotic and eukaryotic prey	8.54E-06	7.01E-04	1		8.54E-06	0.3757	8.54E-06	0.04393
Inorganic P	1	1	1.16E-03	8.54E-06		0.03261	1	0.1426
Inorganic P + eukaryotic prey	0.03261	1	1	0.3757	0.03261		0.03261	1
Organic P and low light	1	1	1.16E-03	8.54E-06	1	0.03261		0.1426
Organic P and low light + eukaryotic prey	0.1426	1	1	0.04393	0.1426	1	0.1426	

Supplementary information:

Supplementary Note 1. Detailed morphological and toxicological description of *P.* cf. *balticum*

The small (13-16 µm transdiameter) spheroid, laterally compressed dinoflagellate, is fast swimming, propelled by a longitudinal and transverse flagellum projecting from a single flagellar pore located anteriorly (Supplementary Fig. 1a), typical of Prorocentroid desmokonts ([2], p 402). Golden-brown chloroplasts are located at the periphery of cells (Fig. 1c) protected by a theca consisting of two halves or valves. Scanning Electron Microscopy (SEM) revealed the valves are covered in short spines (150 nm high), irregularly scattered small pores (150 nm diameter) and a small number of larger pores (330 nm diameter) located near the apical area, from which longer spines (600 nm high) emanate, and valves are joined by a distinct, ornamented intercalary band (Fig. 1d; Supplementary Fig. 1a-g). These morphological features and the presence of two wing-like apical projections bordering the periflagellar area (Fig. 1d; Supplementary Fig. 1b and e) align with the light microscope description of *Prorocentrum balticum* (Lohmann) Loeblich III (basionym *Exuviella baltica* Lohmann) by Lohmann [3] from the Baltic Sea, and the Scanning Electron Micrograph illustration from near the type locality ascribed to the same species by Elbrächter in Hoppenrath, et al. [4] (Fig. 78 m-n).

Phylogenetic analysis of ribosomal DNA from the internal transcribed spacer region (ITS) located between the 18S and 5.8S genes, the 28S large subunit (LSU) and the 18S small subunit (SSU) showed the strains to be indistinguishable from each other, though distinct from all genetically represented species within the genus *Prorocentrum* (Fig. 1a and Supplementary Fig. 2a-b). The strains grouped within a clade comprising the morphologically similar planktonic species *P. minimum* (=*P. cordatum*) and morphologically dissimilar species *P. dentatum*, *P. donghaiense* and *P. shikokuense* (=*P. obtusidens*) but were most closely related to taxonomically undefined strains (notation *Prorocentrum* sp. culture QUCCM SS1-13 from the Arabian Gulf and *P.* cf. *balticum*, culture CCMP 1787 from New Zealand, CCMP 1260 from Gulf of Mexico) (Fig. 1a and Supplementary Fig. 2a-b). Unfortunately, the original description of *P. balticum* by Lohmann [3] lacks critical detail of thecal plate ornamentation and no archived type material exists, nor are genetic sequences available for strains isolated from the type locality. Without a direct comparison, we cannot unequivocally determine if our strains are undescribed or are indeed authentic *P. balticum* and have therefore referred to them herein as *Prorocentrum* cf. *balticum*.

It is worth noting that the morphologically similar and phylogenetic sister species *Prorocentrum minimum*, has been linked with Harmful Algal Bloom (HAB) events and shown to produce a water-soluble neurotoxin found to cause detrimental effects to scallops, oysters, and clams[5], mortalities of fish, shellfish and zoobenthos

([6] and references therein), and death to mice[7]. Negative human health effects have also been reported, linked with the production of tetrodotoxin[8]. Subsequent studies have questioned whether production of the toxin was associated with the microalgal cells themselves, or a symbiotic bacterium[9]. Additional toxins associated with negative human health effects are also produced by other species from the genus *Prorocentrum*, including okadaic acid[10-12] and dinophysistoxins[10,11,13]. When tested using Liquid Chromatography-Tandem Mass Spectrometry (LC-MS/MS), our *P.* cf. *balticum* strains and their associated bacterial microbiome did not produce okadaic acid, dinophysistoxin-1 and -2, or tetrodotoxin, thus this species is unlikely to be associated with human illness or detrimental ecosystem impacts.

Supplementary Note 2. Nutrient limitation induced initiation of sexual reproduction

Phototrophy is a successful strategy for *P.* cf. *balticum* when dissolved inorganic nutrients are sufficient, and phago-heterotrophy an effective behaviour when they are not, but there can be occasions when dissolved inorganic nutrients and potential prey are both limiting. Under these conditions, *P.* cf. *balticum* can reproduce sexually, combining DNA to develop novel genotypes. Typically, growth is through asexual division via desmoschisis[14], but when grown under macronutrient (nitrogen and phosphorus) deplete conditions without prey, *P.* cf. *balticum* underwent sexual recombination. This process began with haploid isogamete development and pairing, followed by extraction of the nucleus from the donor through a fertilisation tube to the recipient gamete and conjugation of the nuclei within the recipient to form a planozygote. DNA was then replicated and the first and second meiotic divisions occurred resulting in a tetrad cell, finally producing four individual cells containing recombined DNA (Supplementary Fig. 5a-h; Supplementary Movie 1). This is an unusual form of sexual reproduction for dinoflagellates and has only been documented for one other species (*Prorocentrum micans*[15,16]) in which the description of the nuclei donation process through a fertilisation tube remarkably resembles the peduncular phago-heterotrophic feeding mechanism described within this study.

Supplementary Note 3. Phago-heterotrophic feeding mechanism

The phago-heterotrophic feeding mechanism of dinoflagellates varies widely but can be grouped into three main categories: (1) direct engulfment or phagocytosis, (2) pallium feeding and (3) peduncular feeding[17]. Phagocytosis involves the interception of a prey cell and complete engulfment via an opening in the dinoflagellate sulcal region[18-20] or the apical horn[19], and is mainly observed in, but not restricted to, unarmored dinoflagellates. In contrast, pallium and peduncular feeding are generally observed in armored dinoflagellates that are inhibited from ingesting large and complete prey through the narrow opening in the sulcal region[21].

Pallium feeding involves the capture of a potential prey item using a tow filament, followed by the extrusion of a pseudopodial "feeding veil" that engulfs and digests the prey outside of the dinoflagellate cell[22-24]. Similarly, peduncular feeding involves the capture of a potential prey item using a tow filament, extrusion of the peduncle, which penetrates the prey membrane and through which the cellular contents are extracted and deposited in food vacuoles for intracellular digestion within the dinoflagellate [24-26]. This process is also known as myzocytosis or "cellular vampirism"[27] and allows the consumption of prey items as large as 10 times the dinoflagellate's size[21].

Without direct observation of feeding, it is difficult to confirm the presence of a peduncle. On occasion, peduncle-like tubular organelles can be detected in SEM images (e.g., [28,29]) or inferred from the presence of intracellular microtubular baskets in Transmission Electron Microscope (TEM) images (e.g., [30,31]) and the presence of these have been proposed to indicate peduncular feeding capabilities of dinoflagellates in general [26]. Such structures have been reported for other Prorocentrales (e.g., *P. norrisianum* and *P. tropicalis* in[28]; *P. lima* in[32]; *P. micans* in [31,33,34], *P. arenarium* in [29], including the morphological and phylogenetically similar species *P. minimum* (as *P. mariae-lebouriae* in [31,35]). It has therefore been assumed that some *Prorocentrum* species feed via a peduncle, but this has never been observed until now. Curiously, the feeding mechanism for *P. micans*[19] and *P. minimum* has been reported as direct engulfment via separation of the intercalary band through the sutures between the two valves[19,36]. This contrasts with previous reports of microtubular structures in these species and the peduncular feeding we observed in *P.* cf. *balticum*.

Supplementary Note 4. Mixotroph classification

Further classification of mixotrophic protists differentiates between taxa with the innate ability to photosynthesise through vertical transmission of plastids, as opposed to those which acquire the ability through ingestion and domestication of phototrophic prey, known as constitutive and non-constitutive mixotrophs, respectively[37]. Constitutive mixotrophs can be further defined by their preference for phototrophy versus phago-heterotrophy[38,39]. Some protists are considered obligate phototrophs, acquiring carbon from photosynthesis, and supplementing nutrients through phago-heterotrophy (facultative phago-heterotroph) (defined as type II mixotrophs in[39]). While other protists are obligate phago-heterotrophs, only photosynthesising (facultative phototrophs) when prey are limiting (defined as type III mixotrophs in[39]).

Supplementary Note 5. Mucosphere composition

Mucus can be produced by microorganisms through a variety of mechanisms, but the most common method recognized for Prorocentrales, is secretion through pores in the thecal valves[2]. Equally, most pores in the thecal valves of Prorocentrales are associated with trichocysts, a type of extrusome that is involved in phago-heterotrophic feeding[21]. *P.* cf. *balticum* has two types of pores (Fig. 1d; Supplementary Fig. 1e), including a small number of large pores (330 nm diameter) located near the apical area from which large spines emanate, a feature that is unique to the strains described in our study. Trichocysts and mucocysts were not observed but it is possible that this unique morphological characteristic in *P.* cf. *balticum* is associated with the release of the mucoid material that is used to construct its distinctive mucospheres. Effort was made to further characterise the chemical composition of the mucospheres by staining with a series of fluorescent stains including SYPRO-Red for proteins[40], Calcofluor white for β-polysaccharides[41], BODIPY for neutral lipids[42] and Acridine Orange for mucopolysaccharides[43] but all were negative (Supplementary Fig. 15c-f).

Supplementary References:

1 Picheral, M. *et al.* (PANGAEA, 2014).
2 Tomas, C. R. *Identifying Marine Phytoplankton*. (Academic Press, 1997).
3 Lohmann, H. v. Untersuchungen zur Feststellung des vollstandigen Gehaltes des Meeres an Plankton. *Wissenschaftl. Meeresunters. Abt. Kiel* **10**, 129-370 (1908).
4 Hoppenrath, M., Elbrächter, M. & Drebes, G. *Marine phytoplankton. Selected microphytoplankton species from the North Sea around Helgoland and Sylt , Schweitzerbarth Verlag, Stuttgart*. (Kleine Senckenberg-Reihe, 2009).
5 Glibert, P. M., Alexander, J., Meritt, D. W., North, E. W. & Stoecker, D. K. Harmful algae pose additional challenges for oyster restoration: impacts of the harmful algae *Karlodinium veneficum* and *Prorocentrum minimum* on early life stages of the oysters *Crassostrea virginica* and *Crassostrea ariakensis*. *Journal of Shellfish Research* **26**, 919-925 (2007).
6 Heil, C. A., Glibert, P. M. & Fan, C. *Prorocentrum minimum* (Pavillard) Schiller: a review of a harmful algal bloom species of growing worldwide importance. *Harmful Algae* **4**, 449-470 (2005).
7 Grzebyk, D., Denardou, A., Berland, B. & Pouchus, Y. Evidence of a new toxin in the red-tide dinoflagellate *Prorocentrum minimum*. *Journal of Plankton Research* **19**, 1111-1124 (1997).
8 Vlamis, A. *et al.* First detection of tetrodotoxin in Greek shellfish by UPLC-MS/MS potentially linked to the presence of the dinoflagellate *Prorocentrum minimum*. *Toxins* **7**, 1779-1807 (2015).
9 Rodríguez Filgueiras, I. *et al.* The association of bacterial C9-based TTX-like compounds with *Prorocentrum minimum* opens new uncertainties about shellfish seafood safety. (2017).
10 Hu, T. *et al.* in *Toxic Phytoplankton Blooms in the Sea* (eds T. J. Smayda & Y. Shimizu) (Elsevier Science Publish, 1993).
11 Morton, S. L. Morphology and toxicology of *Prorocentrum faustiae* sp. nov., a toxic species of non-planktonic dinoflagellate from Heron Island, Australia. *Botanica Marina* **41**, 565-570 (1998).
12 Murakami, Y., Oshima, Y. & Yasumoto, Y. Identification of okadaic acid as a toxic component of a marine dinoflagellate *Prorocentrum lima*. *Bulletin of the Japanese Society for the Science and Fish* **48**, 69-72 (1982).
13 Lee, J.-S. *et al.* Determination of diarrhetic shellfish toxins in various dinoflagellate species. *Journal of Applied Phycology* **1**, 147-152 (1989).

14 *Marine benthic dinoflagellates-unveiling their worldwide biodiversity*. (Kleine Senckenberg-Reihe, 2014).
15 Bhaud, Y., Soyer-Gobillard, M.-O. & Salmon, J. Transmission of gametic nuclei through a fertilization tube during mating in a primitive dinoflagellate, *Prorocentrum micans* Ehr. *Journal of Cell Science* **89**, 197-206 (1988).
16 Soyer-Gobillard, M., Bhaud, Y. & Saint Hilaire, D. New data on mating in an autotrophic dinoflagellate, *Prorocentrum micans* Ehrenberg. *Vie et milieu* **52**, 167-176 (2002).
17 Jacobson, D. M. & Anderson, D. M. Thecate heterophic dinoflagellates: feeding behavior and mechanisms. *Journal of Phycology* **22**, 249-258 (1986).
18 Skovgaard, A. Engulfment of *Ceratium* spp. (Dinophyceae) by the thecate photosynthetic dinoflagellate *Fragilidium subglobosum*. *Phycologia* **35**, 490-499 (1996).
19 Jeong, H. J. *et al.* Feeding by phototrophic red-tide dinoflagellates: five species newly revealed and six species previously known to be mixotrophic. *Aquatic microbial ecology* **40**, 133-150 (2005).
20 Jeong, H. J. *et al.* Feeding by the mixotrophic red-tide dinoflagellate *Gonyaulax polygramma*: mechanisms, prey species, effects of prey concentration, and grazing impact. *Aquatic Microbial Ecology* **38**, 249-257 (2005).
21 Hansen, P. J. & Calado, A. J. Phagotrophic mechanisms and prey selection in free-living dinoflagellates. *Journal of Eukaryotic Microbiology* **46**, 382-389 (1999).
22 Gaines, G. & Taylor, F. Extracellular digestion in marine dinoflagellates. *Journal of Plankton Research* **6**, 1057-1061 (1984).
23 Jacobson, D. M. & Anderson, D. M. Ultrastructure pf the feeding appartus and myonemal system of the heterotrophic dinoflagellate *Protoperidinium spinulosum*. *Journal of Phycology* **28**, 69-82 (1992).
24 Hansen, P. J. Prey size selection, feeding rates and growth dynamics of heterotrophic dinoflagellates with special emphasis on *Gyrodinium spirale*. *Marine Biology* **114**, 327-334 (1992).
25 Calado, A. & Moestrup, Ø. Feeding in *Peridiniopsis berolinensis* (Dinophyceae): new observations on tube feeding by an omnivorous, heterotrophic dinoflagellate. *Phycologia* **36**, 47-59 (1997).
26 Spero, H. J. Phagotrophy in *Gymnodinium fungiforme* (Pyrrhophyta): the peduncle as an organelle of ingestion. *Journal of Phycology* **18**, 356-360 (1982).
27 Gaines, G. & Elbrächter, M. *Heterotrophic nutrition. The biology of dinoflagellates.*, (Oxford, Blackwell Scientific, 1987).
28 Faust, M. A. Three new benthic species of *Prorocentrum* (Dinophyceae) from Belize : *P. norrisianum* sp. nov., *P. tropicalis* sp. nov., and *P. reticulatum* sp. nov. *Journal of Phycology* **33**, 851-858 (1997).
29 Faust, M. A. Three new benthic species of *Prorocentrum* (Dinophyceae) from Carrie Bow Cay, Belize: *P. sabulosum* sp. nov., *P. sculptile* sp. nov., and *P. arenarium* sp. nov. *Journal of phycology* **30**, 755-763 (1994).
30 Schnepf, E., Deichgräber, G. & Drebes, G. Food uptake and the fine structure of the dinophyte *Paulsenella* sp. an ectoparasite of marine diatoms. *Protoplasma* **124**, 188-204 (1985).
31 Roberts, K. R., Heimann, K. & Wetherbee, R. The flagellar apparatus and canal structure in *Prorocentrum micans* (Dinophyceae). *Phycologia* **34**, 313-322 (1995).
32 Malcolm, S. Aspects of the biology and ultrastructure of *Prorocentrum* spp. (pyrrhophyta). (1987).
33 Schnepf, E. & Winter, S. A microtubular basket in the armoured dinoflagellate *Prorocentrum micans* (Dinophyceae). *Archiv für Protistenkunde* **138**, 89-91 (1990).
34 Dodge, J. D. & Bibby, B. T. The Prorocentrales (Dinophyceae): I. A comparative account of fine structure in the genera *Prorocentrum* and *Exuviaella*. *Botanical Journal of the Linnean Society* **67**, 175-187 (1973).
35 Faust, M. A. Micromorphology of a small dinoflagellate *Prorocentrum mariae-lebouriae* (Park & Ballantine) comb. nov. *Journal of Phycology* **10**, 315-322 (1974).
36 Johnson, M. D. Inducible mixotrophy in the dinoflagellate *Prorocentrum minimum*. *Journal of Eukaryotic Microbiology* **62**, 431-443 (2015).
37 Mitra, A. *et al.* Defining planktonic protist functional groups on mechanisms for energy and nutrient acquisition: incorporation of diverse mixotrophic strategies. *Protist* **167**, 106-120 (2016).

38 Stoecker, D. K., Hansen, P. J., Caron, D. A. & Mitra, A. Mixotrophy in the marine plankton. *Annual Review of Marine Science* **9**, 311-335 (2017).
39 Stoecker, D. K. Conceptual models of mixotrophy in planktonic protists and some ecological and evolutionary implications. *European Journal of Protistology* **34**, 281-290 (1998).
40 Zubkov, M. V., Fuchs, B. M., Eilers, H., Burkill, P. H. & Amann, R. Determination of total protein content of bacterial cells by SYPRO staining and flow cytometry. *Applied and Environmental Microbiology* **65**, 3251-3257 (1999).
41 Gonzalez-Machado, C., Capita, R., Riesco-Pelaez, F. & Alonso-Calleja, C. Visualization and quantification of the cellular and extracellular components of *Salmonella agona* biofilms at different stages of development. *PLoS One* **13**, e0200011 (2018).
42 Rumin, J. *et al.* The use of fluorescent Nile red and BODIPY for lipid measurement in microalgae. *Biotechnology for biofuels* **8**, 42 (2015).
43 Coats, D. W. & Heinbokel, J. F. A study of reproduction and other life cycle phenomena in planktonic protists using an acridine orange fluorescence technique. *Marine Biology* **67**, 71-79 (1982).
