# Supplementary material for: Mucospheres produced by a mixotrophic protist impact ocean carbon cycling
Source: Nat Commun. 2022 Mar 14;13:1301. doi: 10.1038/s41467-022-28867-8 (PMC8921327; doi:10.1038/s41467-022-28867-8)
Supplement: Supplementary file 2 — Description of Additional Supplementary Files [file 41467_2022_28867_MOESM2_ESM.pdf]

Description of Additional Supplementary Files

File Name: Supplementary Data 1
Description: Spreadsheet detailing how the carbon export potential of mucospheres produced by P. cf. balticum were calculated.

File Name: Supplementary Movie 1
Description: Evidence of mucosphere production, prey capture and peduncular phago-heterotrophic consumption by P. cf. balticum.
