# Supplementary material for: Mucospheres produced by a mixotrophic protist impact ocean carbon cycling
Source: Nat Commun. 2022 Mar 14;13:1301. doi: 10.1038/s41467-022-28867-8 (PMC8921327; doi:10.1038/s41467-022-28867-8)
Supplement: Supplementary file 5 — Reporting Summary [file 41467_2022_28867_MOESM5_ESM.pdf]

nature research

Corresponding author(s): Dr. Michaela E. Larsson

Last updated by author(s): Dec 7, 2021

Reporting Summary

Nature Research wishes to improve the reproducibility of the work that we publish. This form provides structure for consistency and transparency in reporting. For further information on Nature Research policies, see our Editorial Policies and the Editorial Policy Checklist.

Statistics

For all statistical analyses, confirm that the following items are present in the figure legend, table legend, main text, or Methods section.

n/a	Confirmed	
☐	☒	The exact sample size (*n*) for each experimental group/condition, given as a discrete number and unit of measurement
☐	☒	A statement on whether measurements were taken from distinct samples or whether the same sample was measured repeatedly
☐	☒	The statistical test(s) used AND whether they are one- or two-sided<br>*Only common tests should be described solely by name; describe more complex techniques in the Methods section.*
☒	☐	A description of all covariates tested
☐	☒	A description of any assumptions or corrections, such as tests of normality and adjustment for multiple comparisons
☐	☒	A full description of the statistical parameters including central tendency (e.g. means) or other basic estimates (e.g. regression coefficient) AND variation (e.g. standard deviation) or associated estimates of uncertainty (e.g. confidence intervals)
☐	☒	For null hypothesis testing, the test statistic (e.g. *F*, *t*, *r*) with confidence intervals, effect sizes, degrees of freedom and *P* value noted<br>*Give P values as exact values whenever suitable.*
☐	☒	For Bayesian analysis, information on the choice of priors and Markov chain Monte Carlo settings
☒	☐	For hierarchical and complex designs, identification of the appropriate level for tests and full reporting of outcomes
☒	☐	Estimates of effect sizes (e.g. Cohen's *d*, Pearson's *r*), indicating how they were calculated

Our web collection on statistics for biologists contains articles on many of the points above.

Software and code

Policy information about availability of computer code

Data collection	Cytexpert v2.4 was used for flow cytometric cell enumeration; Infinity Analyze v6.4.0 or NIS Elements v4.60 was used for light, fluorescence or confocal microscopy to visualise, image, and video cells.
Data analysis	Phylogenetic analysis were performed using Geneious Prime v1.8.032; MUSCLE algorithm; Maximum Likelihood trees were generated using PHYLM; Bayesian analysis was performed using MrBayes.<br>Prorocentrum cf. balticum associated microbiome was analysed using R v3.6.3 and R packages, dada2 v1.14.0, phyloseq v1.30.0, vegan and the SILVA database v138.<br>Biogeographic distribution maps were made in QGIS v2.18.16.<br>Software used with microscopes was Infinity Analyse v6.4.0 and NIS elements v4.60.

For manuscripts utilizing custom algorithms or software that are central to the research but not yet described in published literature, software must be made available to editors and reviewers. We strongly encourage code deposition in a community repository (e.g. GitHub). See the Nature Research guidelines for submitting code & software for further information.

Data

Policy information about availability of data

All manuscripts must include a data availability statement. This statement should provide the following information, where applicable:

- Accession codes, unique identifiers, or web links for publicly available datasets
- A list of figures that have associated raw data
- A description of any restrictions on data availability

The xenic and axenic Prorocentrum cf. balticum lineages established in this study have been deposited at the Australian National Algal Culture Collection (ANACC) under accession CS-1390 and are available for distribution. Sequences from the phylogenetic analysis of the four Prorocentrum cf. balticum strains have been

deposited in Genbank with accession numbers LSU MW024106-MW02409; SSU MW024110-MW024113; ITS1 MW024089-MW02492 and SSU-ITS-LSU MW024115-MW024118. The raw .fastq read files from the Prorocentrum cf. balticum associated microbiome assessment were deposited in Sequence Read Archive (SRA) under project id PRJNA737517 with sample numbers SAMN19697965-SAMN19697985. Raw data associated with the mucosphere carbon contribution calculations are supplied as a supplementary excel file titled Supplementary Data File 1. A movie file showing phago-heterotrophic feeding and mucosphere construction is provided as a supplementary .mp4 file titled Supplementary Movie File 1. The data used to assess the distribution and abundance of Prorocentrum cf. balticum can be accessed the following links: Integrated Marine Observing System National Reference Station Program (IMOS-NRSP), through the Australia Ocean Data Network (AODN) portal https://portal.aodn.org.au/; Joint Global Ocean Flux Study (JGOFS), DOI:10.1594/PANGAEA.859221; Continuous Plankton Recorder (CPR) Survey, DOI:10.17031/1735; The Tara Oceans amplicon dataset, DOI:10.1594/PANGAEA.873275 and DOI:10.1594/PANGAEA.875582

Field-specific reporting

Please select the one below that is the best fit for your research. If you are not sure, read the appropriate sections before making your selection.

☐ Life sciences ☐ Behavioural & social sciences ☒ Ecological, evolutionary & environmental sciences

For a reference copy of the document with all sections, see nature.com/documents/nr-reporting-summary-flat.pdf

Ecological, evolutionary & environmental sciences study design

All studies must disclose on these points even when the disclosure is negative.

Study description	We combine microscopic observations of the undescribed foraging behaviour of a novel mixotrophic dinoflagellate, with manipulative experiments and a biogeographical assessment of its distribution, to quantify its major contribution to the vertical flux of carbon from the euphotic zone of the ocean. All experiments were single-factor designs, carried out with a minimum of 3 replicates. See "Statistics and Reproducibility" section.
Research sample	The research samples comprised of clonal microalgal strains isolated from natural oceanic plankton communities originating from the continental shelf waters off south east Australia. The clonal strains identified as a novel dinoflagellate species are referred to as Prorocentrum cf. balticum and are available from the Australian National Algal Culture Collection (ANACC) under accession CS-1390. Refer to Methods section for more detail.
Sampling strategy	Samples were originally collected from oceanic surface waters using a 20 µm plankton net, from which clonal (single cell) isolates were established. For details of this process and subsequent experiment sampling strategies, please refer to the Methods.
Data collection	Various data collection methods were used in this study and are detailed in the Methods.
Timing and spatial scale	The plankton net tow was conducted in September 2018 from which the clonal dinoflagellates cultures were isolated and established.
Data exclusions	No data was excluded from the analysis.
Reproducibility	Mucosphere production was reproduced on many occasions throughout the study period. See "Statistics and Reproducibility" section for details of each experiment.
Randomization	Randomization was used for each experiment with a random collection of cells used to represent the population in each instance.
Blinding	Blinding was not relevant to this study.

Did the study involve field work? ☒ Yes ☐ No

Field work, collection and transport

Field conditions	Sampling occurred in September 2018 (Austral Spring) during fine conditions.
Location	Sampling was part of Australia's Integrated Marine Observing System National Reference Station sampling program at Port Hacking (34.120°S, 151.224°E).
Access & import/export	Samples were collected using a 20 µm plankton net from the local site through the national sampling strategy therefore access, import and export permits were not required.
Disturbance	No disturbance was caused by oceanographic sample collection.

Reporting for specific materials, systems and methods

We require information from authors about some types of materials, experimental systems and methods used in many studies. Here, indicate whether each material, system or method listed is relevant to your study. If you are not sure if a list item applies to your research, read the appropriate section before selecting a response.

Materials & experimental systems

n/a	Involved in the study
x	Antibodies
x	Eukaryotic cell lines
x	Palaeontology and archaeology
	x Animals and other organisms
x	Human research participants
x	Clinical data
x	Dual use research of concern

Methods

n/a	Involved in the study
x	ChIP-seq
	x Flow cytometry
x	MRI-based neuroimaging

Animals and other organisms

Policy information about studies involving animals; ARRIVE guidelines recommended for reporting animal research

Laboratory animals	This study did not involve laboratory animals.
Wild animals	This study did not involve wild animals.
Field-collected samples	Plankton was collected from the field using a 20 μm net and stored in a sealed bottle until returning to the lab. Clonal microalgal strains were then isolated from the collected material and stored in an incubator under the conditions described in the Methods.
Ethics oversight	No ethical approval or guidance was required for this study because the study organisms are non-hazardous microbes.

Note that full information on the approval of the study protocol must also be provided in the manuscript.

Flow Cytometry

Plots

Confirm that:

- [x] The axis labels state the marker and fluorochrome used (e.g. CD4-FITC).
- [x] The axis scales are clearly visible. Include numbers along axes only for bottom left plot of group (a 'group' is an analysis of identical markers).
- [x] All plots are contour plots with outliers or pseudocolor plots.
- [x] A numerical value for number of cells or percentage (with statistics) is provided.

Methodology

Sample preparation	Microalgal cell enumeration - there was no sample preparation required for enumerating microalgal cells using flow cytometry.<br>Bacterial cell enumeration - samples were stained with SYBR Green as described in Methods.
Instrument	Beckman Coulter Cytoflex LX
Software	Cytexpert v2.4
Cell population abundance	Cell population abundance varied between experiments, treatments and samples.
Gating strategy	Microalgal cell enumeration - the gating strategy used the chl-a autofluorescence to identify cells to be gated, was activated using the blue laser (488 nm) excitation with 690/50 nm and 585/42 nm detection.<br>Bacterial cell enumeration - the gating strategy used SYBR Green to stain the DNA of cell for detection. Cells were detected using the blue laser (488nm) excitation with 525/40 nm and Violet SSC detection.

- [x] Tick this box to confirm that a figure exemplifying the gating strategy is provided in the Supplementary Information.
